# Copper(II)-Dioxygen Facilitated Activation of Nitromethane: Nitrogen Donors for the Synthesis of Substituted 2-Hydroxyimino-2-phenylacetonitriles and Phthalimides

**DOI:** 10.3389/fchem.2020.622867

**Published:** 2021-01-29

**Authors:** Shiqun Xiang, Yinghua Li, Weibin Fan, Jiang Jin, Wei Zhang, Deguang Huang

**Affiliations:** ^1^ State Key Laboratory of Structural Chemistry, Fujian Institute of Research on the Structure of Matter, Chinese Academy of Sciences, Fuzhou, China; ^2^ School of Chemical Sciences, University of Chinese Academy of Sciences, Beijing, China

**Keywords:** copper-dioxygen, nitrogen source, nitromethane transformation, methyl nitrite, phthalimide, 2-hydroxyimino-2-phenylacetonitrile

## Abstract

A simple and efficient method is explored for the synthesis of 2-hydroxyimino-2-phenylacetonitriles (2) and phthalimides (4), by using nitromethane as nitrogen donors. Both reactions are promoted by Cu(II) system with the participation of dioxygen as an oxidant. The scope of the method has been successfully demonstrated with a total of 51 examples. The flexible and diversified characteristics of reactions are introduced in terms of electronic effect, steric effect, position of substituted groups, and intramolecular charge transfer. Experimental studies suggest that the methyl nitrite could be a precursor in the path to the final products. A possible reaction mechanism is proposed, including the Cu(II)/O_2_-facilitated transformation of nitromethane to methyl nitrite, the base-induced formation of 2-hydroxyimino-2-phenylacetonitriles, and the base-dioxygen-promoted formation of phthalimides.

## Introduction

**SCHEME 1:**
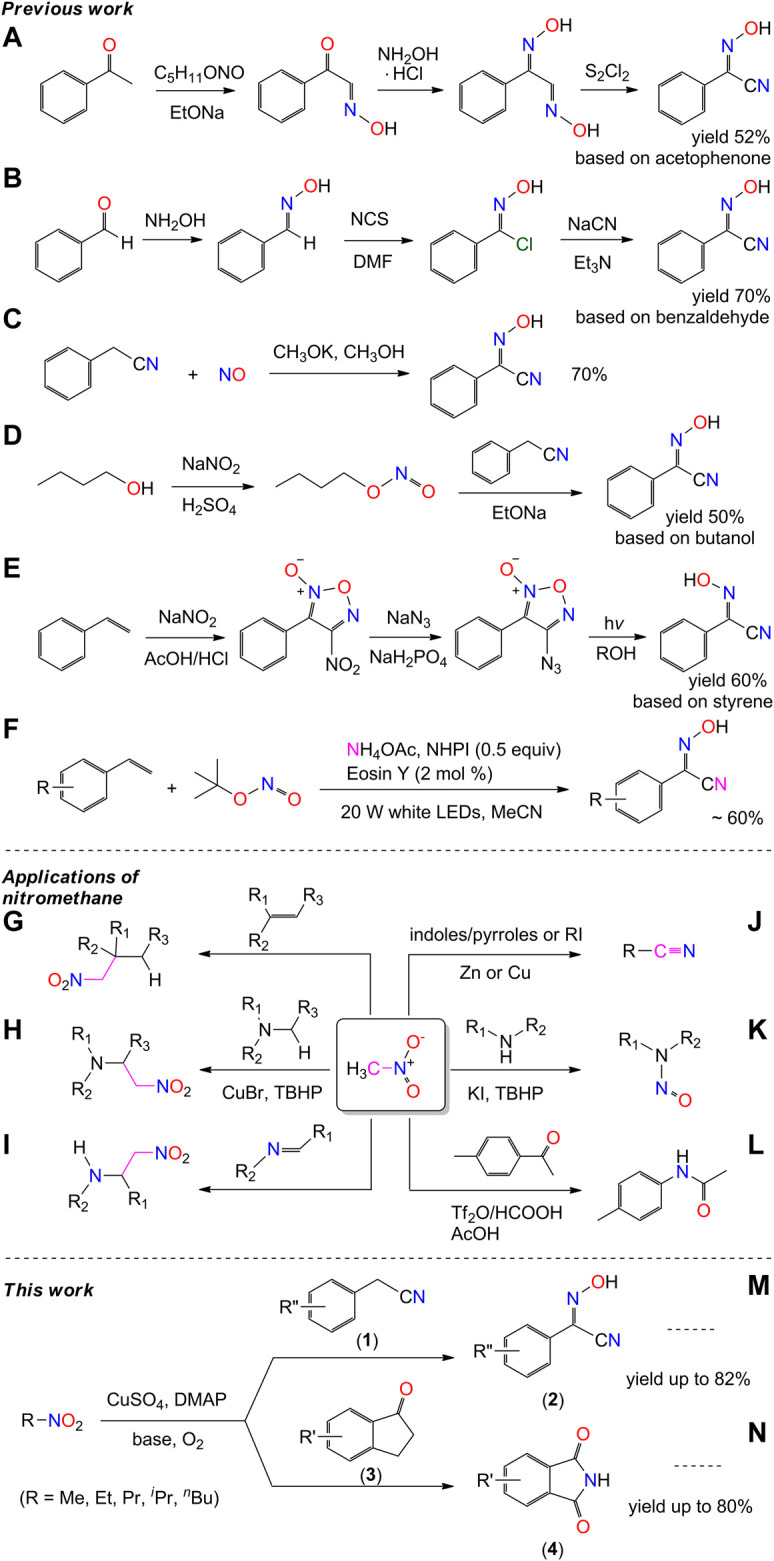
Previous work of the synthesis of 2-(hydroxyimino)-2-phenylacetonitrile and the reported applications of nitromethane as nitrogen donor.

Nitrogen is one of the most important elements that constitute organisms and plays an important role in life activities of biomolecules. Cyano-oxime compounds and their derivatives have shown tremendous potentials for the development of a variety of biological compounds such as herbicides, drugs, and antibacterial agents (Gerasimchuk et al., 2008; El-Faham et al., 2014). The presence of two important functional groups in a single molecule makes them widely applicable for the synthesis of nitrogenous organic compounds in chemical engineering (Robertson et al., 2005; Aakeröy et al., 2012; Soliman et al., 2017). Of these, 2-hydroxyimino-2-phenylacetonitriles (2) are useful building blocks for the construction of polynitrogen organic substances, especially for the synthesis of peptides and/or nitrogen-containing heterocyclic compounds (Itoh et al., 1975; Kunai et al., 1990; Neel and Zhao, 2018; Alam et al., 2020). A few methods are available for the synthesis of these compounds, including the condensation of phenylketone/benzaldehyde and the cyanidation of their secondary products (Schemes 1A,B) (Ma et al., 1999; Uysal, 2010; Lemercier and Pierce, 2014; Kryshenko et al., 2016; Ushakov et al., 2019), the nitrosylation and nitridation of benzyl-cyanide (Schemes 1C,D) (Dubery et al., 1999; Bohle and Perepichka, 2009; Bohle et al., 2013; Neel and Zhao, 2018), and the nitration and nitrosation of styrene (Schemes 1E,F) (Kunai et al., 1990; Alam et al., 2020). However, these methods suffer from several drawbacks, such as the use of toxic reagents, rigorous reaction conditions, the cost of starting materials, multistep processes, and/or low yields. Thus, a simple and efficient method for the synthesis of compound 2 is desired, consistent with the development of benzyl-cyano-oxime compounds and their related pharmacological and/or material chemistry.

In the literature, the direct protocol to enhance the scope of conventional synthesis of N-containing compounds generally involves the development and utilization of nitrogen transfer agents. Many nitrogen donors have been used in the catalytic transformations, *e*.*g*., oxidative amination, nitrogenation, and nitrogen heterocyclization. They include diazo/azide (Hennessy and Betley, 2013), isocyanide (Kim and Hong, 2015), cyanide (Wu et al., 2016a), ammonia (Shimokawa et al., 2016), sulfonamide/amide (Wang et al., 2019a), and hydroxylamine (Mudshinge et al., 2020). In contrast, the reactivity of nitromethane as a nitrogen donor was rarely developed, until a recent article in the journal of *Science* provided a reference of nitromethane activation for use in the Schmidt reaction (Scheme 1L) (Liu et al., 2019). The efficient utilization of nitro compounds as nitrogen donors has attracted considerable attention, and the interest in the activation of nitromethane grows.

Nitromethane is shelf-stable and commercially available. It is commonly used as a solvent for organic synthesis. It is also used as an intermediate in the carbon-chain-growth synthesis reaction owing to the strongly electrophilic properties of the nitro group (Xu et al., 2015; Aksenov et al., 2015; Wu et al., 2016b; Aksenov et al., 2020), for example, nitroaldol reactions (Jacobsen, 2001; Akagawa and Kudo, 2012; Kwiatkowski et al., 2014; Fu et al., 2019), cross dehydrogenative coupling reaction (Li et al., 2006; Basle and Li, 2007), and nitro-Mannich reactions (Schemes 1G–I) (Noble and Anderson, 2013). These reactions took place in the active site of the methyl group, without any change to the skeleton of the nitro group. Moreover, nitromethane is also used as cyano and nitroso sources in the formation of nitriles and nitrosamines (Schemes 1J,K), in the presence of zinc or copper catalysts, respectively (Nagase et al., 2014; Sakai et al., 2015; Chaudhary et al., 2016; Ogiwara et al., 2017; Qiu et al., 2018; Mudithanapelli et al., 2019). However, the substrates of those reactions are limited to heterocycles/aryl iodides and amines.

Copper complexes are often used as oxidants and/or oxidation catalysts in organic catalytic synthesis. As advanced oxidizing agents, copper-oxygen species have been used in the activation of C-H bond of alkyl group (Allen et al., 2013; Thorseth et al., 2013; Solomon et al., 2014; Elwell et al., 2017). On considering our previous work on the C-H bond activation of pyridine (Xiang et al., 2019), we set out to study the application of copper(II)-oxygen species on the activation of nitromethane, from the perspective of applied chemistry and practical use. We herein report a new method for the synthesis of 2-hydroxyimino-2-phenylacetonitrile (2a) and its derivatives, by nitrosation of phenylacetonitriles with nitromethane under dioxygen atmosphere (Scheme 1M). The employment of easily available reagents, environmentally friendly solvents, and mild reaction conditions make this process very practical. Meanwhile, the copper(II)-oxygen facilitated nitrosation reaction is validated with the amination of indanones with nitromethane in yields up to 80% (Scheme 1N). Experimental studies showed that the methyl nitrite could be an intermediate of the activation of nitromethane in our system, through which the final products two and four were obtained by means of nucleophilic reaction and electrophilic reaction, respectively.

## Material and Methods

### General Information

The single-crystal data of compounds were collected by a Cu/Mo-Kα rotating anode source, using a Supernova diffractometer with the ω-scan method. ESI-MS were obtained using a Bruker Impact II quadrupole time-of-flight mass spectrometer. ^1^H NMR and ^13^C NMR spectra were recorded on Bruker Avance III (400 MHz) and JNM ECS400S (400 MHz). Chemical shifts are expressed in δ ppm values with reference to tetramethylsilane (TMS) as an internal standard. NMR multiplicities are abbreviated as follows: s = singlet, d = doublet, and m = multiplet. Coupling constants (J) are expressed in Hz. ^1^H and ^13^C NMR spectra are provided for all the compounds in the SI. Pyridine was distilled before use. MeNO_2_ and DMF were distilled over CaH_2_ and stored over molecular sieve (3Å). THF was distilled with metal sodium over a period of 5 h. All other reagents were of analytical grade, purchased from commercial sources, and used as received.

*Warning*. The starting materials of nitroalkanes and the nitrointermediates generated during reactions are energetic and should be handled as if they are explosive materials. All the reactions should be conducted behind a blast shield.

#### General Procedure for the Synthesis of Compounds Shown in Scheme 2

*For safety reasons, the feeding order of chemicals should be followed*. To a mixture of phenylacetonitriles (0.60 mmol), 4-dimethylaminopyridine (DMAP, 0.40 mmol), NaOH (0.40 mmol), CuSO_4_.5H_2_O (0.20 mmol), and THF (1 mL) in a 50 mL Teflon screw-cap sealed tube, MeNO_2_ (0.20 mmol) was added slowly in air atmosphere. The tube was charged with O_2_ (1 atm) and the mixture was stirred at 120 °C for 24 h. After cooling to room temperature, the reaction mixture was diluted with dichloromethane (20 mL), filtered through a pad of silica gel, and concentrated under reduced pressure. The crude product was purified on a silica gel column eluted with petroleum ether/ethyl acetate (7:1 v/v) to afford the products in yields up to 82% (2e). The scale-up production of compound 2 was examined with the typical nitrile of phenylacetonitrile. The reaction of PhCH_2_CN (3.51 g, 30 mmol), DMAP (2.44 g, 20 mmol), NaOH (0.80 g, 20 mmol), CuSO_4_.5H_2_O (2.50 g, 10 mmol), THF (30 mL), and MeNO_2_ (0.61 g, 10 mmol) under the same conditions afforded the product 2a in yield 68% (0.99 g).

**SCHEME 2:**
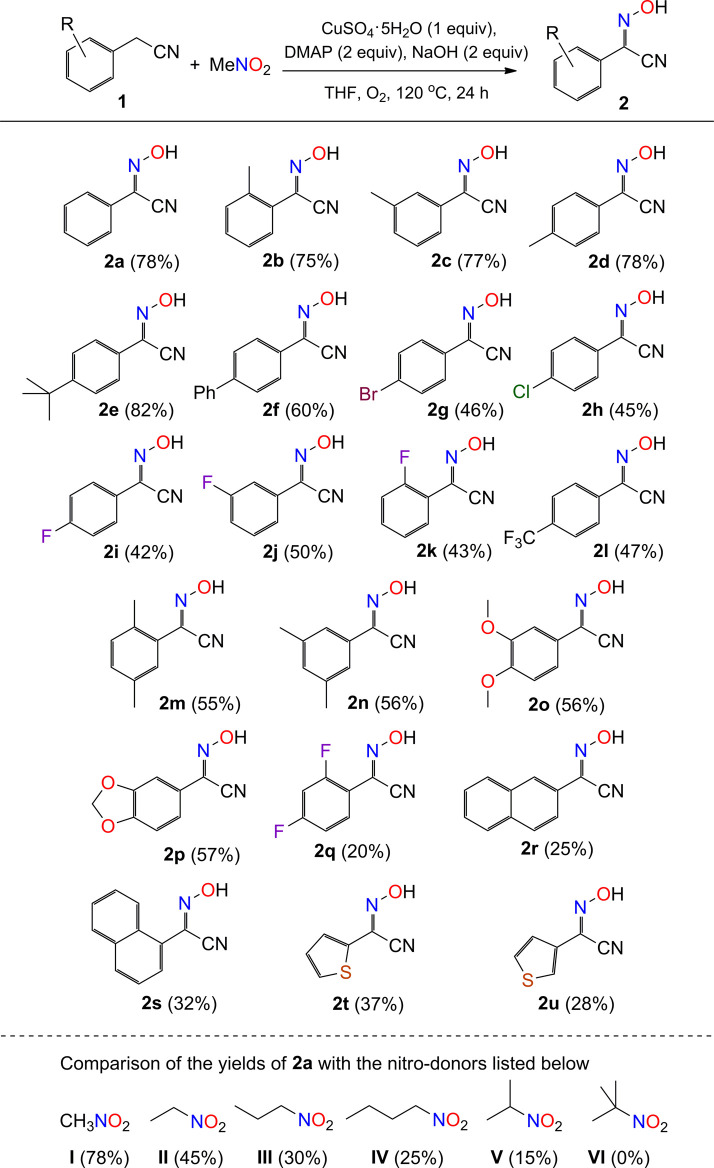
Scope of phenylacetonitriles with respect to compound 2^a^. ^*a*^Reaction conditions: substrate 1 (0.6 mmol), MeNO_2_ (0.2 mmol), CuSO_4_.5H_2_O (0.2 mmol), DMAP (0.4 mmol), NaOH (0.4 mmol), THF (1 mL), O_2_ (1 atm), 120 °C, and 24 h.

#### General Procedure for the Synthesis of Compounds Shown in Scheme 3

*Part a. For safety reasons, the reaction procedure should be strictly followed*. A mixture of indanones (0.20 mmol), DMAP (0.40 mmol), ^*t*^BuOK (0.40 mmol), and CuSO_4_.5H_2_O (0.20 mmol) in a 50 mL Teflon screw-cap sealed tube was stirred for 5 min in air atmosphere. The mixture was cooled to 0 °C and MeNO_2_ (1 mL) was added dropwise while stirring. After the addition was completed, the mixture was warmed up, charged with O_2_ (1 atm), and stirred at 120 °C for 24 h. After cooling to room temperature, the reaction mixture was diluted with dichloromethane (20 mL), filtered through a pad of silica gel, and concentrated under reduced pressure. The crude product was purified on a silica gel column eluted with dichloromethane/methanol (20:1 v/v) to afford the products in yields up to 80% (4e). The scale-up production of compound 4 was examined with the typical starting material of 1-indanone. The reaction of 1-indanone (1.32 g, 10 mmol), DMAP (2.44 g, 20 mmol), ^*t*^BuOK (2.24 g, 20 mmol), CuSO_4_.5H_2_O (2.50 g, 10 mmol), and MeNO_2_ (30 mL) under the same conditions shown above afforded the product 4a in yield 65% (0.96 g).

**SCHEME 3:**
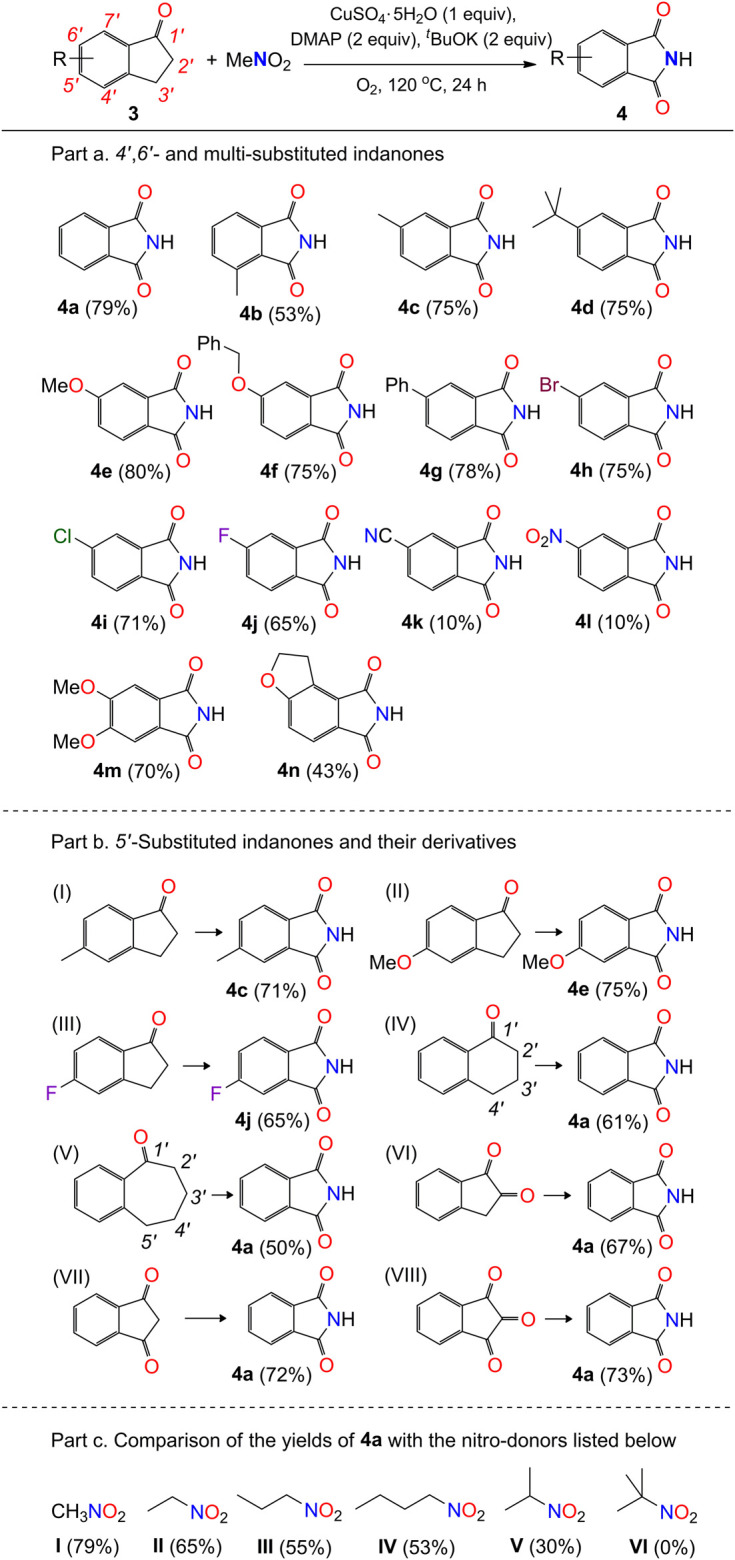
Scope of indanones with respect to compound 4^a^. ^*a*^Reaction conditions: substrate 3 (0.2 mmol), MeNO_2_ (1 mL), CuSO_4_.5H_2_O (0.2 mmol), DMAP (0.4 mmol), ^*t*^BuOK (0.4 mmol), O_2_ (1 atm), 120 °C, and 24 h.

*Part b.* The above procedure (part a) was modified for the preparation of compounds listed in Part b, with the use of corresponding 1-carbonyl-benzohetercyclic compounds (0.2 mmol) as substrates.

*Part c*. The above procedure (part a) was used for the preparation of compound 4a with the use of nitroethane, nitropropane, 1-nitrobutane, 2-nitropropane, and 2-methyl-2-nitropropane as solvents (1 mL), respectively.

#### Experimental Procedure for the Synthesis of Compounds Shown in Scheme 4

**SCHEME 4:**
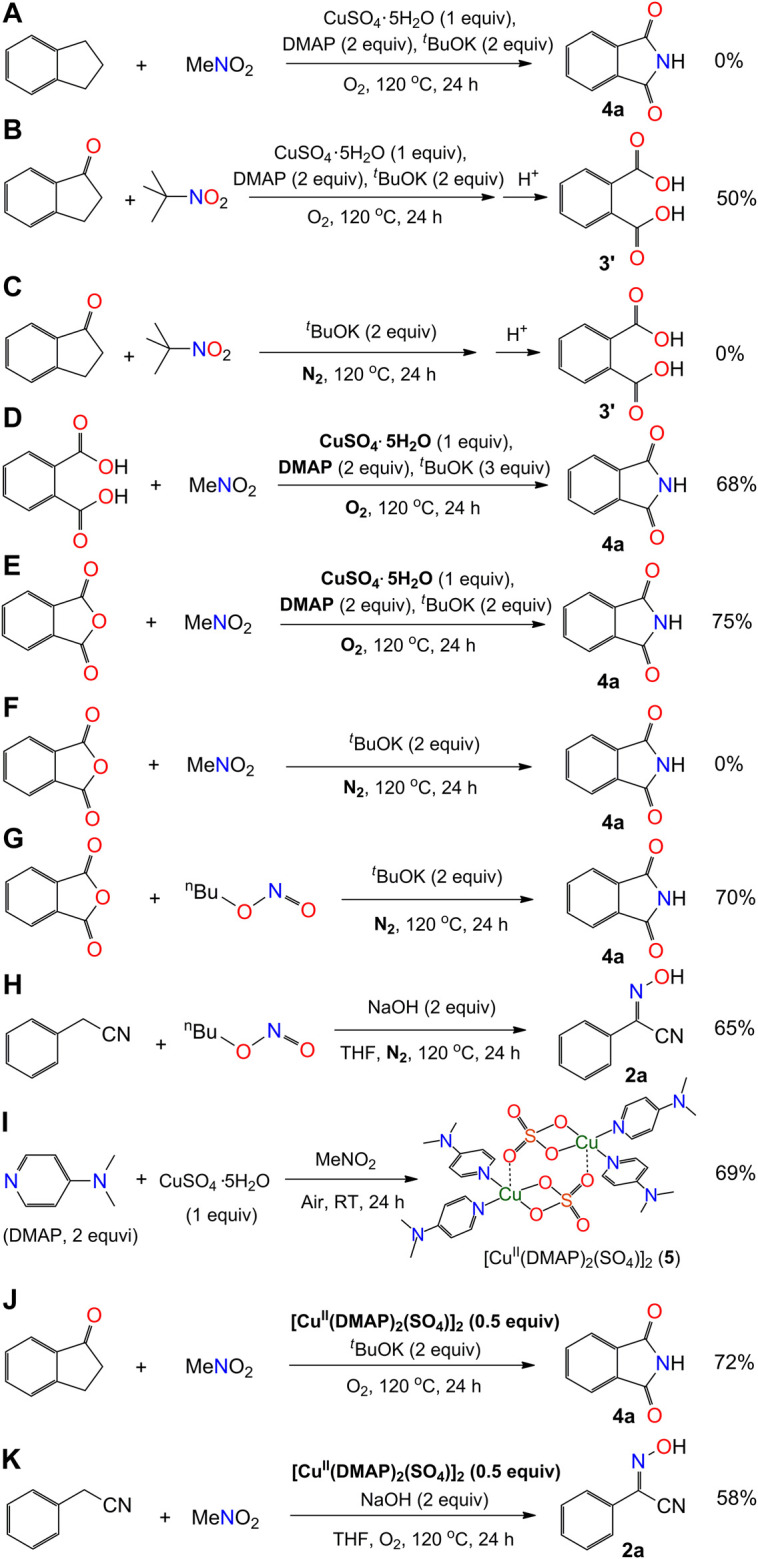
Mechanistic exploration of the synthesis of phthalimide and 2-hydroxyimino-2-phenylacetonitrile.

*For Safety Reasons, the Feeding Order of Chemicals Should Be Followed*

- A mixture of indanone (23.6 mg, 0.20 mmol), DMAP (48.8 mg, 0.40 mmol), ^*t*^BuOK (44.8 mg, 0.40 mmol), and CuSO_4_.5H_2_O (50.0 mg, 0.20 mmol) in a 50 mL Teflon screw-cap sealed tube was stirred for 5 min in air atmosphere. The mixture was cooled to 0 °C and MeNO_2_ (1 mL) was added dropwise while stirring. After the addition was completed, the mixture was warmed up to room temperature. The tube was charged with O_2_ (1 atm) and the mixture was stirred at 120 °C for 24 h. After cooling to room temperature, the reaction mixture was diluted with dichloromethane (20 mL), filtered through a pad of silica gel, and concentrated under reduced pressure. The residue was checked with the techniques of Thin-Layer Chromatography (TLC) and ^1^H NMR, but no signal of compound 4a was found.
- A mixture of 1-indanone (26.4 mg, 0.20 mmol), DMAP (48.8 mg, 0.40 mmol), ^*t*^BuOK (44.8 mg, 0.40 mmol), and CuSO_4_.5H_2_O (50.0 mg, 0.20 mmol) in a 50 mL Teflon screw-cap sealed tube was stirred for 5 min in air atmosphere. The mixture was cooled to 0 °C and ^*t*^BuNO_2_ (1 mL) was added dropwise while stirring. After the addition was completed, the mixture was warmed up to room temperature. The tube was charged with O_2_ (1 atm) and the mixture was stirred at 120 °C for 24 h. After cooling to room temperature, the reaction mixture was diluted with dichloromethane (20 mL), acidified by acetic acid to pH = 3, filtered through a pad of silica gel, and concentrated under reduced pressure. The crude product was purified on a silica gel column eluted with dichloromethane/methanol (10:1 v/v) to afford the product 3′ in yield 50% (16.6 mg).
- A mixture of 1-indanone (26.4 mg, 0.20 mmol) and ^*t*^BuOK (44.8 mg, 0.40 mmol) in a 50 mL Teflon screw-cap sealed tube was stirred for 5 min in air atmosphere. The mixture was cooled to 0 °C and ^*t*^BuNO_2_ (1 mL) was added dropwise while stirring. After the addition was completed, the mixture was warmed up to room temperature. The tube was charged with N_2_ (1 atm) and the mixture was stirred at 120 °C for 24 h. The reaction mixture was cooled, diluted with dichloromethane (20 mL), acidified by acetic acid to pH = 3, filtered through a pad of silica gel, and concentrated under reduced pressure. The residue was checked with the techniques of TLC and ^1^H NMR, but no signal of compound 3′ was found.
- A mixture of *o*-phthalic acid (33.2 mg, 0.20 mmol), DMAP (48.8 mg, 0.40 mmol), ^*t*^BuOK (44.8 mg, 0.40 mmol), and CuSO_4_.5H_2_O (50.0 mg, 0.20 mmol) in a 50 mL Teflon screw-cap sealed tube was stirred for 5 min in air atmosphere. The mixture was cooled to 0 °C and MeNO_2_ (1 mL) was added dropwise while stirring. After the addition was completed, the mixture was warmed up to room temperature. The tube was charged with O_2_ (1 atm) and stirred at 120 °C for 24 h. The reaction mixture was cooled, diluted with dichloromethane (20 mL), filtered through a pad of silica gel, and concentrated under reduced pressure. The crude product was purified on a silica gel column eluted with dichloromethane/methanol (20:1 v/v) to afford the product 4a in yield 68% (20.0 mg).
- A mixture of *o*-phthalic anhydride (29.6 mg, 0.20 mmol), DMAP (48.8 mg, 0.40 mmol), ^*t*^BuOK (44.8 mg, 0.40 mmol), and CuSO_4_.5H_2_O (50.0 mg, 0.20 mmol) in a 50 mL Teflon screw-cap sealed tube was stirred for 5 min in air atmosphere. The mixture was cooled to 0 °C and MeNO_2_ (1 mL) was added dropwise while stirring. The same procedure of (d) was followed for the processing of solution and the purification of crude product. The yield of phthalimide (4a) is 75% (22.0 mg).
- A mixture of *o*-phthalic anhydride (29.6 mg, 0.20 mmol) and ^*t*^BuOK (44.8 mg, 0.40 mmol) in a 50 mL Teflon screw-cap sealed tube was stirred for 5 min in air atmosphere. The mixture was cooled to 0 °C and ^*t*^BuNO_2_ (1 mL) was added dropwise while stirring. After the addition was completed, the mixture was warmed up to room temperature. The tube was charged with N_2_ (1 atm) and the mixture was stirred at 120 °C for 24 h. The reaction mixture was cooled, diluted with dichloromethane (20 mL), filtered through a pad of silica gel, and concentrated under reduced pressure. The residue was checked with the techniques of TLC and ^1^H NMR, but no signal of compound 4a was found.
- A mixture of *o*-phthalic anhydride (29.6 mg, 0.20 mmol) and ^*t*^BuOK (44.8 mg, 0.40 mmol) in a 50 mL Teflon screw-cap sealed tube was stirred for 5 min in air atmosphere. The mixture was cooled to 0 °C and ^*n*^BuONO (1 mL) was added dropwise while stirring. After the addition was completed, the mixture was warmed up to room temperature. The tube was charged with N_2_ (1 atm) and stirred at 120 °C for 24 h. The reaction mixture was cooled, diluted with dichloromethane (20 mL), filtered through a pad of silica gel, and concentrated under reduced pressure. The crude product was purified on a silica gel column eluted with dichloromethane/methanol (20:1 v/v) to afford the product 4a in yield 70% (20.6 mg).
- To a mixture of phenylacetonitrile (70.3 mg, 0.60 mmol), NaOH (16.0 mg, 0.40 mmol), and THF (1 mL) in a 50 mL Teflon screw-cap sealed tube, ^*n*^BuONO (20.6 mg, 0.20 mmol) was added slowly in air atmosphere. The tube was charged with N_2_ (1 atm) and the mixture was stirred at 120 °C for 24 h. After cooling to room temperature, the reaction mixture was diluted with dichloromethane (20 mL), filtered through a pad of silica gel, and concentrated under reduced pressure. The crude product was purified on a silica gel column eluted with petroleum ether/ethyl acetate (7:1 v/v) to afford 2-(hydroxyimino)-2-phenylacetonitrile (2a) in yield 65% (19.0 mg).
- A mixture of 1-indanone (26.4 mg, 0.20 mmol), ^*t*^BuOK (44.8 mg, 0.40 mmol), and compound 5 (80.8 mg, 0.10 mmol, green crystalline solid) in a 50 mL Teflon screw-cap sealed tube was stirred for 5 min in air atmosphere. The mixture was cooled to 0 °C and MeNO_2_ (1 mL) was added dropwise while stirring. The same procedure of (d) was followed for the processing of solution and the purification of crude product. The yield of phthalimide (4a) is 72% (21.2 mg).
- To a mixture of phenylacetonitrile (70.3 mg, 0.60 mmol), NaOH (16.0 mg, 0.40 mmol), compound 5 (80.8 mg, 0.10 mmol, green crystalline solid), and THF (1 mL) in a 50 mL Teflon screw-cap sealed tube, MeNO_2_ (12.2 mg, 0.20 mmol) was added slowly in air atmosphere. The tube was charged with O_2_ (1 atm) and the mixture was stirred at 120 °C for 24 h. After cooling to room temperature, the reaction mixture was diluted with dichloromethane (20 mL), filtered through a pad of silica gel, and concentrated under reduced pressure. The crude product was purified on a silica gel column eluted with petroleum ether/ethyl acetate (7:1 v/v) to afford the product 2a in yield 58% (17.0 mg).

#### The Synthetic Procedure for Compound 5

A mixture of CuSO_4_.5H_2_O (25.0 mg, 0.10 mmol) and DMAP (24.4 mg, 0.20 mmol) was stirring in dried MeNO_2_ (10 mL) for 30 min to give a dark green solution. The mixture was filtered and the filtrate was added Et_2_O to deposit the product as some green crystalline solid, which was collected, washed with THF/Et_2_O (0.5/1 mL), and dried (28 mg, 69%). Anal. Calcd. for C_28_H_40_Cu_2_N_8_O_8_S_2_: C, 41.63; H, 4.99; N, 13.87. Found (%): C, 41.16; H, 4.94; N, 14.03. The crystal suitable for X-ray crystallography was grown by diffusion of Et_2_O into a solution of compound 5 in DMF.

## NMR Data

### 2-(Hydroxyimino)-2-phenylacetonitrile (2a) (Alam et al., 2020)

Yield, 78% (22.8 mg); white powder; melting point, 120–122 °C (literature 120–122 °C); ^1^H NMR (400 MHz, DMSO-d_6_): δ 13.89 (s, 1H), 7.81 (brs, 2H), 7.62 (brs, 3H); ^13^C NMR (100 MHz, DMSO-d_6_): δ 131.7, 131.4, 130.0, 129.8, 126.0, 110.6.

### 2-(Hydroxyimino)-2-(2-methyl)-phenylacetonitrile (2b) (Alam et al., 2020)

Yield, 75% (24.1 mg); white powder; melting point, 132–134 °C; ^1^H NMR (400 MHz, DMSO-d_6_): δ 13.85 (s, 1H), 7.57 (d, *J* = 7.5 Hz, 1H), 7.49 (t, *J* = 7.1 Hz, 1H), 7.41–7.45 (m, 2H), 2.51 (s, 3H); ^13^C NMR (100 MHz, DMSO-d_6_): δ 137.0, 132.1, 131.8, 130.9, 129.7, 129.3, 127.2, 111.4, 21.1; HRMS m/z (ESI) [M + Na^+^] calculated for C_9_H_8_N_2_O_1_Na, 183.0534; found, 183.0559.

### 2-(Hydroxyimino)-2-(3-methyl)-phenylacetonitrile (2c) (Alam et al., 2020)

Yield, 77% (24.7 mg); white powder; melting point, 124–126 °C; ^1^H NMR (400 MHz, DMSO-d_6_): δ 13.75 (s, 1H), 7.53 (s, 1H), 7.52 (d, *J* = 7.6 Hz, 1H), 7.41 (t, *J* = 7.6 Hz, 1H), 7.35 (d, *J* = 7.6 Hz, 1H), 2.37 (s, 3H); ^13^C NMR (100 MHz, DMSO-d_6_): δ 138.8, 131.6, 131.2, 129.5, 129.3, 125.9, 122.8, 110.2, 20.9.

### 2-(Hydroxyimino)-2-(4-methyl)-phenylacetonitrile (2d) (Alam et al., 2020)

Yield, 78% (25.0 mg); white powder; melting point, 150–152 (literature 150–152 °C); ^1^H NMR (400 MHz, DMSO-d_6_): δ 13.70 (s, 1H), 7.66 (d, *J* = 8.0 Hz, 2H), 7.38 (d, *J* = 8.0 Hz, 2H), 2.40 (s, 3H); ^13^C NMR (100 MHz, DMSO-d_6_): δ 141.4, 131.6, 130.4, 127.3, 126.0, 110.7, 21.4.

### 4-(tert-Butyl)-N-hydroxybenzimidoyl Cyanide (2e) (Alam et al., 2020)

Yield, 82% (33.1 mg); pale yellow powder; melting point, 158–160 °C (literature 158–160 °C); ^1^H NMR (400 MHz, DMSO-d_6_): δ 13.58 (s, 1H), 7.53 (d, *J* = 8.0 Hz, 2H), 7.41 (d, *J* = 8.0 Hz, 2H), 1.16 (s, 9H); ^13^C NMR (100 MHz, DMSO-d_6_): δ 153.8, 131.1, 126.9, 126.2, 125.4, 110.2, 34.7, 30.9.

### 2-(Hydroxyimino)-2-(4-phenyl)-phenylacetonitrile (2f) (Alam et al., 2020)

Yield, 60% (26.4 mg); white powder; melting point, 140–142 °C (literature 141–143 °C); ^1^H NMR (400 MHz, DMSO-d_6_): δ 13.83 (s, 1H), 7.85 (d, *J* = 8.0 Hz, 2H), 7.81 (d, *J* = 8.0 Hz, 2H), 7.73 (d, *J* = 7.2 Hz, 2H), 7.50 (t, *J* = 7.2 Hz, 2H), 7.42 (t, *J* = 7.2 Hz, 1H); ^13^C NMR (100 MHz, DMSO-d_6_): δ 142.4, 138.9, 130.9, 129.1, 128.6, 128.2, 127.5, 126.8, 126.1, 110.2; HRMS m/z (ESI) [M + H^+^]: calculated for C_14_H_11_N_2_O_1_, 223.0872; found, 223.0866.

### 2-(Hydroxyimino)-2-(4-bromo)-phenylacetonitrile (2g) (Gao et al., 2018)

Yield, 46% (20.7 mg); white powder; melting point, 127–129 °C (literature 123–125 °C); ^1^H NMR (400 MHz, DMSO-d_6_): δ 13.94 (s, 1H), 7.74 (d, *J* = 8.0 Hz, 2H), 7.66 (d, *J* = 8.0 Hz, 2H); ^13^C NMR (100 MHz, DMSO-d_6_): δ 132.3, 130.4, 128.8, 127.4, 124.3, 109.9; HRMS m/z (ESI) [M + 2Na^+^ - H^+^]: calculated for C_8_H_4_N_2_O_1_BrNa_2_, 268.9297; found, 268.9295.

### 2-(Hydroxyimino)-2-(4-chloro)-phenylacetonitrile (2h) (Neel and Zhao, 2018)

Yield, 45% (16.3 mg); white powder; melting point, 137–139 °C (literature 139–141 °C); ^1^H NMR (400 MHz, DMSO-d_6_): δ 13.95 (s, 1H), 7.74 (d, *J* = 8.6 Hz, 2H), 7.60 (d, *J* = 8.6 Hz, 2H); ^13^C NMR (100 MHz, DMSO-d_6_): δ 135.5, 130.3, 129.4, 128.5, 127.3, 109.9.

### 2-(Hydroxyimino)-2-(4-fluoro)-phenylacetonitrile (2i) (Gao et al., 2018)

Yield, 42% (13.8 mg); white powder; melting point, 112–114 °C (literature 112–114 °C); ^1^H NMR (400 MHz, DMSO-d_6_): δ 13.83 (s, 1H), 7.78 (brs, 2H), 7.37 (dd, *J*_HF_ = 8.0 Hz, *J*_HH_ = 8.0 Hz, 2H); ^13^C NMR (100 MHz, DMSO-d_6_): δ 164.0 (d, *J* = 225.3 Hz), 130.2, 128.0 (d, *J* = 9.0 Hz), 116.5 (d, *J* = 22.5 Hz), 110.1, 99.6; HRMS m/z (ESI) [M + 2Na^+^ - H^+^]: calculated for C_8_H_4_N_2_O_1_F_1_Na_2_, 209.0098; found, 209.0101.

### 2-(Hydroxyimino)-2-(3-fluoro)-phenylacetonitrile (2j) (Alam et al., 2020)

Yield, 50% (16.4 mg); white powder; melting point, 120–122 °C (literature 120–122 °C); ^1^H NMR (400 MHz, DMSO-d_6_): δ 14.03 (s, 1H), 7.53–7.61 (m, 2H), 7.49 (d, *J* = 9.6 Hz, 1H), 7.39 (td, *J*_HH_ = 8.0 Hz, *J*_HF_ = 3.0 Hz, 1H); ^13^C NMR (100 MHz, DMSO-d_6_): δ 162.7 (d, *J* = 243.6 Hz), 132.2 (d, *J* = 8.4 Hz), 132.1 (d, *J* = 8.4 Hz), 130.7 (d, *J* = 3.6 Hz), 122.6 (d, *J* = 2.8 Hz), 118.3 (d, *J* = 21.3 Hz), 112.3 (d, *J* = 24.2 Hz), 110.3.

### 2-(Hydroxyimino)-2-(2-fluoro)-phenylacetonitrile (2k) (Alam et al., 2020)

Yield, 43% (14.1 mg); white powder; melting point, 128–130 °C (literature 132–134 °C); ^1^H NMR (400 MHz, DMSO-d_6_): δ 14.12 (s, 1H), 7.69 (dd, *J*_HH_ = 7.6 Hz, *J*_HF_ = 6.4 Hz, 1H), 7.59 (td, *J*_HH_ = 8.0 Hz, *J*_HF_ = 5.2 Hz, 1H), 7.34–7.43 (m, 2H); ^13^C NMR (100 MHz, DMSO-d_6_): δ 159.5 (d, *J* = 250.7 Hz), 133.4 (d, *J* = 8.6 Hz), 129.5, 127.1, 125.8 (d, *J* = 3.5 Hz), 118.2 (d, *J* = 10.4 Hz), 117.2 (d, *J* = 20.8 Hz), 110.5.

### 2-(Hydroxyimino)-2-(4-trifluoromethyl)-phenylacetonitrile (2l) (Neel and Zhao, 2018)

Yield, 47% (20.1 mg); white powder; melting point, > 200 °C; ^1^H NMR (400 MHz, DMSO-d_6_): δ 14.21 (s, 1H), 7.88–7.95 (m, 4H); ^13^C NMR (100 MHz, DMSO-d_6_): δ 133.5, 130.6 (q, *J* = 32.0 Hz), 130.3, 126.4, 126.3 (q, *J* = 3.7 Hz), 123.9 (q, *J* = 270.8 Hz), 109.9.

### 2-(Hydroxyimino)-2-(2,5-dimethyl)-phenylacetonitrile (2m)

Yield, 55% (19.2 mg); white powder; melting point, > 200 °C; ^1^H NMR (400 MHz, DMSO-d_6_): δ 13.75 (s, 1H), 7.30 (s, 1H), 7.24 (brs, 2H), 2.38 (s, 3H), 2.32 (s, 3H); ^13^C NMR (100 MHz, DMSO-d_6_): δ 136.2, 133.7, 132.0, 131.7, 131.4, 129.9, 128.9, 111.3, 20.8, 20.6.

### 2-(Hydroxyimino)-2-(3,5-dimethyl)-phenylacetonitrile (2n)

Yield, 56% (19.5 mg); white powder; melting point, 153–155 °C; ^1^H NMR (400 MHz, DMSO-d_6_): δ 13.70 (s, 1H), 7.32 (s, 2H), 7.16 (s, 1H), 2.32 (s, 6H); ^13^C NMR (100 MHz, DMSO-d_6_): δ 139.1, 132.8, 131.7, 129.9, 123.6, 110.7, 21.2; HRMS m/z (ESI) [M + 2Na^+^ - H^+^]: calculated for C_10_H_9_N_2_O_1_Na_2_, 219.0505; found, 219.0504.

### 2-(Hydroxyimino)-2-(3,4-dimethoxyl)-phenylacetonitrile (2o)

Yield, 56% (23.1 mg); white powder; melting point, 183–185 °C (literature 183–191 °C); ^1^H NMR (400 MHz, DMSO-d_6_): δ 13.48 (s, 1H), 7.25 (s, 1H), 7.23 (d, *J* = 8.0 Hz, 1H), 7.10 (d, *J* = 8.0 Hz, 1H), 3.82 (s, 3H), 3.81 (s, 3H); ^13^C NMR (100 MHz, DMSO-d_6_): δ 151.7, 149.6, 131.3, 122.5, 120.1, 112.2, 110.7, 107.6, 56.1, 55.9; HRMS m/z (ESI) [M + 2Na^+^ - H^+^]: calculated for C_10_H_9_N_2_O_3_Na_2_, 251.0403; found, 251.0403.

### N-Hydroxybenzo[d][1,3]dioxole-5-carbimidoyl Cyanide (2p)

Yield, 57% (21.7 mg); white powder; melting point, 76–78 °C (literature 76–78 °C); ^1^H NMR (400 MHz, DMSO-d_6_): δ 13.55 (s, 1H), 7.23 (s, 1H), 7.19 (d, *J* = 8.1 Hz, 1H), 7.06 (d, *J* = 8.1 Hz, 1H), 6.12 (s, 2H); ^13^C NMR (100 MHz, DMSO-d_6_): δ 150.2, 148.8, 131.2, 124.1, 121.7, 110.6, 109.2, 104.8, 102.5; HRMS m/z (ESI) [M + 2Na^+^ - H^+^]: calculated for C_9_H_5_N_2_O_3_Na_2_, 235.0090; found, 235.0089.

### 2,4-Difluoro-N-hydroxybenzimidoyl Cyanide (2q)

Yield, 20% (14.6 mg) for 0.4 mmol; pale yellow powder; melting point, > 200 °C; ^1^H NMR (400 MHz, DMSO-d_6_): δ 14.14 (s, 1H), 7.72–7.80 (m, 1H), 7.49 (t, *J* = 8.8 Hz, 1H), 7.26 (t, *J* = 8.8 Hz, 1H); ^13^C NMR (100 MHz, DMSO-d_6_): δ 164.1 (dd, *J* = 262.3, 11.7 Hz), 160.0 (dd, *J* = 253.1, 12.8 Hz), 131.2 (dd, *J* = 10.3, 3.2 Hz), 126.4, 115.0 (dd, *J* = 10.6, 3.7 Hz), 113.3 (dd, *J* = 22.0, 3.6 Hz), 110.4, 105.8 (t, *J* = 26.0 Hz); HRMS m/z (ESI) [M + H^+^]: calculated for C_8_H_5_N_2_O_1_F_2_, 183.0370; found, 183.0342.

### 2-(Hydroxyimino)-2-naphthylacetonitrile (2r) (Gao et al., 2018)

Yield, 25% (9.8 mg); white powder; melting point, 143–145 °C (literature 145 °C); ^1^H NMR (400 MHz, DMSO-d_6_): δ 13.88 (s, 1H), 8.22 (s, 1H), 8.11 (d, *J* = 9.2 Hz, 1H), 8.02 (d, *J* = 8.8 Hz, 1H), 7.98 (d, *J* = 9.2 Hz, 1H), 7.89 (d, *J* = 8.8 Hz, 1H), 7.58–7.65 (m, 2H); ^13^C NMR (100 MHz, DMSO-d_6_): δ 134.2, 133.0, 131.9, 129.6, 129.2, 128.3, 127.73, 127.71, 127.0, 121.8, 110.6; HRMS m/z (ESI) [M + 2Na^+^ - H^+^]: calculated for C_12_H_7_N_2_O_1_Na_2_, 241.0348; found, 241.0350.

### Hydroxy-1-naphthimidoyl Cyanide (2s)

Yield, 32% (25.1 mg) for 0.4 mmol; pale yellow powder; melting point, > 200 °C; ^1^H NMR (400 MHz, DMSO-d_6_): δ 14.05 (s, 1H), 8.45 (d, *J* = 8.4 Hz, 1H), 8.10 (d, *J* = 8.4 Hz, 1H), 8.04 (d, *J* = 7.2 Hz, 1H), 7.82 (d, *J* = 7.2 Hz, 1H), 7.60–7.68 (m, 3H); ^13^C NMR (100 MHz, DMSO-d_6_): δ 134.0, 131.8, 131.5, 129.9, 129.4, 129.0, 128.3, 127.2, 126.7, 125.9, 125.0, 111.6; HRMS m/z (ESI) [M + Na^+^]: calculated for C_12_H_8_N_2_O_1_Na, 219.0534; found, 219.0514.

### N-Hydroxythiophene-2-carbimidoyl Cyanide (2t)

Yield, 37% (22.5 mg) for 0.4 mmol; white powder; melting point, 103–105 °C (literature 103–104 °C); ^1^H NMR (400 MHz, DMSO-d_6_): δ 14.22 (s, 1H), 7.97 (dd, *J* = 5.2, 1.2 Hz, 1H), 7.65 (dd, *J* = 3.6, 1.2 Hz, 1H), 7.25 (dd, *J* = 5.2, 4.0 Hz, 1H); ^13^C NMR (100 MHz, DMSO-d_6_): δ 134.1, 131.7, 129.7, 128.6, 127.3, 114.9; HRMS m/z (ESI) [M + H^+^]: calculated for C_6_H_5_N_2_O_1_S_1_, 153.0123; found, 153.0114.

### N-Hydroxythiophene-3-carbimidoyl Cyanide (2u)

Yield, 28% (17 mg) for 0.4 mmol; white powder; melting point, 98–100 °C (literature 96–106 °C); ^1^H NMR (400 MHz, DMSO-d_6_): δ 13.83 (s, 1H), 8.47 (dd, *J* = 3.2, 1.2 Hz), 7.73 (dd, *J* = 5.2, 3.2 Hz), 7.59 (dd, *J* = 5.2, 1.2 Hz); ^13^C NMR (100 MHz, DMSO-d_6_): δ 132.5, 129.7, 128.8, 128.08, 128.06, 116.1; HRMS m/z (ESI) [M + H^+^]: calculated for C_6_H_5_N_2_O_1_S_1_, 153.0123; found, 153.0114.

### Phthalimide (4a) (Wang et al., 2019b)

Yield, 79% (23.3 mg); white powder; melting point, > 200 °C (literature 232–234 °C); ^1^H NMR (400 MHz, DMSO-d_6_): δ 11.40 (s, 1H), 7.83 (s, 4H); ^13^C NMR (100 MHz, DMSO-d_6_): δ 169.7, 134.8, 133.1, 123.4.

### 3-Methyl-phthalimide (4b)

Yield, 53% (17.1 mg); white powder; melting point, 188–190 °C (literature 193–195 °C); ^1^H NMR (400 MHz, DMSO-d_6_): δ 11.22 (s, 1H), 7.66 (t, *J* = 7.4 Hz, 1H), 7.60 (d, *J* = 7.0 Hz, 1H), 7.57 (d, *J* = 7.6 Hz, 1H), 2.58 (s, 3H); ^13^C NMR (100 MHz, DMSO-d_6_): δ 170.6, 169.5, 137.6, 136.8, 134.3, 133.5, 129.6, 121.0, 17.4; HRMS m/z (ESI) [M + NH_4_^+^]: calculated for C_9_H_11_N_2_O_2_, 179.0820; found, 179.0815.

### 4-Methylphthalimide (4c) (Wang et al., 2019b)

Yield, 75% (24.1 mg) for 6-methyl-indanone, 57% (18.4 mg) for 5-methyl-indanone; white powder; melting point, 196–198 °C (literature 195–197 °C); ^1^H NMR (400 MHz, DMSO-d_6_): δ 11.22 (s, 1H), 7.69 (d, *J* = 7.6 Hz, 1H), 7.62 (s, 1H), 7.61 (d, *J* = 8.0 Hz, 1H), 2.46 (s, 3H); ^13^C NMR (100 MHz, DMSO-d_6_): δ 169.77, 169.68, 145.6, 135.2, 133.4, 130.5, 123.8, 123.3, 21.8.

### 4-tert-Butyl-phthalimide (4d)

Yield, 75% (30.5 mg); white powder; melting point, 134–136 °C (literature 132–134 °C); ^1^H NMR (400 MHz, DMSO-d_6_): δ 11.24 (s, 1H), 7.84 (d, *J* = 7.6 Hz, 1H), 7.78 (s, 1H), 7.73 (d, *J* = 7.6 Hz, 1H), 1.32 (s, 9H); ^13^C NMR (100 MHz, DMSO-d_6_): δ 169.4, 169.1, 158.0, 132.9, 131.3, 130.1, 122.8, 119.7, 35.4, 30.8; HRMS m/z (ESI) [M + 2Na^+^ - H^+^]: calculated for C_12_H_12_N_1_O_2_Na_2_, 248.0658; found, 248.0656.

### 4-Methoxylphthalimide (4e) (Wang et al., 2019b)

Yield, 80% (28.3 mg) for 6-methoxyl-indanone, 75% (26.6 mg) for 5-methoxyl-indanone; white powder; melting point, > 200 °C (literature 224–225 °C); ^1^H NMR (400 MHz, DMSO-d_6_): δ 11.17 (s, 1H), 7.72 (d, *J* = 8.0 Hz, 1H), 7.29 (s, 1H), 7.28 (d, *J* = 8.0 Hz, 1H), 3.90 (s, 3H); ^13^C NMR (100 MHz, DMSO-d_6_): δ 168.90, 168.85, 164.3, 135.3, 124.8, 124.5, 119.9, 107.9, 56.2.

### 4-Benzyloxy-phthalimide (4f) (Punchi Hewage et al., 2019)

Yield, 75% (38.0 mg); white powder; melting point, 154–156 °C (literature 159–161 °C); ^1^H NMR (400 MHz, DMSO-d_6_): δ 11.20 (s, 1H), 7.73 (d, *J* = 8.0 Hz, 1H), 7.47 (d, *J* = 8.0 Hz, 1H), 7.46 (s, 1H), 7.33–7.42 (m, 5H), 5.28 (s, 2H); ^13^C NMR (100 MHz, DMSO-d_6_): δ 168.88, 168.86, 163.3, 136.2, 135.2, 128.6, 128.2, 127.9, 124.80, 124.68, 120.7, 108.6, 70.1.

### 4-Phenylphthalimide (4g) (Wang et al., 2019b)

Yield, 78% (34.8 mg); white powder; melting point, 198–200 °C (literature 200–202 °C); ^1^H NMR (400 MHz, DMSO-d_6_): δ 11.38 (s, 1H), 8.10 (d, *J* = 7.6, 1H), 8.04 (s, 1H), 7.89 (d, *J* = 7.6 Hz, 1H), 7.80 (d, *J* = 7.6 Hz, 2H), 7.54 (t, *J* = 7.6 Hz, 2H), 7.47 (t, *J* = 7.6 Hz, 1H); ^13^C NMR (100 MHz, DMSO-d_6_): δ 169.01, 168.99, 146.2, 138.4, 133.7, 132.5, 131.3, 129.2, 128.8, 127.3, 123.6, 120.9.

### 4-Bromophthalimide (4h) (de Nanteuil et al., 2013)

Yield, 75% (35.8 mg); white powder; melting point, > 200 °C (literature 230–233 °C); ^1^H NMR (400 MHz, DMSO-d_6_): δ 11.48 (s, 1H), 8.02 (d, *J* = 7.8 Hz, H), 8.00 (s, 1H), 7.75 (d, *J* = 7.8 Hz, 1H); ^13^C NMR (100 MHz, DMSO-d_6_): δ 168.5, 167.9, 137.0, 134.7, 131.6, 127.9, 125.9, 124.9.

### 4-Chlorophthalimide (4i) (Wang et al., 2019b)

Yield, 71% (25.8 mg); white powder; melting point, > 200 °C (literature 228–230 °C); ^1^H NMR (400 MHz, DMSO-d_6_): δ 11.50 (s, 1H), 7.88 (s, 1H), 7.87 (d, *J* = 7.0 Hz, 1H), 7.82 (d, *J* = 7.0 Hz, 1H); ^13^C NMR (100 MHz, DMSO-d_6_): δ 168.4, 168.0, 139.1, 134.7, 134.1, 131.2, 124.8, 123.1.

### 4-Fluorophthalimide (4j)

Yield, 65% (21.5 mg) for 6-fluoro-indanone, 71% (23.4 mg) for 5-fluoro-indanone; white powder; melting point, 175–177 °C (literature 178–179 °C); ^1^H NMR (400 MHz, DMSO-d_6_): δ 11.46 (s, 1H), 7.89 (dd, *J*_HH_ = 8.0, *J*_HF_ = 4.4 Hz, 1H), 7.69 (dd, *J*_HF_ = 7.6, *J*_HH_ = 2.4 Hz, 1H), 7.64 (td, *J*_HF/HH_ = 8.8, *J*_HH_ = 2.4 Hz, 1H); ^13^C NMR (100 MHz, DMSO-d_6_): δ 168.3, 167.9 (d, *J* = 1.8 Hz), 165.8 (d, *J* = 251.3 Hz), 135.6 (d, *J* = 9.5 Hz), 128.8 (d, *J* = 2.4 Hz), 125.7 (d, *J* = 9.7 Hz), 121.2 (d, *J* = 23.7 Hz), 110.7 (d, *J* = 24.7 Hz); HRMS m/z (ESI) [M + 2Na^+^ - H^+^]: calculated for C_8_H_3_N_1_O_2_F_1_Na_2_, 209.9938; found, 209.9959.

### 4-Cyano-phthalimide (4k)

Yield, 10% (6.9 mg for 0.4 mmol); white powder; melting point, > 200 °C (literature 237–238 °C); ^1^H NMR (400 MHz, DMSO-d_6_): δ 11.75 (s, 1H), 8.35 (s, 1H), 8.29 (d, *J* = 7.6 Hz, 1H), 8.00 (d, *J* = 7.6 Hz, 1H); ^13^C NMR (100 MHz, DMSO-d_6_): δ 168.0, 167.8, 138.5, 136.1, 133.3, 126.8, 123.8, 117.6, 116.4; HRMS m/z (ESI) [M + NH_4_^+^]: calculated for C_9_H_8_N_3_O_2_, 190.0617; found, 190.0575.

### 4-Nitro-phthalimide (4l) (Song et al., 2018)

Yield, 10% (7.7 mg for 0.4 mmol); pale yellow powder; melting point, 196–198 °C (literature 198–202 °C); ^1^H NMR (400 MHz, DMSO-d_6_): δ 11.84 (s, 1H), 8.60 (d, *J* = 8.0 Hz, 1H), 8.43 (s, 1H), 8.07 (d, *J* = 8.0 Hz, 1H); ^13^C NMR (100 MHz, DMSO-d_6_): δ 167.7, 167.4, 151.4, 137.4, 134.1, 129.5, 124.6, 117.9.

### 4,5-Dimethoxyl-phthalimide (4m)

Yield, 70% (29.0 mg); white powder; melting point, > 200 °C (literature 318–320 °C); ^1^H NMR (400 MHz, DMSO-d_6_): δ 10.97 (s, 1H), 7.33 (s, 2H), 3.91 (s, 6H); ^13^C NMR (100 MHz, DMSO-d_6_): δ 169.3, 153.6, 125.7, 105.4, 56.3; HRMS m/z (ESI) [M + Na^+^]: calculated for C_10_H_9_N_1_O_4_Na, 230.0430; found, 230.0474.

### 7,8-Dihydro-1H-furo[3,2-e]isoindole-1,3(2H)-dione (4n)

Yield, 43% (16.3 mg); white powder; melting point, > 200 °C; ^1^H NMR (400 MHz, DMSO-d_6_): δ 11.11 (s, 1H), 7.55 (d, *J* = 8.0 Hz, 1H), 7.08 (d, *J* = 8.0 Hz, 1H), 4.73 (t, *J* = 8.8 Hz, 2H), 3.39 (t, *J* = 8.8 Hz, 2H); ^13^C NMR (100 MHz, DMSO-d_6_): δ 168.8, 166.0, 129.4, 125.7, 124.5, 123.9, 112.9, 73.2, 27.0; HRMS m/z (ESI) [M + 2Na^+^ - H^+^]: calculated for C_10_H_6_N_1_O_3_Na_2_, 234.0138; found, 234.0141.

## Results and Discussion

All the reactions were carried out under a dioxygen atmosphere in solvents (1 mL). We choose phenylacetonitrile (1a) as the model substrate and optimized the reaction by changing ingredients to find out the most efficient conditions (Table 1). Based on the yields obtained, it is found that the reaction of 1a (0.6 mmol), nitromethane (0.2 mmol), CuSO_4_.5H_2_O (0.2 mmol), 4-dimethylaminopyridine (DMAP, 0.4 mmol), and sodium hydroxide (0.4 mmol) in THF (1 mL) at 120 °C under dioxygen atmosphere for 24 h gives the best result, yielding the target product 2-hydroxyimino-2-phenylacetonitrile (2a) in 78% (entry 1). Other copper sources such as Cu(ClO_4_)_2_·6H_2_O and Cu(OTf)_2_ afford the compound 2a in similar yields (entries 2-3). However, the use of copper salt Cu(OAc)_2_·H_2_O for reaction would lead to the decline of yield to 54% (entry 4). It is proposed that the coordination interaction between the acetate ligands and the Cu(II) center was strong, which made the replacement of the OAc^−^ groups with the deprotonated nitromethane more difficult. Therefore, the formation of the active intermediate of [Cu^II^(DMAP)_2_(CH_2_NO_2_)]^−^ became difficult accordingly (see the following mechanism section, Scheme 6). No compound 2a could be isolated without the participation of copper salt or with the replacement of copper salts with NiSO_4_.6H_2_O and ZnSO_4_.7H_2_O (entries 6-7). The base of NaOH is found to be necessary for the processing of reaction. Both the strong base ^*t*^BuOK and the weak base K_2_CO_3_ resulted in the decreasing of yield to trace (entries 8-9). Interestingly, the use of the organic base of Et_3_N generated the product in a considerable good yield 68% (entry 10), probably due to the perfect solubility of it in THF. The type of solvent is essential for the production of 2a. The coupling reaction proceeds efficiently in THF, but it turns out to be difficult to process in the solvents of DMF, 1,4-dioxane, and toluene with the yields decreasing down to trace (entries 11–13). An excess of 1a (3 equivalents of nitromethane) would help to enhance the synthetic rate and increase the production of 2a (78%, entry 1), but overaddition of 1a (5 equivalents) did not do help to improve the rate of production (77%, entry 16). In contrast, the use of less amount of 1a, such as one equivalent or two equivalents, would decrease the yields down to 54% and 66% (entries 14-15), respectively. It has been shown that the strong base of EtONa and ^*t*^BuOK et al. would promote an extra transformation of phenylacetonitrile to β-enaminonitrile and/or 4-aminopyrimidine by mean of self-condensation (Zhu et al., 2019; Li et al., 2020), which would lead to the loss of the starting material of 1a unwillingly. In addition, the dioxygen atmosphere is crucial for the activation of nitromethane. The reaction in an atmosphere of air would lead to the decline of yield to 66%, and no product could be isolated from the reaction under N_2_ atmosphere (entries 17-18). This result indicates the role of dioxygen as an oxidant in the reaction. Finally, the influence of ligand on the production of 2a was examined with pyridine (L_2_), 4,4′-dimethyl-2,2′-bipyridyl (L_3_), 1,10-phenanthroline (L_4_), and *N*,*N*,*N*′,*N*′-tetramethylethylenediamine (L_5_). It showed that the production of 2a could be achieved with the employment of those ligands, but their yields were generally low at a level of *ca*. 30% (entries 19–22).

**TABLE 1:** Optimization of the formation of 2-hydroxyimino-2-phenylacetonitrile^a^.

En	Copper source	Base	Solvent	Amount of 1^a^ to nitro (equiv)	Atm	L	Yield^b^
1	CuSO_4_·5H_2_O	NaOH	THF	3	O_2_	L_1_	78%
2	Cu(ClO_4_)_2_·6H_2_O	NaOH	THF	3	O_2_	L_1_	74%
3	Cu(OTf)_2_	NaOH	THF	3	O_2_	L_1_	75%
4	Cu(OAc)_2_·H_2_O	NaOH	THF	3	O_2_	L_1_	54%
5	None	NaOH	THF	3	O_2_	L_1_	None
6	NiSO_4_·6H_2_O	NaOH	THF	3	O_2_	L_1_	None
7	ZnSO_4_·7H_2_O	NaOH	THF	3	O_2_	L_1_	None
8	CuSO_4_·5H_2_O	^*t*^BuOK	THF	3	O_2_	L_1_	Trace
9	CuSO_4_·5H_2_O	K_2_CO_3_	THF	3	O_2_	L_1_	Trace
10	CuSO_4_·5H_2_O	Et_3_N	THF	3	O_2_	L_1_	68%
11	CuSO_4_·5H_2_O	NaOH	DMF	3	O_2_	L_1_	Trace
12	CuSO_4_·5H_2_O	NaOH	Dioxane	3	O_2_	L_1_	30%
13	CuSO_4_·5H_2_O	NaOH	Toluene	3	O_2_	L_1_	Trace
14	CuSO_4_·5H_2_O	NaOH	THF	1	O_2_	L_1_	54%
15	CuSO_4_·5H_2_O	NaOH	THF	2	O_2_	L_1_	66%
16	CuSO_4_·5H_2_O	NaOH	THF	5	O_2_	L_1_	77%
17	CuSO_4_·5H_2_O	NaOH	THF	3	Air	L_1_	68%
18	CuSO_4_·5H_2_O	NaOH	THF	3	N_2_	L_1_	None
19	CuSO_4_·5H_2_O	NaOH	THF	3	O_2_	L_2_	30%
20	CuSO_4_·5H_2_O	NaOH	THF	3	O_2_	L_3_	27%
21	CuSO_4_·5H_2_O	NaOH	THF	3	O_2_	L_4_	25%
22	CuSO_4_·5H_2_O	NaOH	THF	3	O_2_	L_5_	37%

^a^ Reaction conditions: 1a (0.6 mmol), CH_3_NO_2_ (0.2 mmol), CuSO_4_·5H_2_O (0.2 mmol), DMAP (0.4 mmol), base (0.2 mmol), solvent (1 mL), and 120 °C.

^b^ Isolated yield. DMAP = 4-dimethylaminopyridine; nitro = nitromethane; atm = atmosphere; L = ligand.

Table 1. Optimization of the formation of 2-hydroxyimino-2-phenylacetonitrile.

**SCHEME 5:**
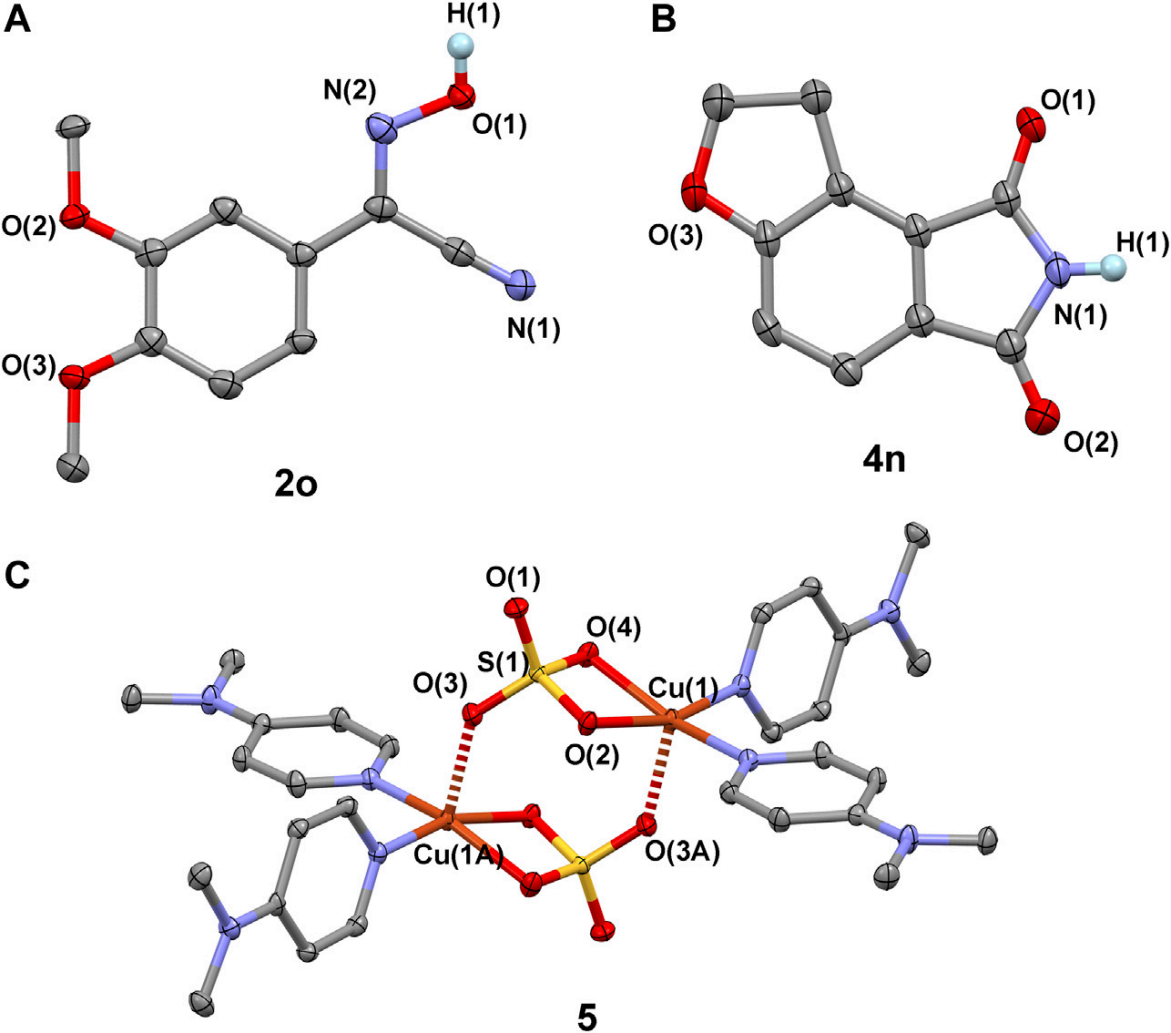
Crystal structures of compounds 2o (CCDC 2007798), 4n (CCDC 2007797), and 5 (CCDC 2007804), showing the thermal ellipsoids of 50% probability surfaces. The weak interactions between molecules with the Cu(1)-O(3A) and Cu(1A)-O(3) bond lengths of 2.193(1) Å are shown.

Under the optimal conditions, we set out to examine the scope of phenylacetonitriles for the generation of benzyl-cyano-oxime compounds (Scheme 2). A total of 26 reactions were carried out for the analysis of the implication of reaction, including twenty-one phenylacetonitrile substrates (2a–2u) and five nitro compounds (I–V). The yield distribution and production efficiency of 2-hydroxyimino-2-phenylacetonitriles (2) are discussed based on the electronic effect, steric effect, and the synergic effect of functional groups on the nitriles. Firstly, the experimental results show that the electron-donating groups would help to improve the yields of compounds (2a–2e) compared to the electron-withdrawing groups (2g–2i, 2l), with the yield difference of *ca*. 25% between them. The phenyl-group-substituted phenylacetonitrile afforded the product in a moderate yield of 60% (2f). Secondly, the steric hindrance to the reaction was studied by employing methyl group (2b-2d) and fluoro group (2i–2k) in the para-, meta-, and orthopositions of phenyl group, respectively. It is found that the size of methyl group or fluoro group is not important for the production of compounds (2b-2d, 2i–2k), but the difunctional groups would lead to a drop of yields about 20% correspondingly (2m–2q) (Scheme 5A). Thirdly, the coupling reaction was examined with varied aromatic-group-substituted acetonitriles as substrates, for example, naphthyl acetonitriles and thienyl acetonitriles. All the reactions could succeed in the production of cyano-oxime compounds, but the efficiency of transformation was relatively low, and the products were obtained in yields about 30% (2r–2u). It is clear that the deprotonation of aromatic acetonitrile is a key step for the formation of 2-hydroxyimino-2-phenylacetonitriles in our system. The reaction of the methyl-substituted phenylacetonitrile in the position of carbon proximal to the cyanide group did not generate the corresponding compound at all. In addition, the scope of nitroalkanes was also examined for comparison. Five nitro compounds, *e*.*g*., nitroethane, 1-nitropropane, 2-nitropropane, 1-nitrobutane, and 2-methyl-2-nitropropane, were selected to examine the percent of the transformation efficiency from nitro to oxime. It is shown that the nitro compounds with *α*-hydrogen on the carbon atom proximal to the cyanide group provided the product 2a in moderate yields (Schemes 2I–V), while no product could be isolated with the use of 2-methyl-2-nitropropane as a starting material (Scheme 2VI). It is proposed that the initial deprotonation of the *α*-H to the nitro group is critical for the activation of nitro compounds, by which the nitro group would transfer to nitroso group *via* oxidation and nitrification reactions (see below).

Interestingly, our reaction can also be extended to the formation of functional phthalimides (Scheme 3). Twenty-seven reactions were carried out to examine the transformation of 1-indanones to phthalimides, by using nitromethane as nitrogen donor. The experimental results show that the electronic effect of substituted groups on the phenyl ring of 1-indanones has a significant impact on the production of phthalimides (4). The reaction of 1-indanones, with electron-donating groups (H-, Me-, Me_3_C-, MeO-, PhCH_2_O-) and weak electron-withdrawing groups (Ph-, Br-) on the 6′-position of phenyl ring, afforded the phthalimides in moderate to high yields (75–80%; 4a and 4c-4h). The 4′-methyl group substituted indanone provided lower production efficiency (53%), probably due to the steric effect on the activation of the 3-carbon of indanone (4b). In contrast, the production of four would be influenced by the introduction of medium electron-withdrawing groups to the phenyl ring. The reaction of 1-indanones with the substituted chloro and fluoro groups in the 6′-position of phenyl ring afforded the product in lower yields 71% and 65% (4i and 4j), respectively. With the strong electron-withdrawing groups, such as cyano and nitro groups, the substituted 1-indanones would only be able to offer the products in yields about 10% (4k and 4l). In addition, the disubstitution of the phenyl ring of 1-indanones would have a negative influence on the yield of product. The dimethoxyl-group-substituted 1-indanone generated the compound 4m in a lower yield (70%) compared to the single methoxyl group substituted 1-indanone (4e, 80%). The disubstituted groups in the 6′,7′-positions of 1-indanone led to the decline of yield dramatically down to 43% (4n) (Scheme 5B). This point of view is consistent with the result presented in the formation of 4b that the substitution on the carbon atoms adjacent to the five-member ring of 1-indanones would significantly decrease the yields of phthalimides in our system.

Furthermore, the reactions of 5′-substituted 1-indanones with methyl, methoxyl, and fluoro groups also generated the phthalimides in good yields (Scheme 3, Part b, I–III), even though they have the same structures to compounds 4c, 4e, and 4j. Apart from the 1-indanone with a 5-member ring on the side of ketone, the 1-tetralone with a 6-member ring and the 1-benzosuberone with a 7-member ring are also able to produce the compound 4a under the standard reaction conditions (Scheme 3, Part b, IV-V). It implies that the methylene groups next to the phenyl ring of 1-indanone and its derivatives (3′-carbon in 1-indanone; 4′-carbon in 1-tetralone; 5′-carbon in 1-benzosuberone) would be oxidized in the processing of reaction. The cleavage of C-C bond between the ketone groups and the unsaturated carbon atoms adjacent to them (1′- and 2′-carbon and 2′- and 3′-carbon in 1-indanone; 1′- and 2′-carbon and 3′- and 4′-carbon in 1-tetralone; 1′- and 2′-carbon and 4′- and 5′-carbon in 1-benzosuberone) would occur with the insertion of nitrogen atom. This inference is consistent with our experimental results that the reactions of indan-1,2-dione, indan-1,3-dione, and 1,2,3-indantrione (ninhydrin) in our system are also able to provide the product 4a (Scheme 3, Part b, VI–VIII). Finally, other organic nitro compounds, such as nitroethane, 1-nitropropane, 2-nitropropane, and 1-nitrobutane, are also suitable for the formation of compound 4a, with the yields decreasing gradually from 65% to 30% (Scheme 3, Part c, I–V). No reaction occurred with the use of 2-methyl-2-nitropropane as a nitrogen donor (Scheme 3, Part c, VI).

**SCHEME 6:**
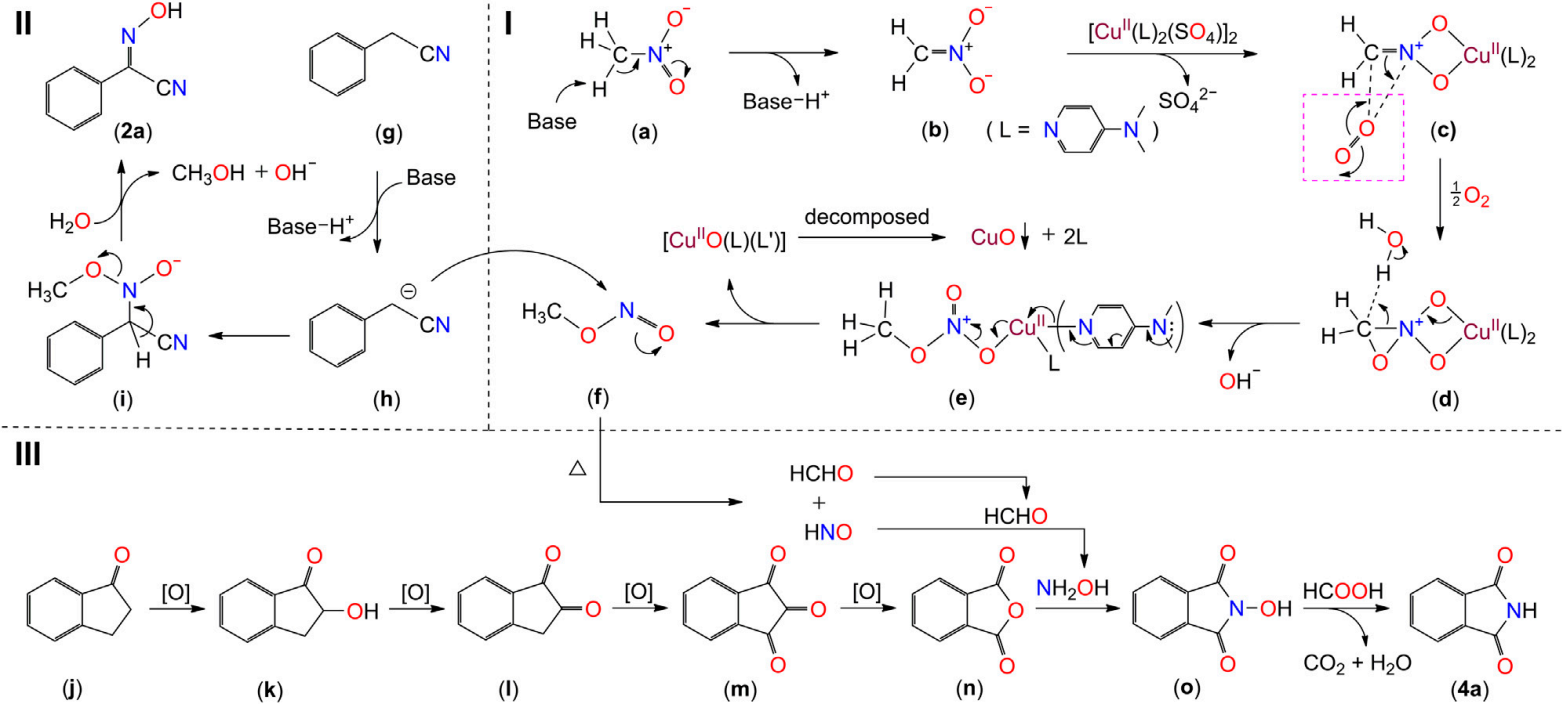
The plausible mechanism of the transformation of nitromethane to 2-hydroxyimino-2-phenylacetonitrile (2a) and phthalimide (4a). The image in the dashed-line-box of (c) is shown for illustrative purposes, displaying how the electron transfer occurs.

The above experiment results aroused our great interest in exploring the reaction mechanism. A number of reactions were carried out for a better understanding of how the reactions occurred (Scheme 4). Firstly, the species of 1-indanone was reduced to form an aromatic hydrocarbon compound, indanone, which was found not able to generate the compound 4a under our conditions. It implies that the presence of carbonyl group in 1′or 3′-position of indanone is required for the processing of reaction (Scheme 4A). Secondly, the oxidation of aromatic ketone in our system was examined in the solution of 2-methyl-2-nitropropane (Me_3_C-NO_2_). The solvent of 2-methyl-2-nitropropane was used with the aim of maintaining the condition of the solvent close to nitromethane without the presence of *α*-H. The reaction of 1-indanone with Me_3_C-NO_2_ under our conditions afforded phthalic acid (3′) rather than phthalimide (4a) after acidification (Scheme 4B). No phthalic acid could be obtained without the addition of CuSO_4_.5H_2_O and DMAP under dinitrogen atmosphere (Scheme 4C). This result indicates that the oxidation of 1-indanone to phthalic acid could be achieved by the activate oxygen, probably coordinating on the Cu(II) intermediate (Wang et al., 2015). The aromatic ketone species underwent a C-C bond cleavage to form an intermediate species of *o*-phthalic anhydride during the reaction, and the insertion of nitrogen donor occurred with the species of *o*-phthalic anhydride (Scheme 6). Thirdly, the activation of nitromethane was investigated by using phthalic acid and/or *o*-phthalic anhydride as the starting materials. The experimental results showed that the reaction of phthalic acid or *o*-phthalic anhydride under the standard conditions afforded the phthalimide in yield 68% and 75%, respectively (Schemes 4D,E). No product could be isolated in the absence of CuSO_4_.5H_2_O and DMAP under dinitrogen atmosphere (Scheme 4F). That is to say, the combination of Cu(II) salt, DMAP, and dioxygen facilitated the activation of nitromethane. Meanwhile, the reaction of *o*-phthalic anhydride with butyl nitrite under the same conditions provided the target product 2a in yield 70% (Scheme 4G). The reaction of phenylacetonitrile with butyl nitrite afforded the compound 4a in yield 65% (Scheme 4H). It is noted that the butyl nitrite was used instead of methyl nitrite for the reasons of commercial availability, easy handling, and same performance. Thus, the methyl nitrite is thought to be an intermediate of the reaction in the process of the oxidation of nitromethane, which resembles the research of the oxygen consumption in the oxidation of nitromethane by visible light irradiation of Rose Bengal (Bilski et al., 1994). Fourthly, the stirring of CuSO_4_.5H_2_O and DMAP in nitromethane at room temperature formed a dark green solution, from which a dinuclear copper(II) species of [Cu^II^(DMAP)_2_(SO_4_)] 5) (Scheme 5C) was isolated by diffusion of Et_2_O into the reaction solution (Scheme 4I). The reactions of 1-indanone or phenylacetonitrile with nitromethane in the presence of compound 5 generated the products 4a and 2a in similar yields (Schemes 4J,K). It implies that compound 5 would be an intermediate of activated copper(II) species for the transformation of nitromethane to methyl nitrite.

Based on the above results, a plausible mechanism is depicted in Scheme 6. The model of the reaction is divided into three parts, the transformation of nitromethane to methyl nitrite (Scheme 6-(I)), the formation of 2-hydroxyimino-2-phenylacetonitrile (Scheme 6-(II)), and the generation of phthalimide (Scheme 6-(III)). The deprotonation of CH_3_NO_2_ molecule (a) leads to the generation of intermediate (b), by which intermediate (c) is formed with the participation of [Cu^II^(DMAP)_2_(SO_4_)]. The latter is produced simultaneously by the reaction of CuSO_4_.5H_2_O with two equivalents of DMAP (Scheme 5C). Two molecules of (c) react with one equivalent of dioxygen to generate the intermediate (e), through the transition state of (d). Methyl nitrite (f) is generated with the release of [Cu^II^O(L) (L′)]^2+^ (L = DMAP) into the solution (Scriven, 1983; Ragnarsson and Grehn, 1998). The [Cu^II^O(L) (L’)]^2+^ species undergoes a decomposition to deposit the metal moiety as a black precipitate CuO. The decomposition process is not clear to us.

On one hand, one of hydrogen atoms on the methylene group of phenyl acetonitrile molecule (g) is deprotonated to form a cyanoalkylide anion (h). The anion attacks an adjacent CH_3_ONO molecule on the nitrogen atom to form the intermediate species (i). The deprotonation of the second hydrogen atoms of the methylene group of (i) with the transfer of the pair electrons to the C-N bond generates the compound 2a (Scheme 6-(II)). On the other hand, the continuous oxidation of 1-indanone (j) in the presence of [Cu^II^(DMAP)_2_(SO_4_)] and dioxygen generates the intermediate species *o*-phthalic anhydride (n) (Scheme 6-(III)) (Wang et al., 2015). Meanwhile, the methyl nitrite (f) undergoes a thermal decomposition reaction to generate the species HCHO and HNO (Brower, 1988; Zhu et al., 2013). The HNO is reduced to NH_2_OH in the presence of formaldehyde, and the generated NH_2_OH is inserted into the intermediate *o*-phthalic anhydride (n) to form the species (o). The species (o) is reduced by the generated formic acid from the oxidation of formaldehyde (Liu et al., 2019), forming the final product phthalimide (4a).

## Conclusion

In summary, we have developed a simple and efficient method for the synthesis of 2-(hydroxyimino)-2-phenylacetonitriles and phthalimides in a total of 51 samples, by using nitromethane as the nitrogen donor. The production of two types of compounds indicates the flexible and diversified characteristics of our system in the activation of nitromethane for the synthesis of N-containing compounds. The extra diversity and stability of compounds are discussed in terms of electronic effect, steric effect, position of substituted groups, and intramolecular charge transfer. The mechanism study shows that the methyl nitrite is generated as an intermediate in the reaction, and the transformation of nitromethane to methyl nitrite is promoted by the Cu(II) intermediate with the participation of dioxygen as oxidant. The proposed nitromethane transformation process is supported by the experimental results. The employment of easily available reagents and mild reaction condition makes this method more interesting for chemists. This work might provide a clue to apply nitromethane as a nitrogen donor in the development of organic synthetic chemistry, biological chemistry, and pharmaceutical chemistry.

## Data Availability Statement

The original contributions presented in the study are included in the article/Supplementary Material; further inquiries can be directed to the corresponding author.

## Author Contributions

SX contributed to the full experimental part and initial draft. YL contributed to the results discussion of Scheme 2. WF contributed to the results discussion of Scheme 3. JJ and WZ contributed to the result discussion of Scheme 4. DH contributed to full organization, supervision, writing of final version of the manuscript, and submission.

## Funding

This research was supported by the National Natural Science Foundation of China (Grant no. 21371171) and the Strategic Priority Research Program of the Chinese Academy of Sciences (Grant no. XDB20000000).

## Conflicts of Interest

The authors declare that the research was conducted in the absence of any commercial or financial relationships that could be construed as a potential conflict of interest.
