# Supplementary material for: Copper(II)-Dioxygen Facilitated Activation of Nitromethane: Nitrogen Donors for the Synthesis of Substituted 2-Hydroxyimino-2-phenylacetonitriles and Phthalimides
Source: Front Chem. 2021 Jan 29;8:622867. doi: 10.3389/fchem.2020.622867 (PMC7878530; doi:10.3389/fchem.2020.622867)
Supplement: Supplementary file 1 [file datasheet1.docx]

**Crystallographic Data of Compounds**

**Table S1** Crystallographic data*^a^* for the compounds of **2a**, **2j**, **2o**, and **4b**.

| Compounds | **2a** (2007801) | **2j** (2007799) | **2o** (2007798) | **4b** (2007796) |
| --- | --- | --- | --- | --- |
| formula | C_8_H_6_N_2_O | C_8_H_5_FN_2_O | C_12_H_16_N_2_O_4_S | C_9_H_7_NO_2_ |
| *M* | 146.15 | 164.14 | 284.33 | 161.16 |
| crystal system | Monoclinic | Monoclinic | Orthorhombic | Monoclinic |
| space group | P2_1_/c | P2_1_/n | Pna2_1_ | P2_1_/n |
| *a*, Å | 8.6866(6) | 3.7099(3) | 17.4948(9) | 7.1869(7) |
| *b*, Å | 11.0479(5) | 16.9111(15) | 4.6370(3) | 4.9108(5) |
| *c*, Å | 8.2038(5) | 11.8106(9) | 16.9301(8) | 21.2924(18) |
| α, deg | 90 | 90 | 90 | 90 |
| *β*, deg | 115.590(8) | 97.448(7) | 90 | 91.604(9) |
| *γ*, deg | 90 | 90 | 90 | 90 |
| *V*, Å^3^ | 710.08(7) | 734.73(10) | 1373.42(13) | 751.18(12) |
| *Z* | 4 | 4 | 4 | 4 |
| *μ*, mm^-1^ | 0.774 | 1.018 | 2.220 | 0.849 |
| independent data | 1332 | 1382 | 1861 | 1410 |
| refined parameters | 100 | 109 | 172 | 110 |
| *R*_1_*^b^*, *wR*_2_*^c^* (I >2σ(I)) | 0.0382, 0.1041 | 0.0840, 0.2596 | 0.0451, 0.1190 | 0.0516, 0.1555 |
| *R*_1_, *wR*_2_  (all data) | 0.0453, 0.1078 | 0.1049, 0.2646 | 0.0507, 0.1232 | 0.0645, 0.1697 |

*^a^*T = 100(2) K, Cu Kα radiation (*λ* = 1.54184 Å). *^b^R*_1_ = ∑||*F*_o_| – | *F*_c_|| / ∑|*F*_o_|. *^c^wR*_2_ = {∑[*w*(*F*_o_^2^ – *F*_c_^2^)^2^/(*F*_o_^2^)^2^]}^½^.

**Table S2** Crystallographic data*^a^* for the compounds of **4e**, **4i**, **4n**, and **5**.

| Compounds | **4e** (2007792) | **4i** (2007794) | **4n** (2007797) | **5** (2007804) |
| --- | --- | --- | --- | --- |
| formula | C_9_H_7_NO_3_ | C_8_H_4_ClNO_2_ | C_10_H_7_NO_3_ | C_34_H_54_Cu_2_N_10_O_10_S_2_ |
| *M* | 177.16 | 181.57 | 189.17 | 954.07 |
| crystal system | Monoclinic | Triclinic | Triclinic | Triclinic |
| space group | P2_1_/n | P-1 | P-1 | P-1 |
| *a*, Å | 6.24453(11) | 5.7786(6) | 5.2597(4) | 9.0156(5) |
| *b*, Å | 8.24959(11) | 7.7921(9) | 8.4520(10) | 10.9206(5) |
| *c*, Å | 15.3916(2) | 8.4411(11) | 10.1210(13) | 11.5562(6) |
| α, deg | 90 | 82.453(11) | 112.814(12) | 102.488(4) |
| *β*, deg | 100.5217(15) | 75.879(10) | 99.476(9) | 108.475(5) |
| *γ*, deg | 90 | 89.225(9) | 92.990(8) | 101.258(4) |
| *V*, Å^3^ | 779.56(2) | 365.34(7) | 405.73(8) | 1009.97(9) |
| *Z* | 4 | 2 | 2 | 1 |
| *μ*, mm^-1^ | 0.974 | 4.239 | 0.980 | 2.846 |
| independent data | 1480 | 1360 | 1529 | 3791 |
| refined parameters | 118 | 109 | 128 | 268 |
| *R*_1_*^b^*, *wR*_2_*^c^* (I >2σ(I)) | 0.0343, 0.0848 | 0.0447, 0.1170 | 0.0384, 0.0977 | 0.0290, 0.0763 |
| *R*_1_, *wR*_2_  (all data) | 0.0346, 0.0849 | 0.0613, 0.1235 | 0.0465, 0.1040 | 0.0315, 0.0781 |

*^a^*T = 100(2) K, Cu Kα radiation (*λ* = 1.54184 Å). *^b^R*_1_ = ∑||*F*_o_| – | *F*_c_|| / ∑|*F*_o_|. *^c^wR*_2_ = {∑[*w*(*F*_o_^2^ – *F*_c_^2^)^2^/(*F*_o_^2^)^2^]}^½^.


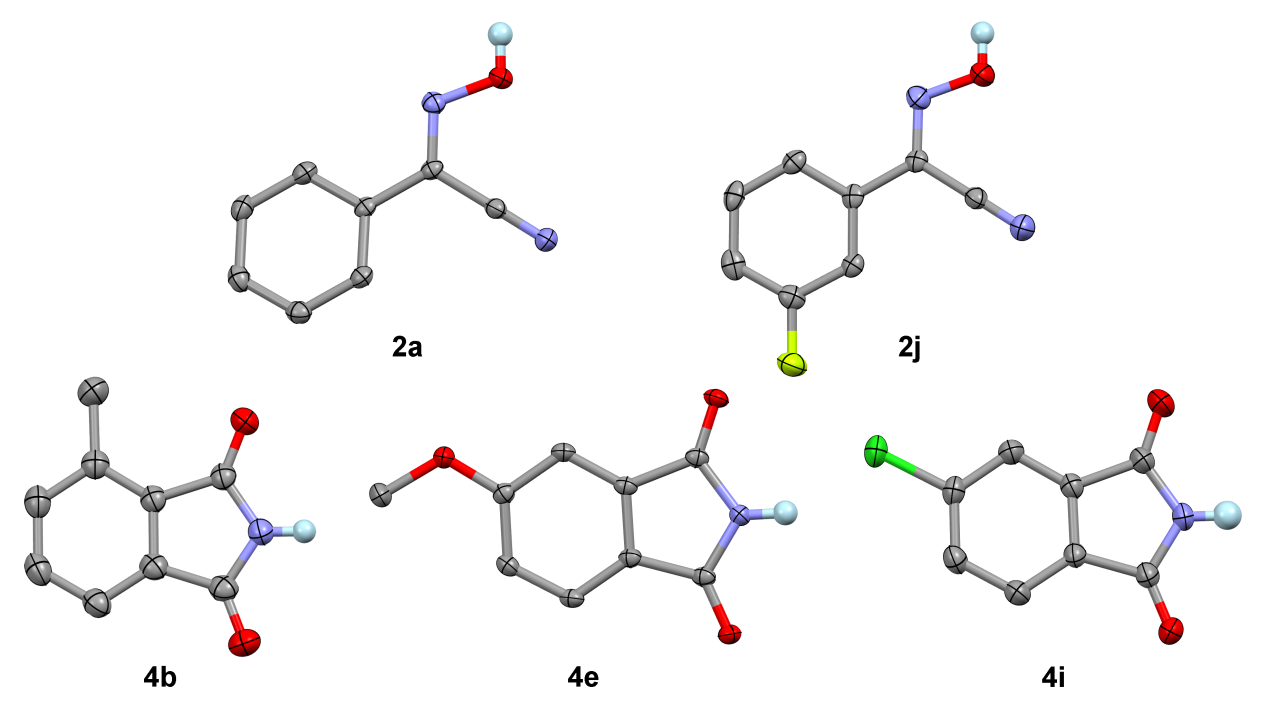


**Fig. S1** Crystal structures of compounds **2a** (CCDC 2007801), **2j** (CCDC 2007799), **4b** (CCDC 2007796), **4e** (CCDC 2007792), and **4i** (CCDC 2007794), showing the thermal ellipsoids of 50% probability surfaces.
