# Supplementary material for: Copper(II)-Dioxygen Facilitated Activation of Nitromethane: Nitrogen Donors for the Synthesis of Substituted 2-Hydroxyimino-2-phenylacetonitriles and Phthalimides
Source: Front Chem. 2021 Jan 29;8:622867. doi: 10.3389/fchem.2020.622867 (PMC7878530; doi:10.3389/fchem.2020.622867)
Supplement: Supplementary file 2 [file datasheet2.docx]

Supplementary Material

**NMR spectra**

**2-(hydroxyimino)-2-phenylacetonitrile (2a)**


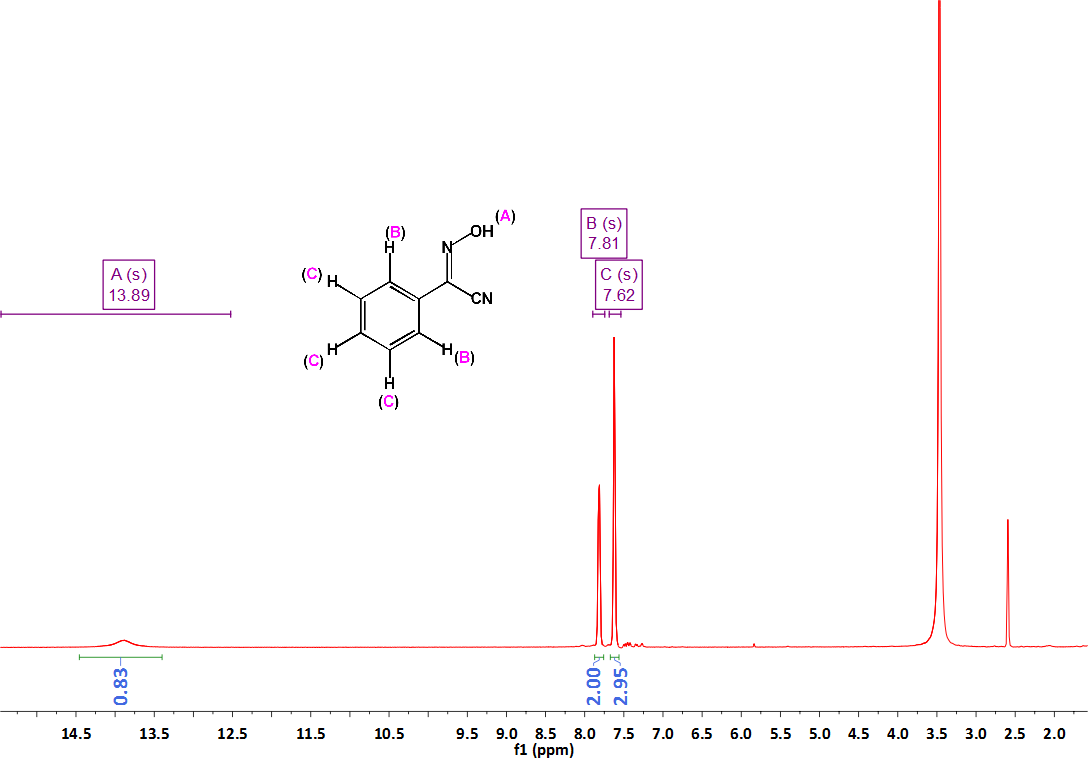


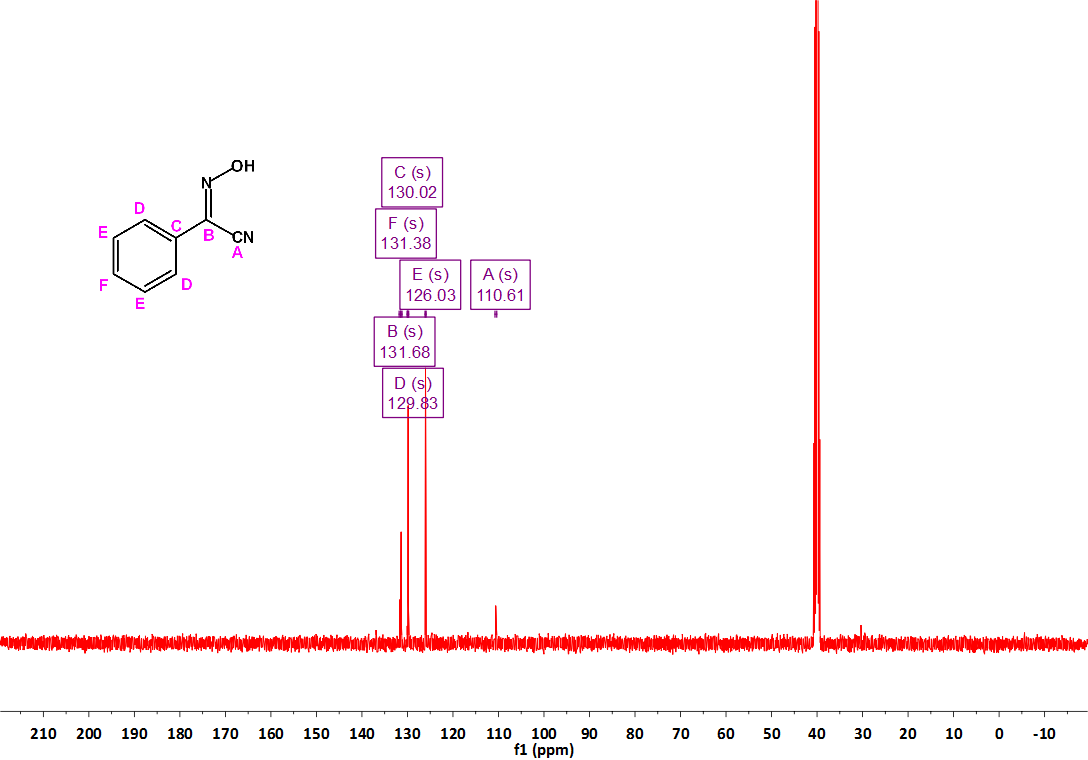


**2-(hydroxyimino)-2-(2-methyl)-phenylacetonitrile (2b)**

**
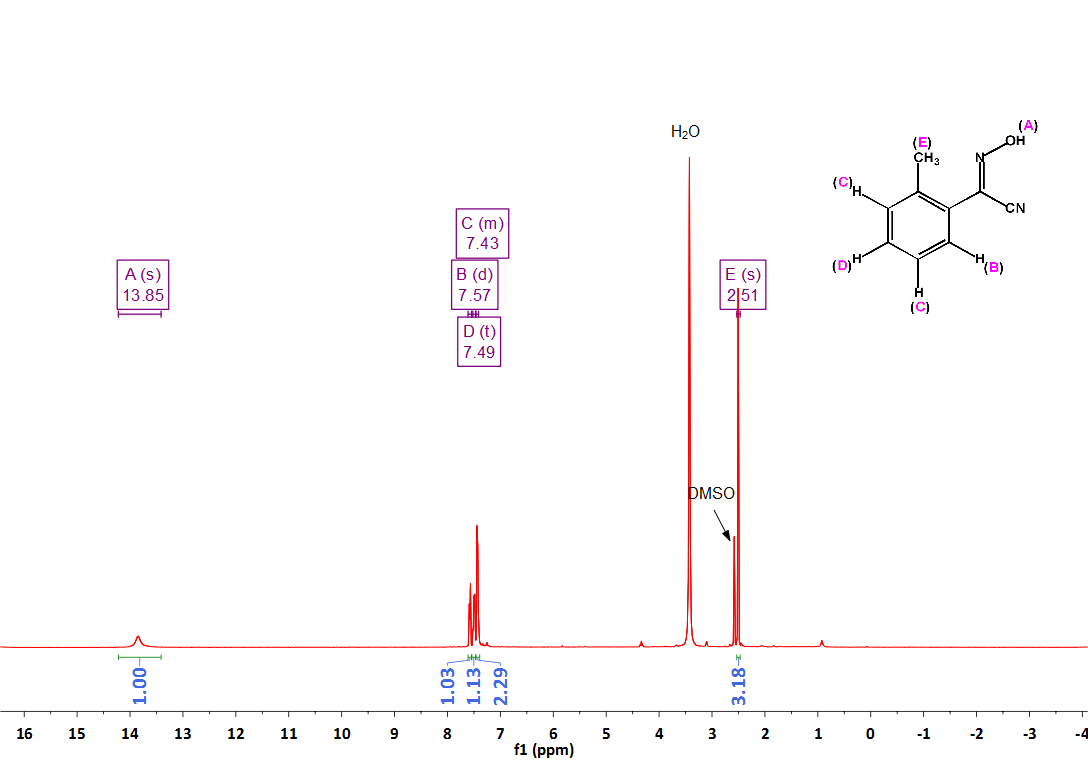
**

**
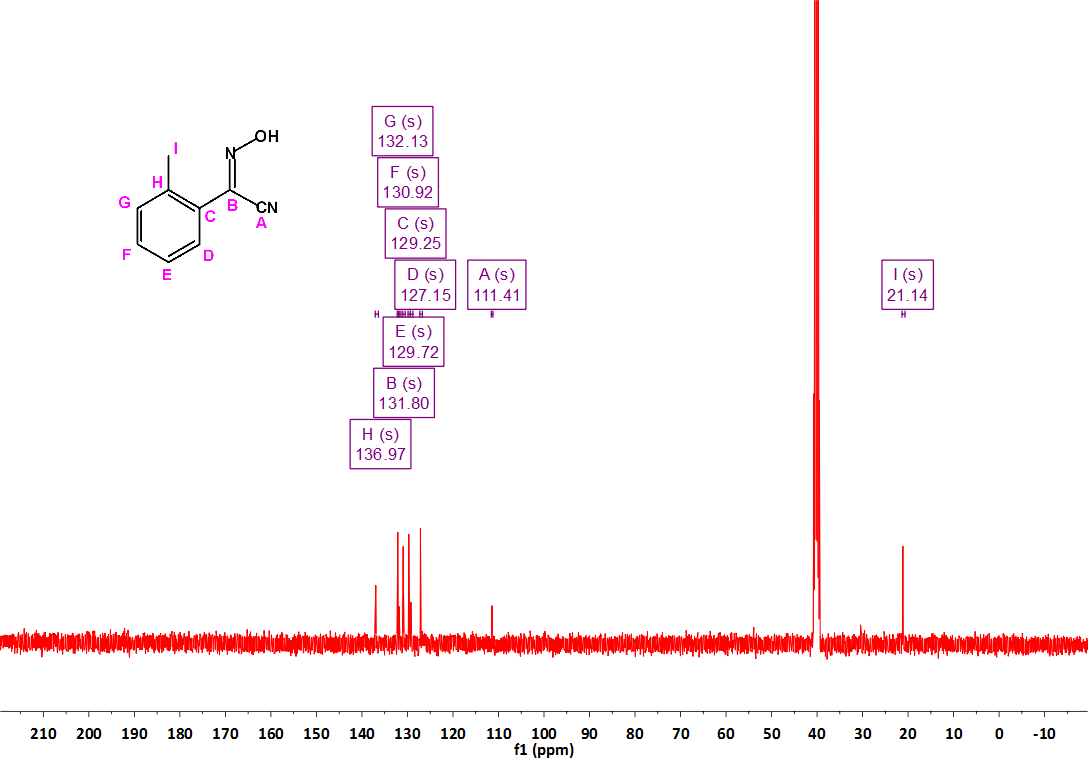
**

**2-(hydroxyimino)-2-(3-methyl)-phenylacetonitrile (2c)**

**
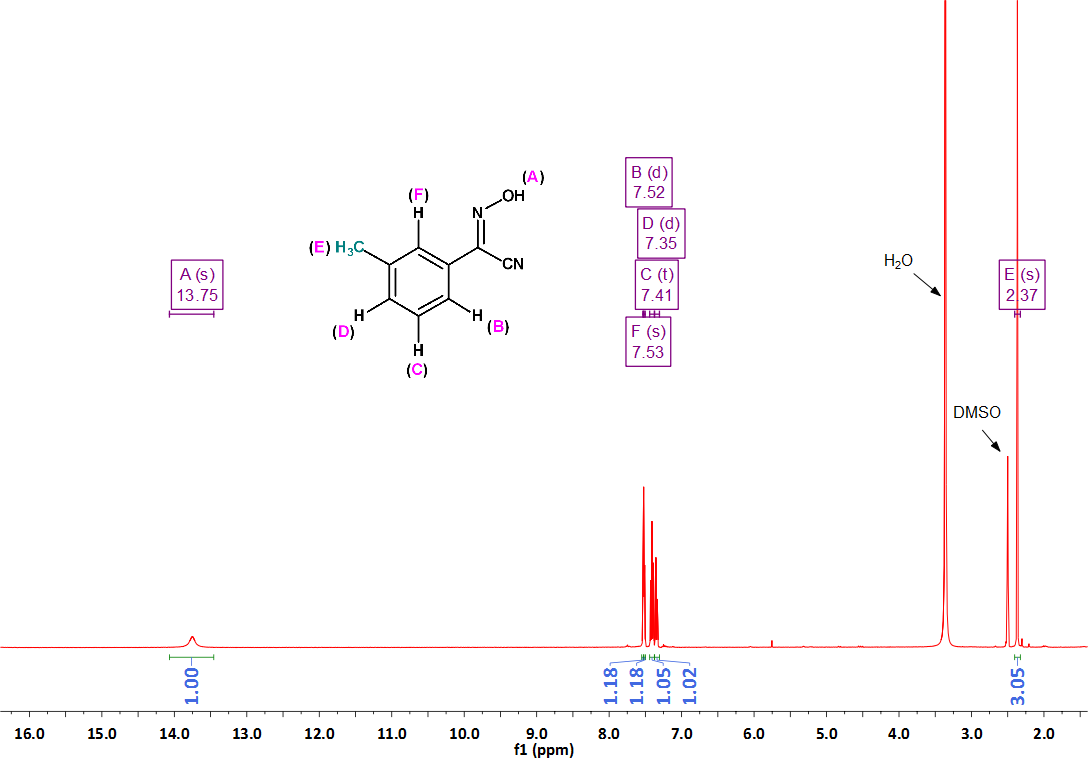
**

**
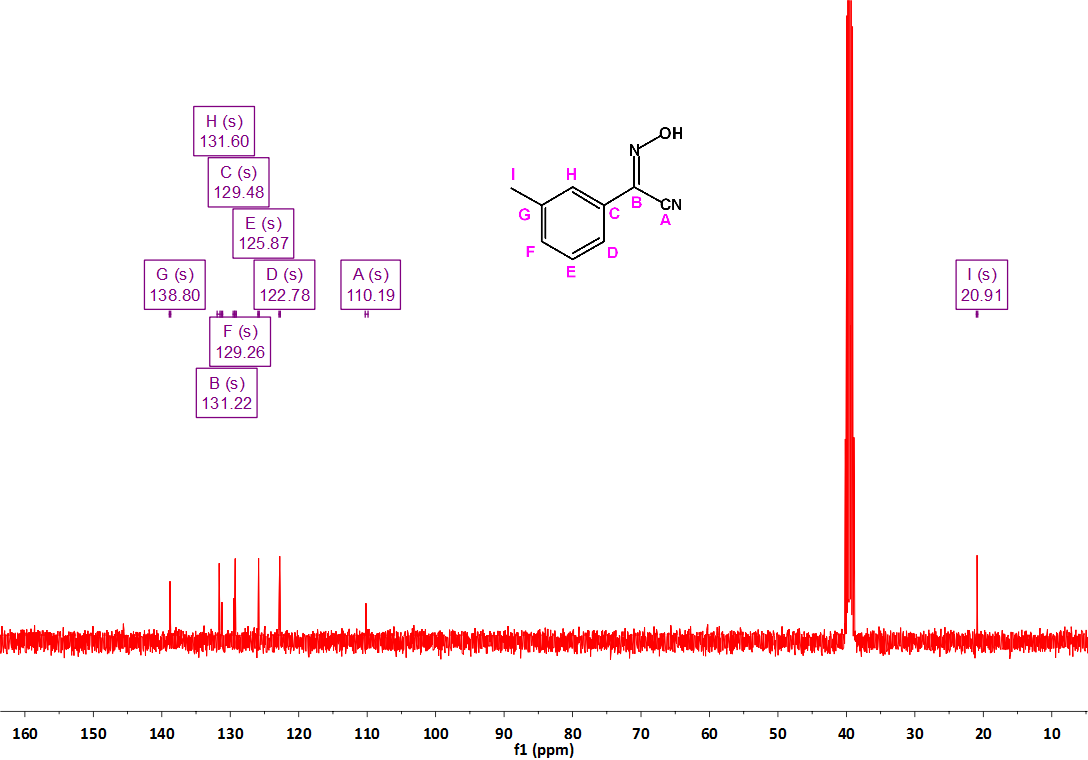
**

**2-(hydroxyimino)-2-(4-methyl)-phenylacetonitrile (2d)**

**
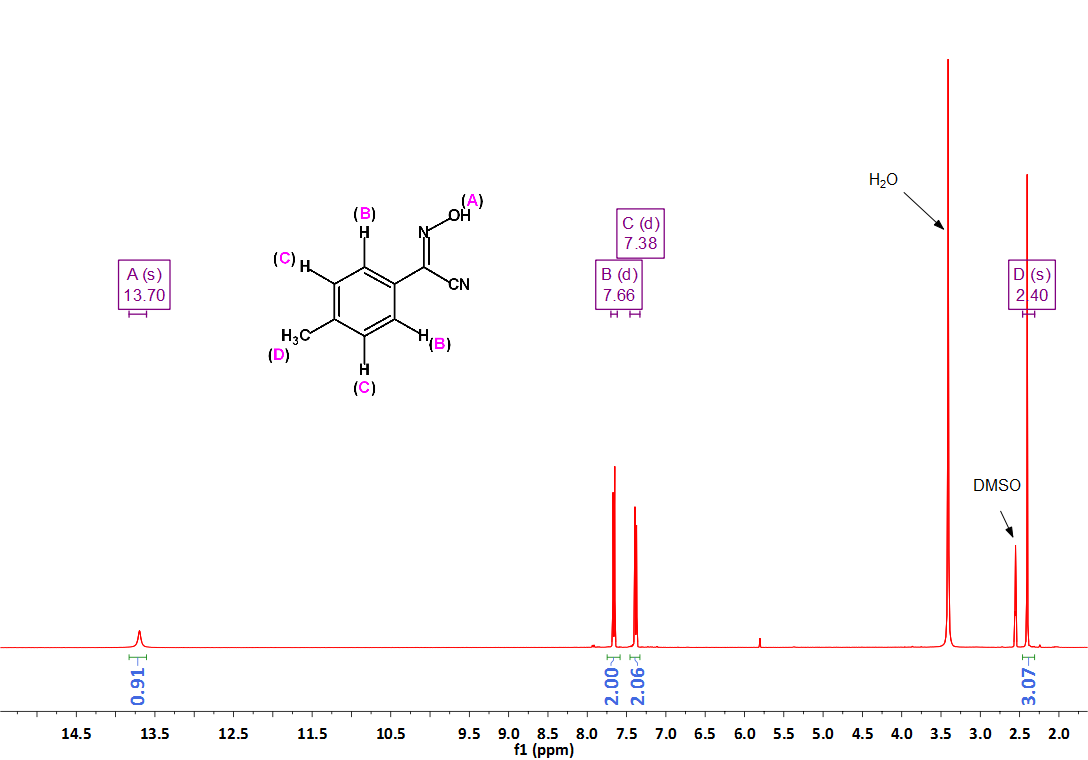
**

**
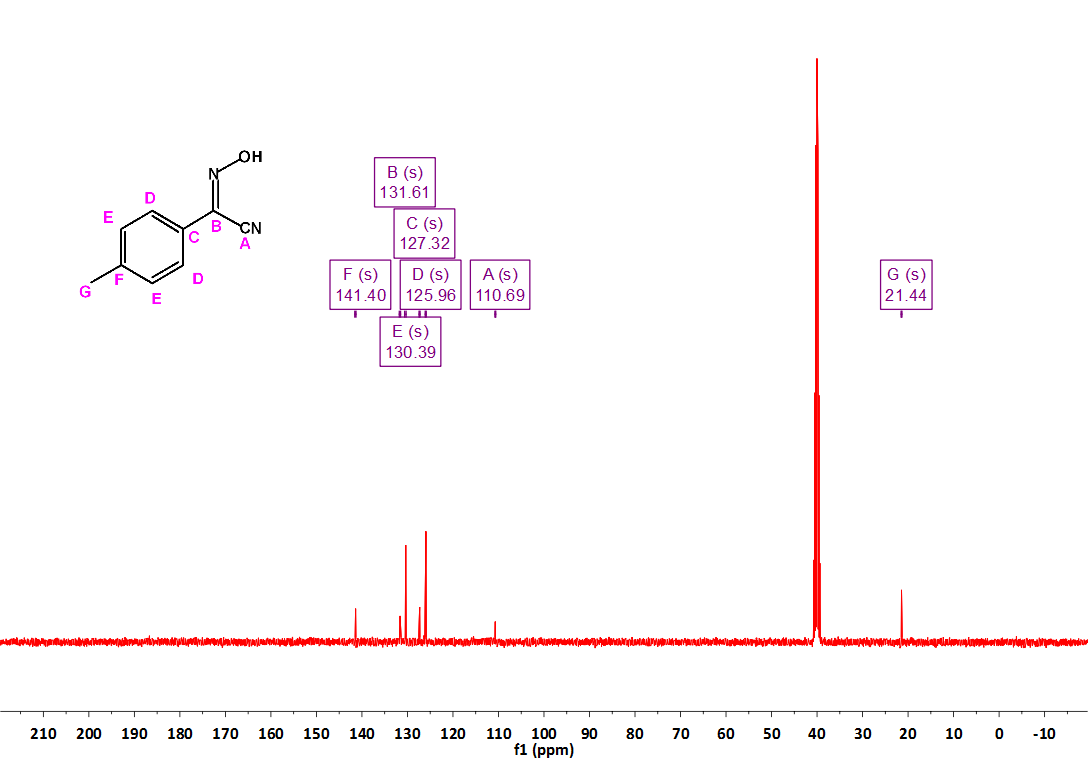
**

**4-(tert-butyl)-N-hydroxybenzimidoyl cyanide (2e)**

**
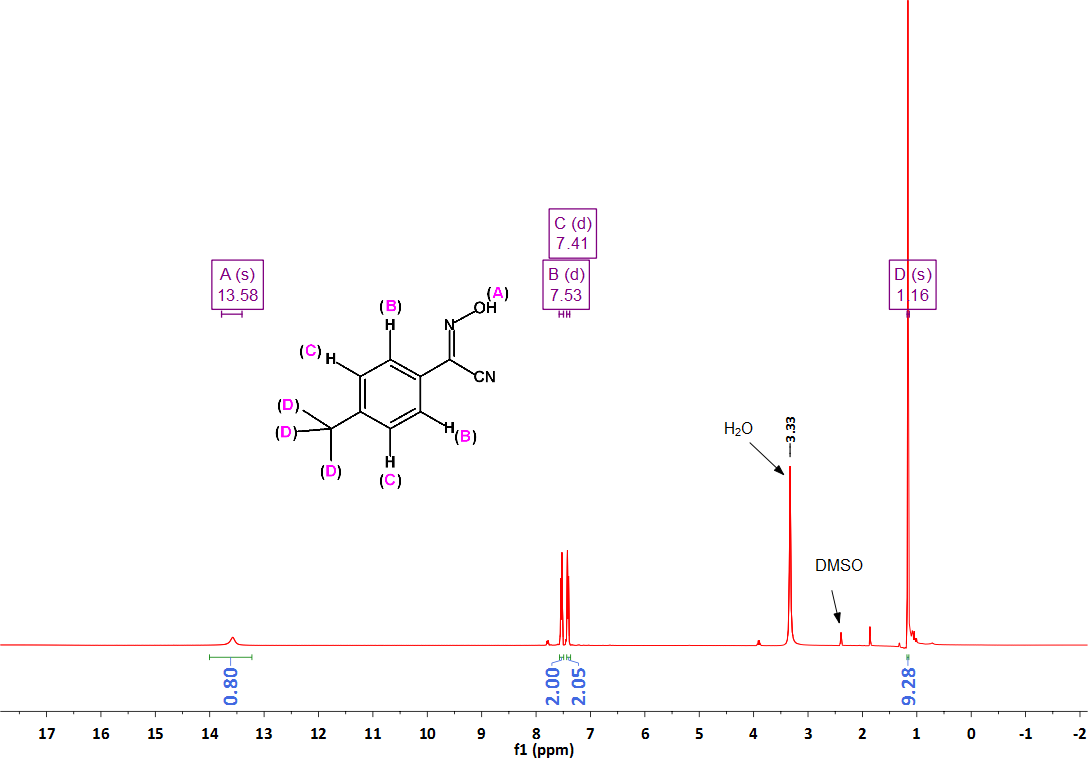
**

**
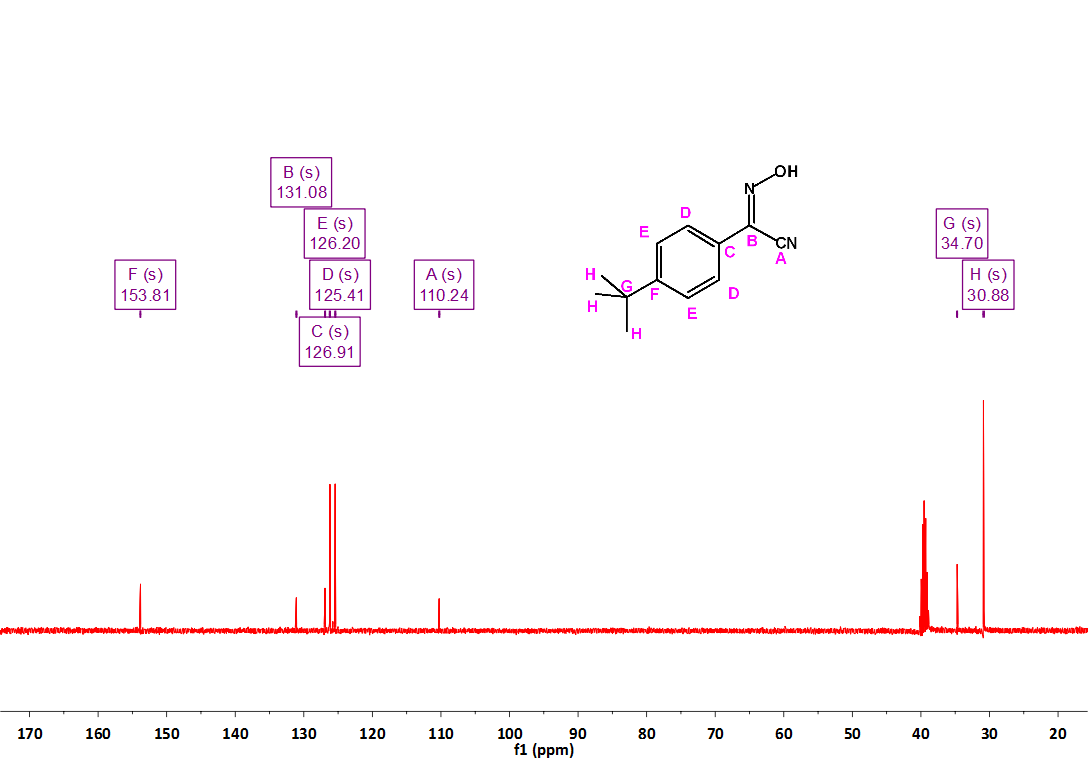
**

**2-(hydroxyimino)-2-(4-phenyl)-phenylacetonitrile (2f)**

**
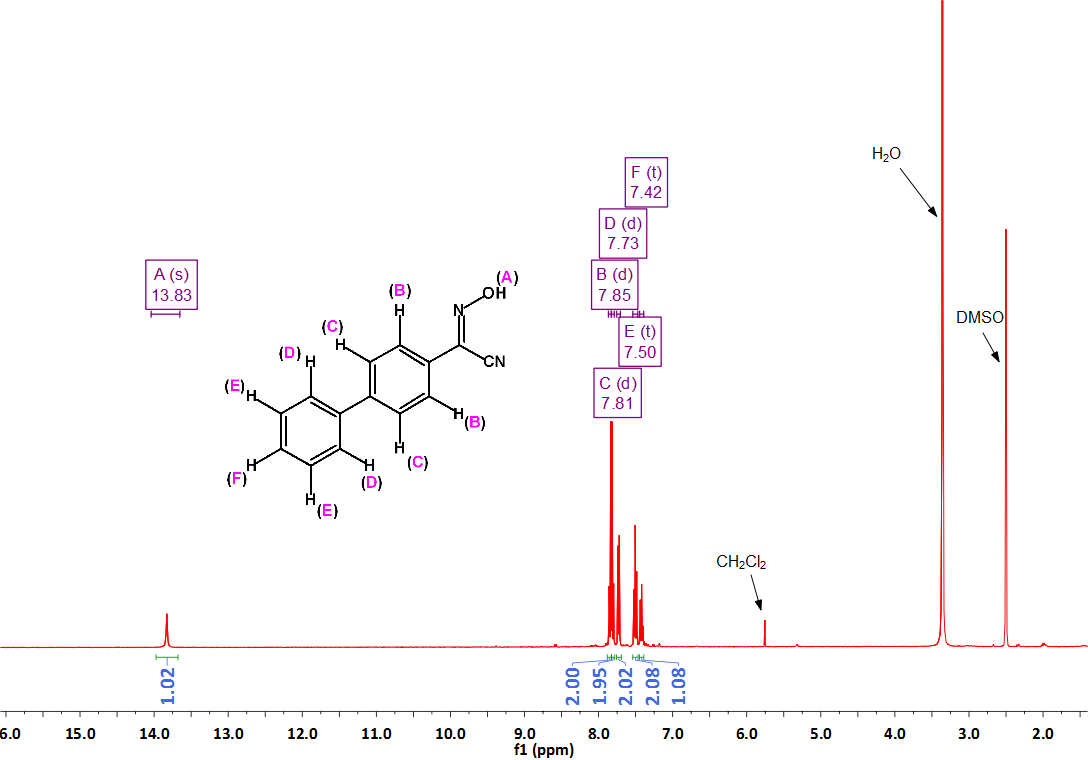
**

**
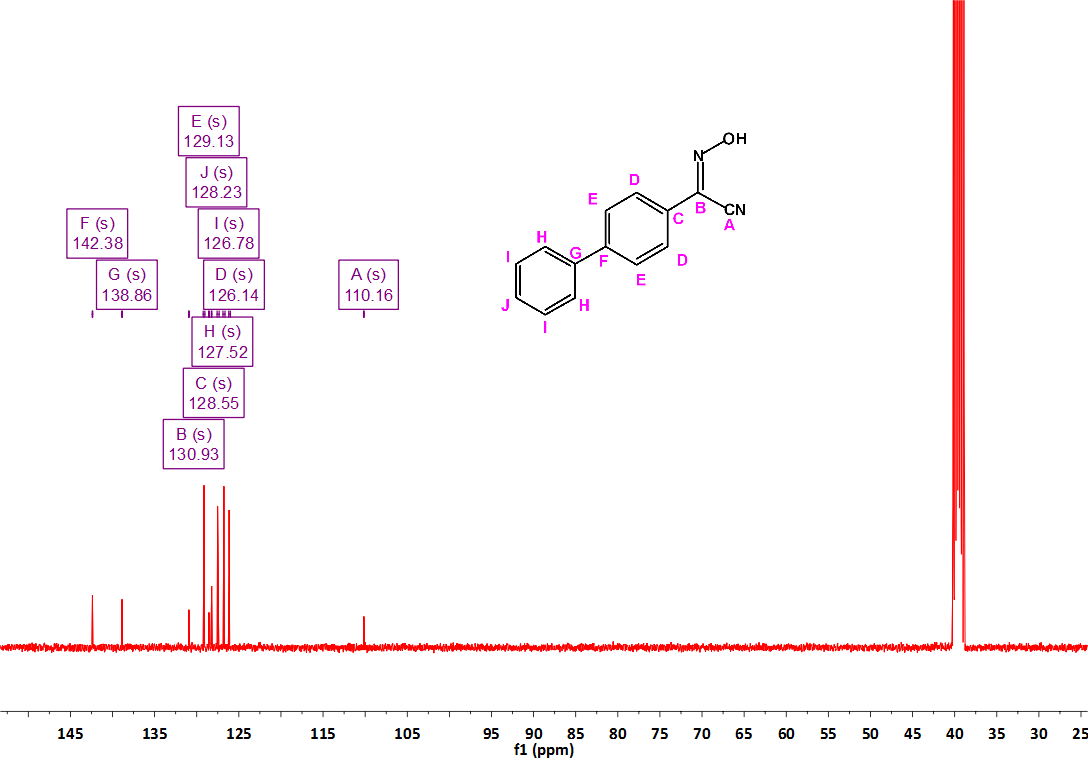
**

**2-(hydroxyimino)-2-(4-bromo)-phenylacetonitrile (2g)**

**
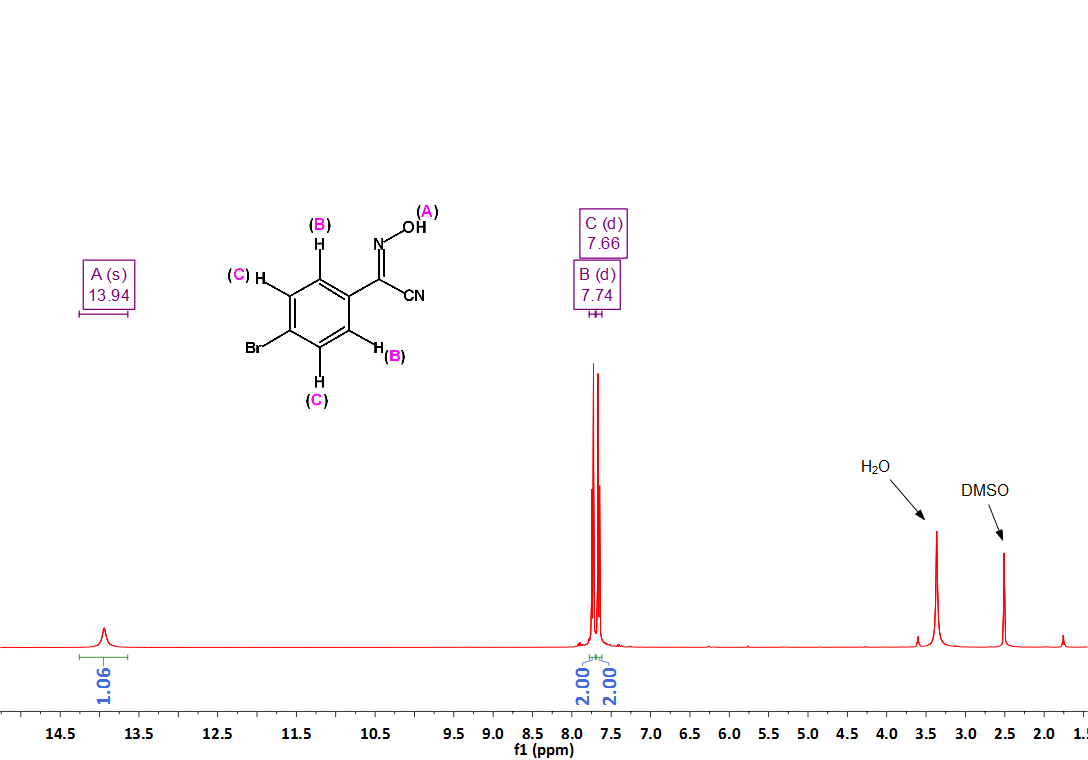
**

**
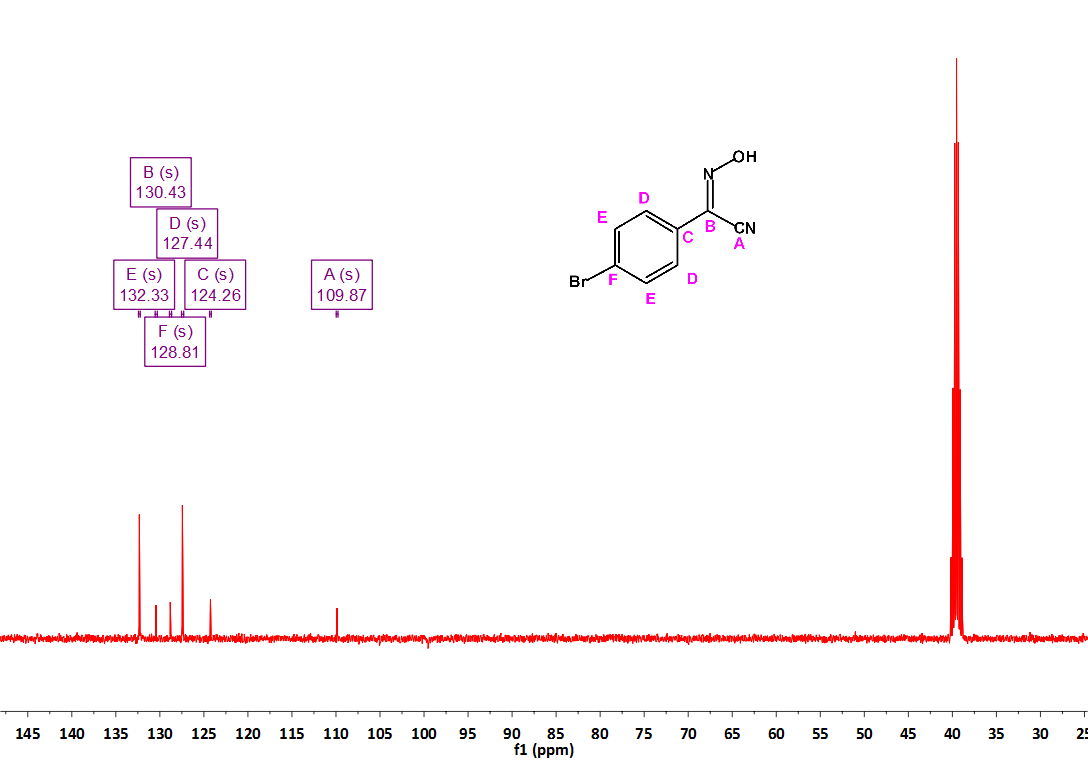
**

**2-(hydroxyimino)-2-(4-chloro)-phenylacetonitrile (2h)**

**
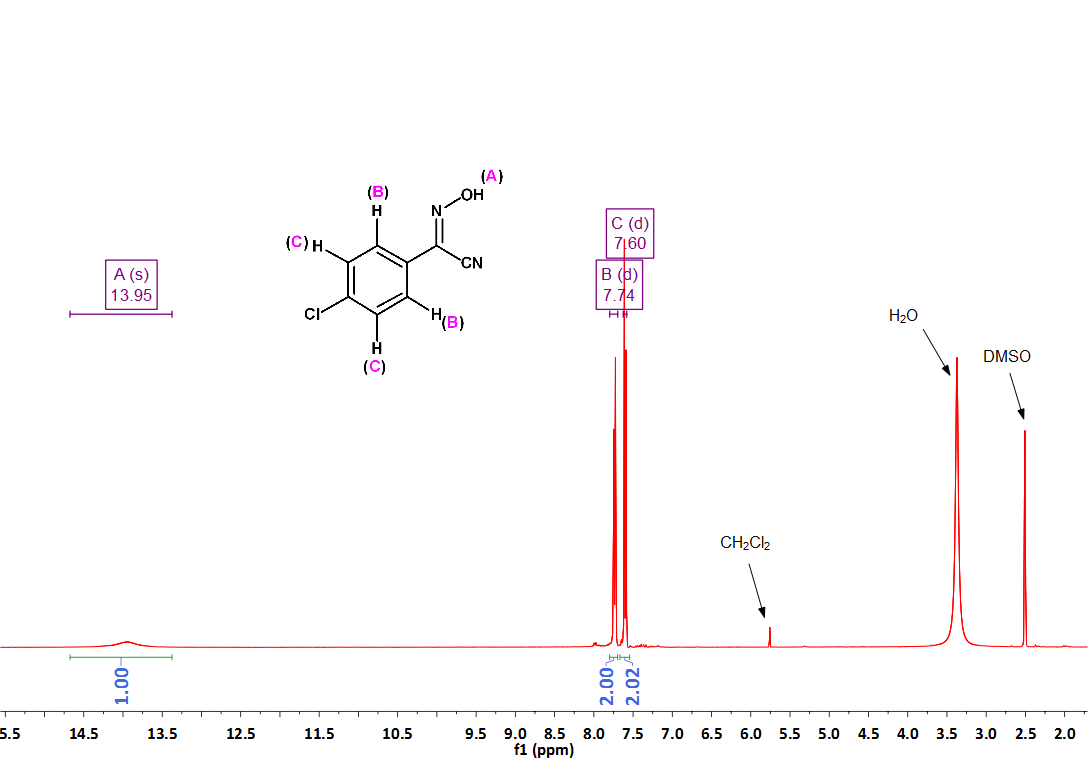
**

**
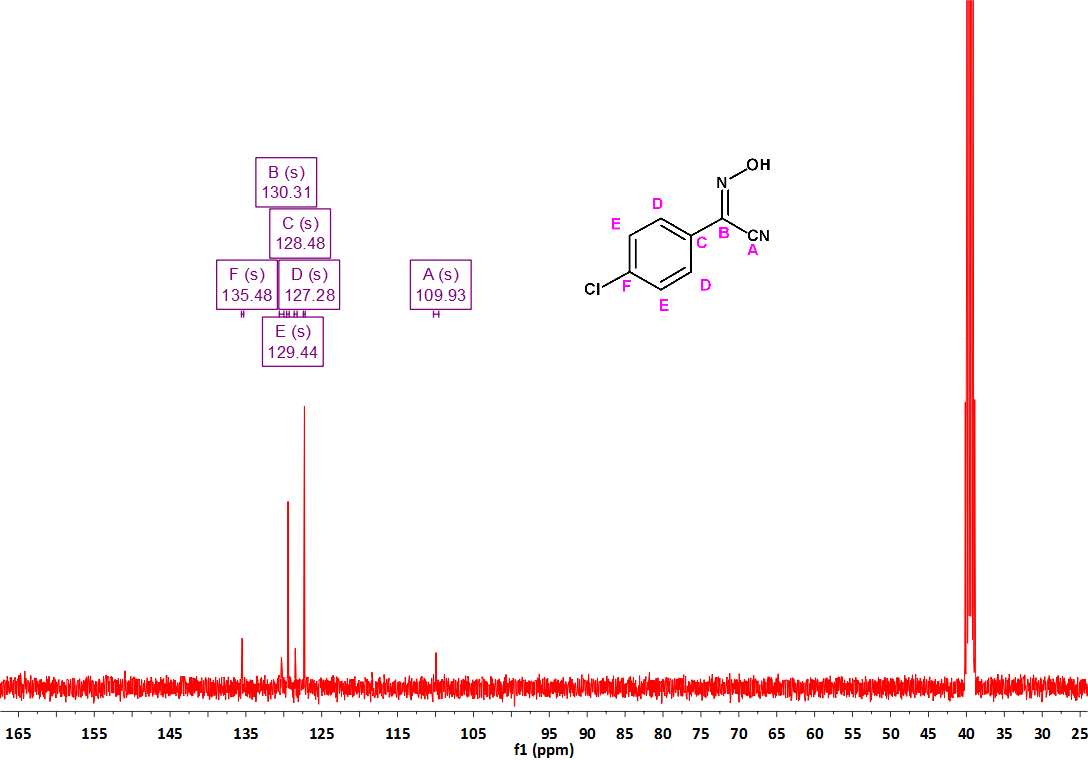
**

**2-(hydroxyimino)-2-(4-fluoro)-phenylacetonitrile (2i)**

**
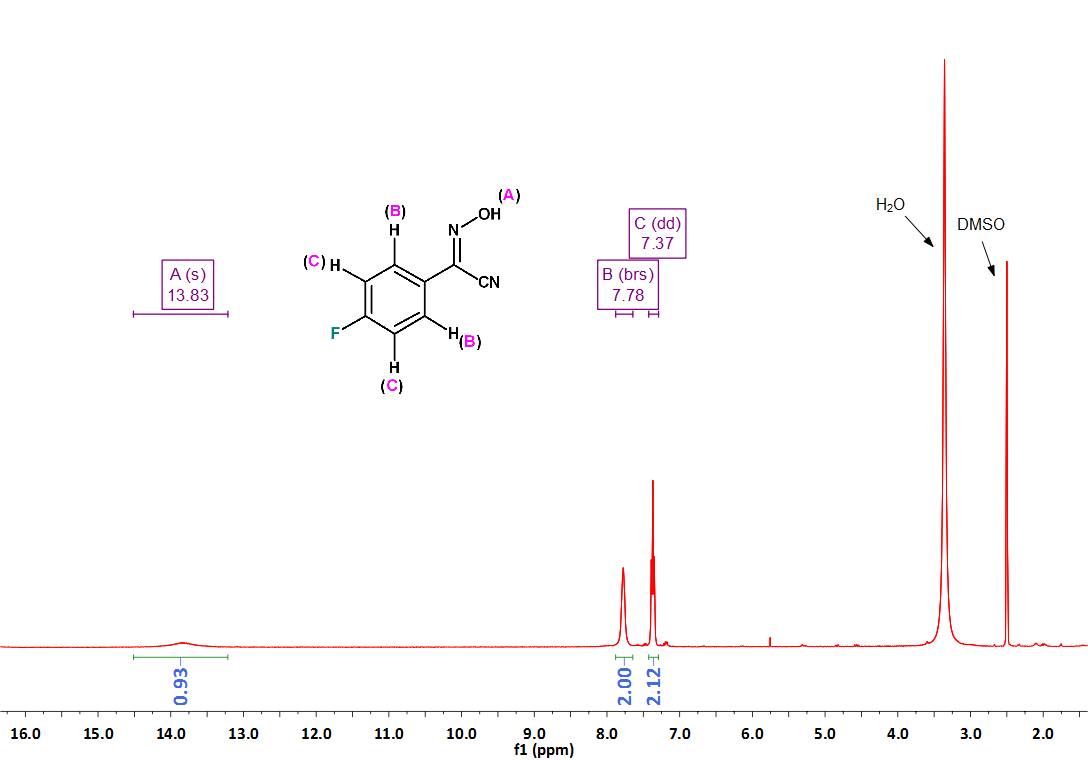
**

**
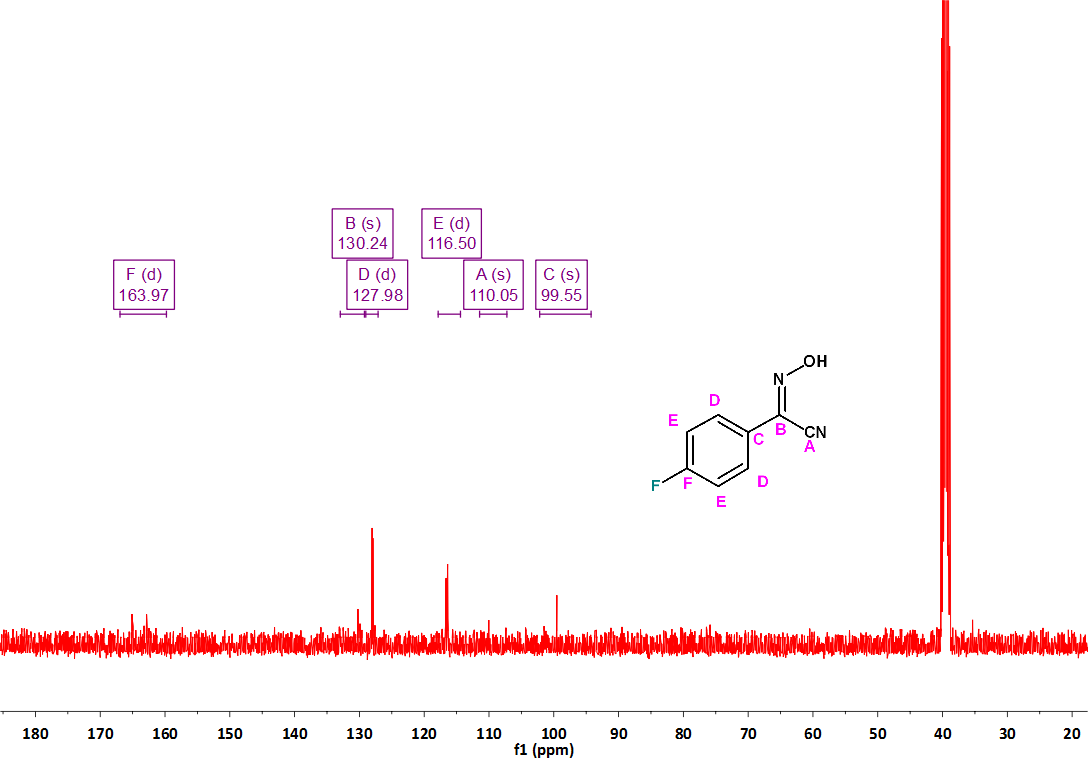
**

**2-(hydroxyimino)-2-(3-fluoro)-phenylacetonitrile (2j)**

**
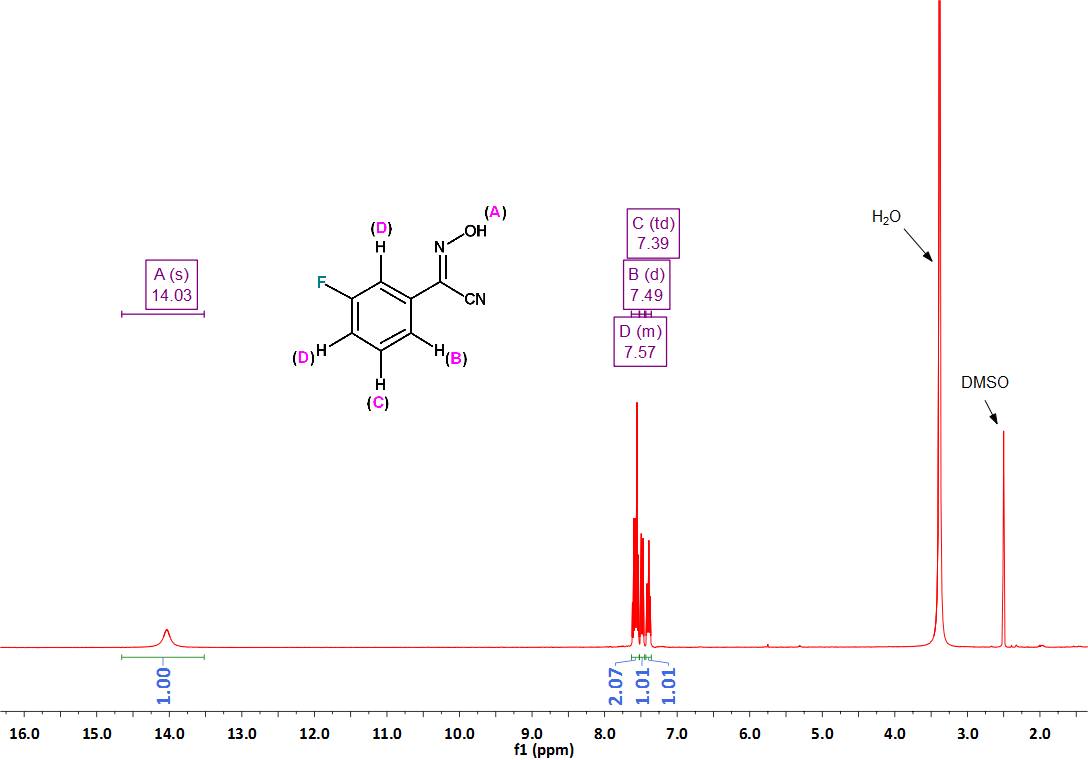
**

**
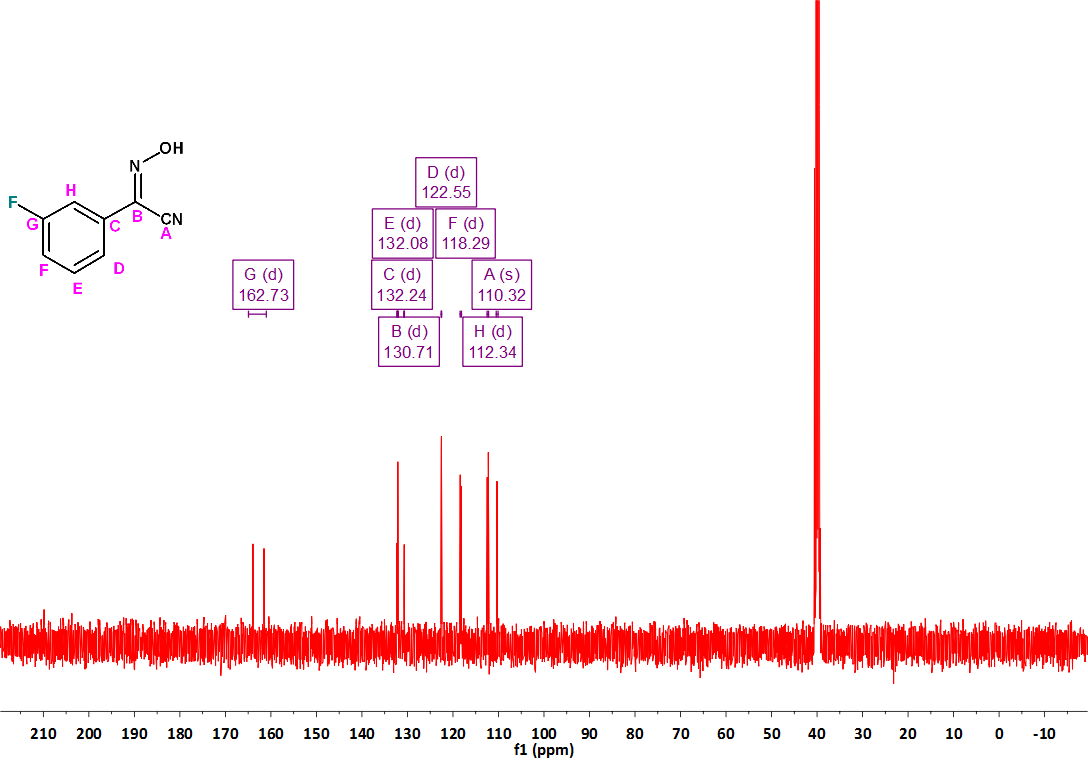
**

**2-(hydroxyimino)-2-(2-fluoro)-phenylacetonitrile (2k)**

**
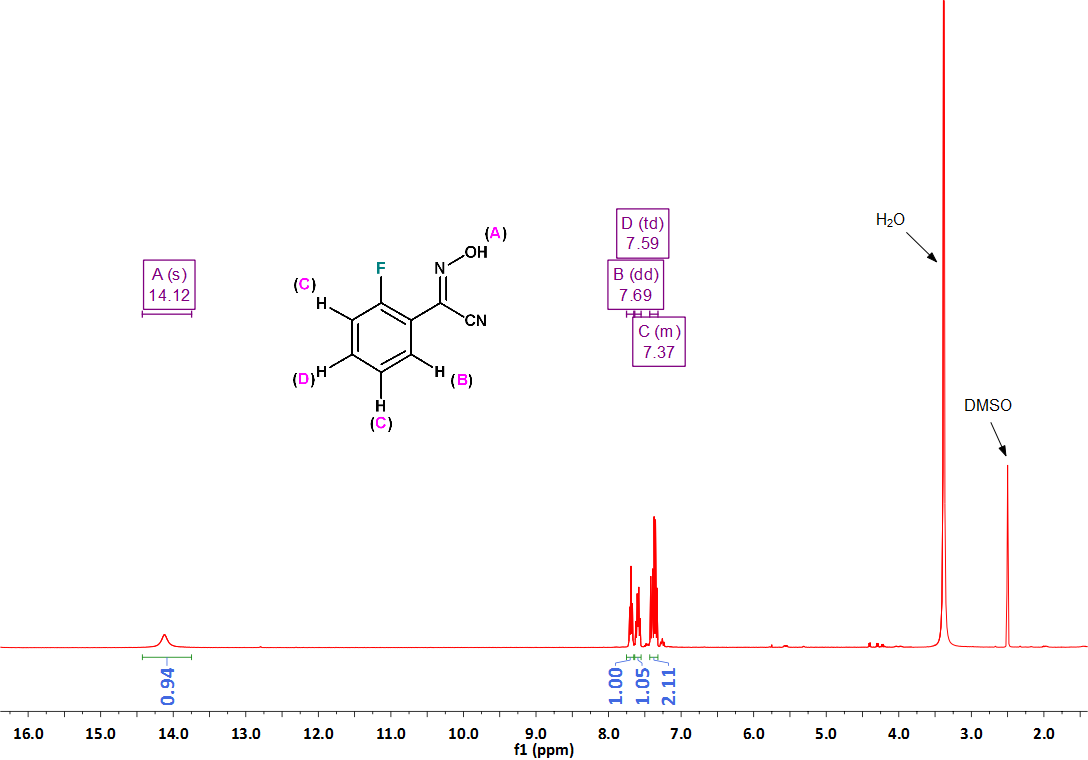
**

**
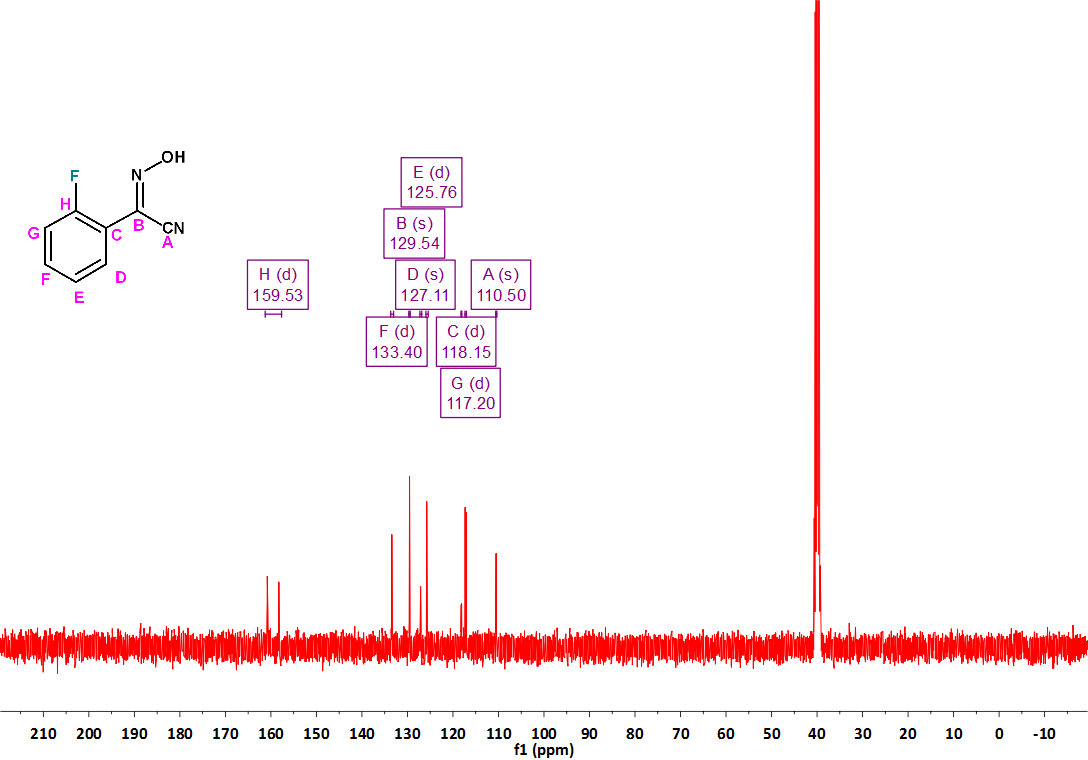
**

**2-(hydroxyimino)-2-(4-trifluoromethyl)-phenylacetonitrile (2l)**

**
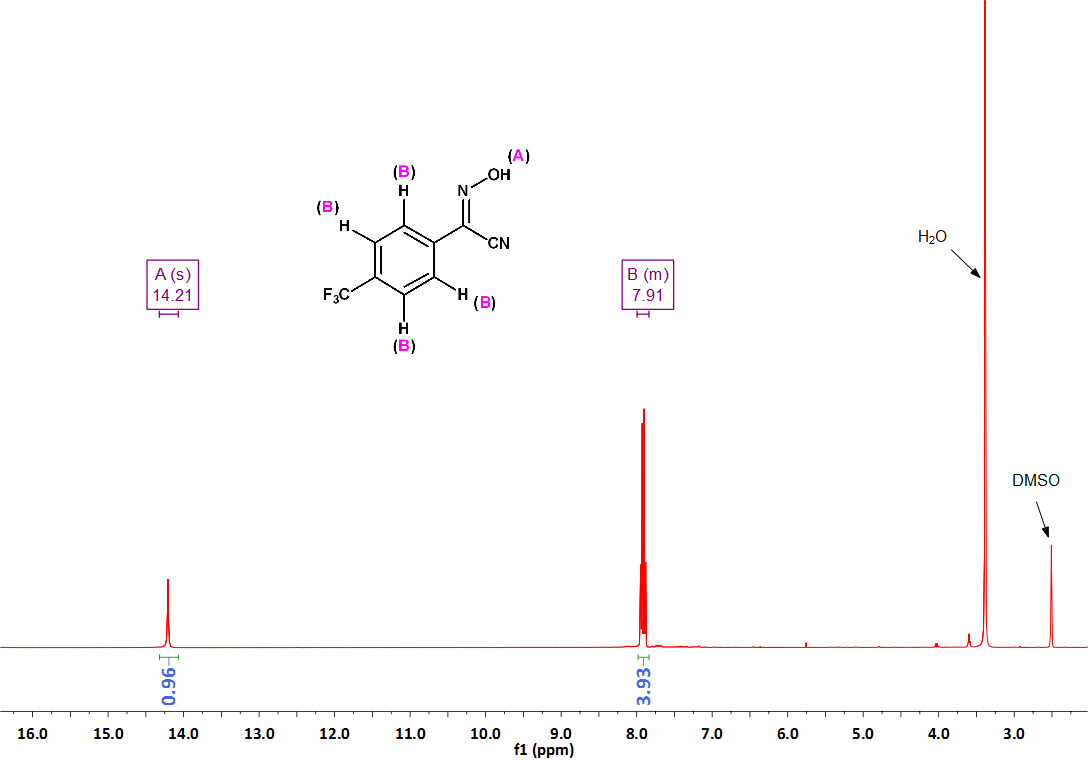
**

**
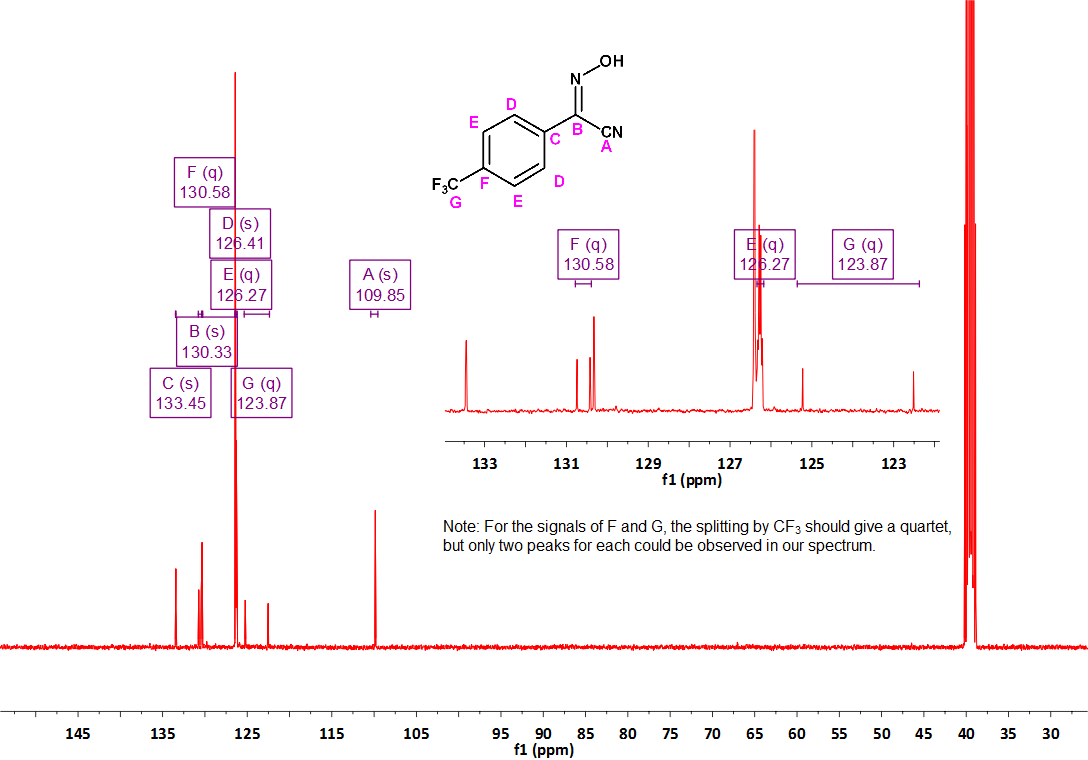
**

**2-(hydroxyimino)-2-(2,5-dimethyl)-phenylacetonitrile (2m)**

**
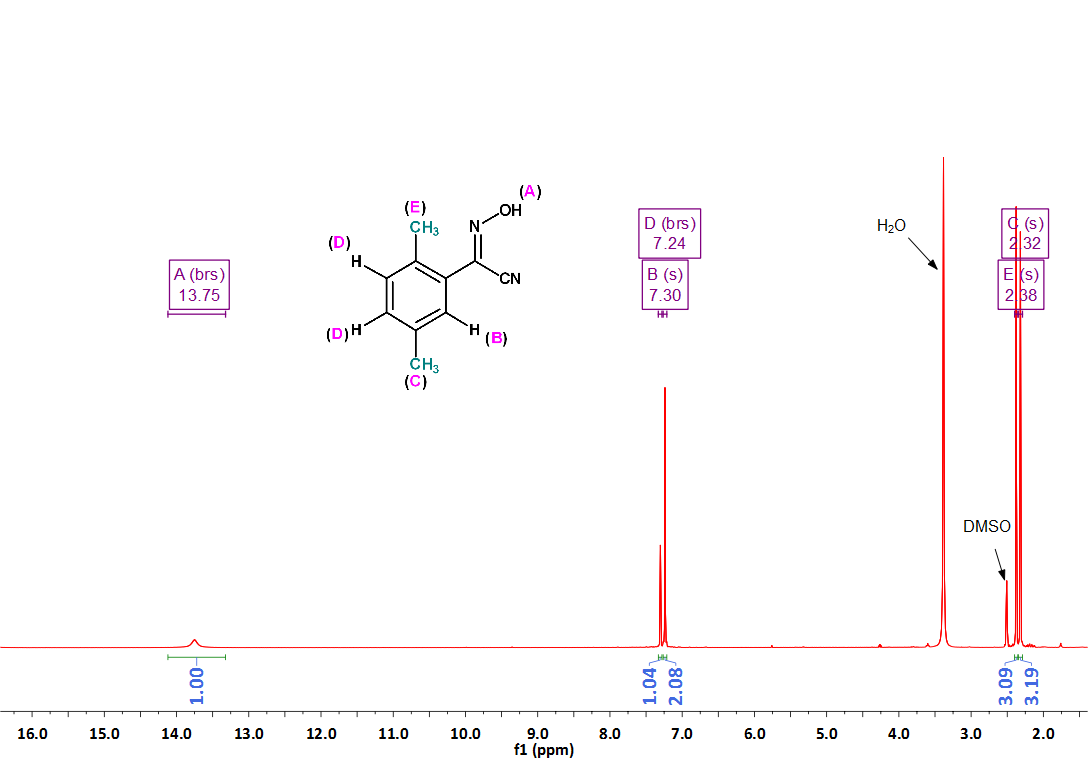
**

**
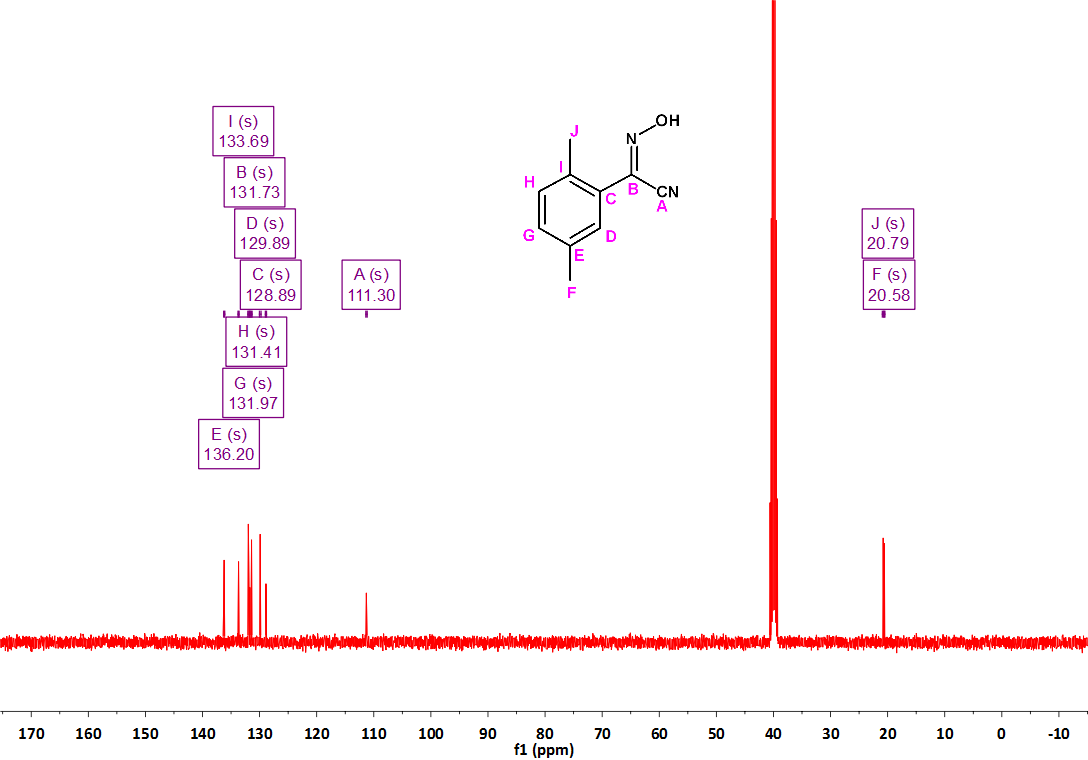
**

**2-(hydroxyimino)-2-(3,5-dimethyl)-phenylacetonitrile (2n)**

**
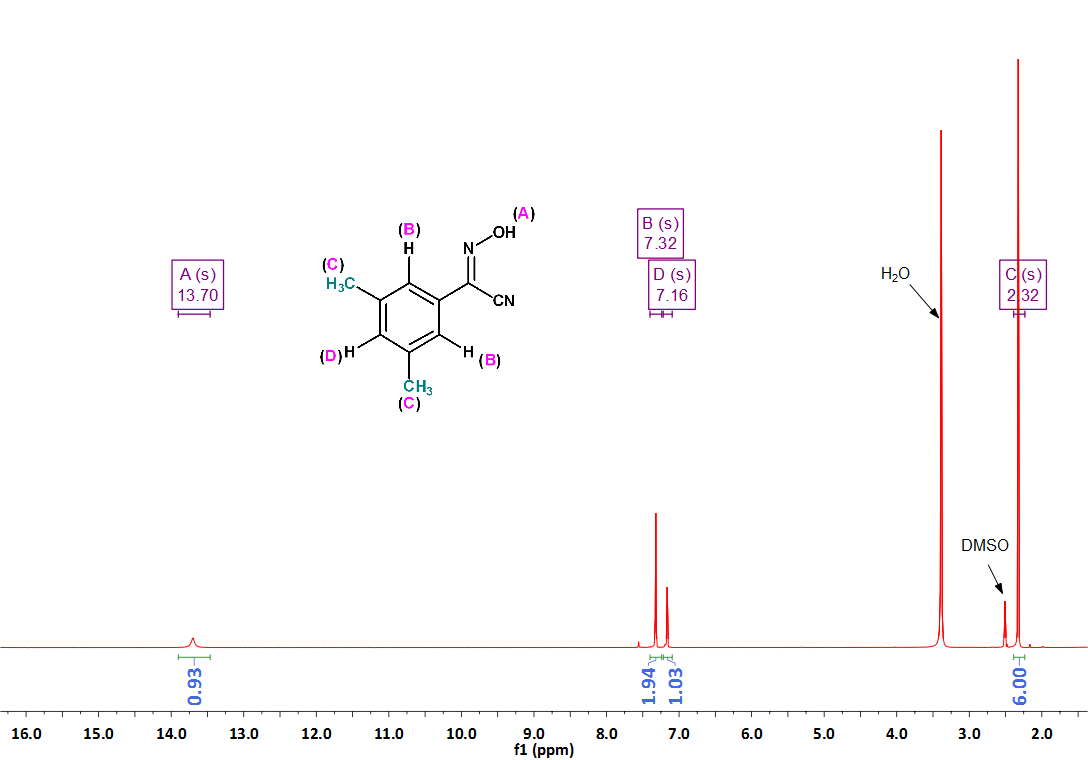
**

**
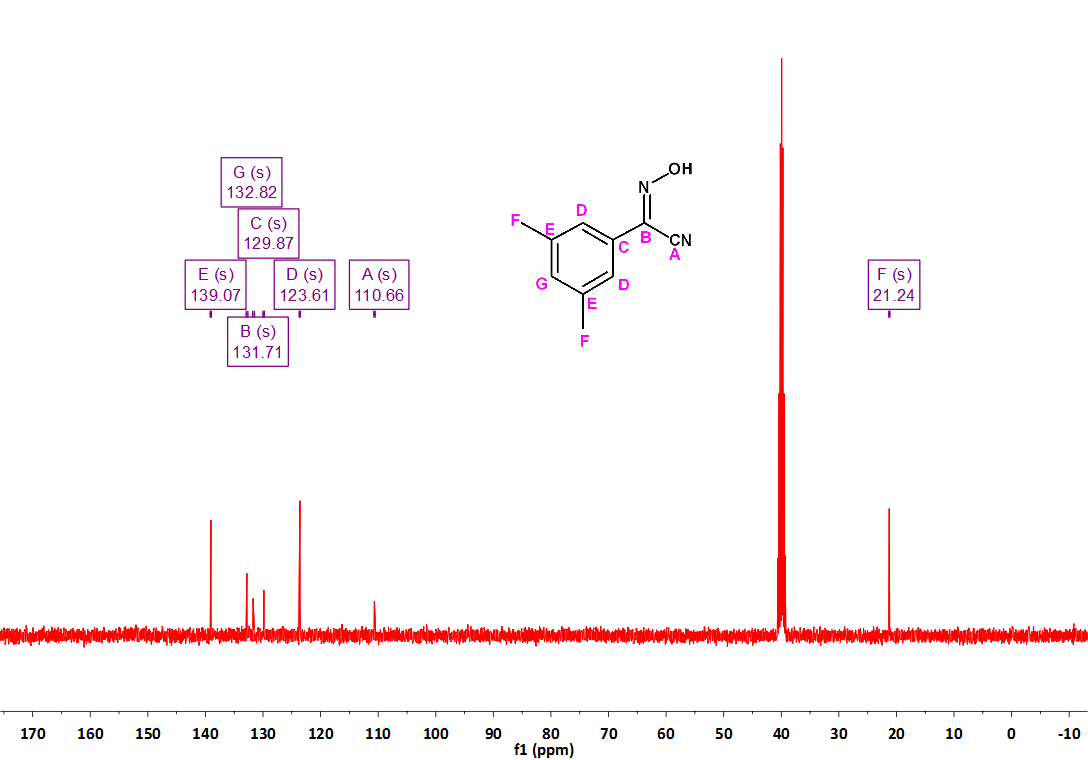
**

**2-(hydroxyimino)-2-(3,4-dimethoxyl)-phenylacetonitrile (2o)**

**
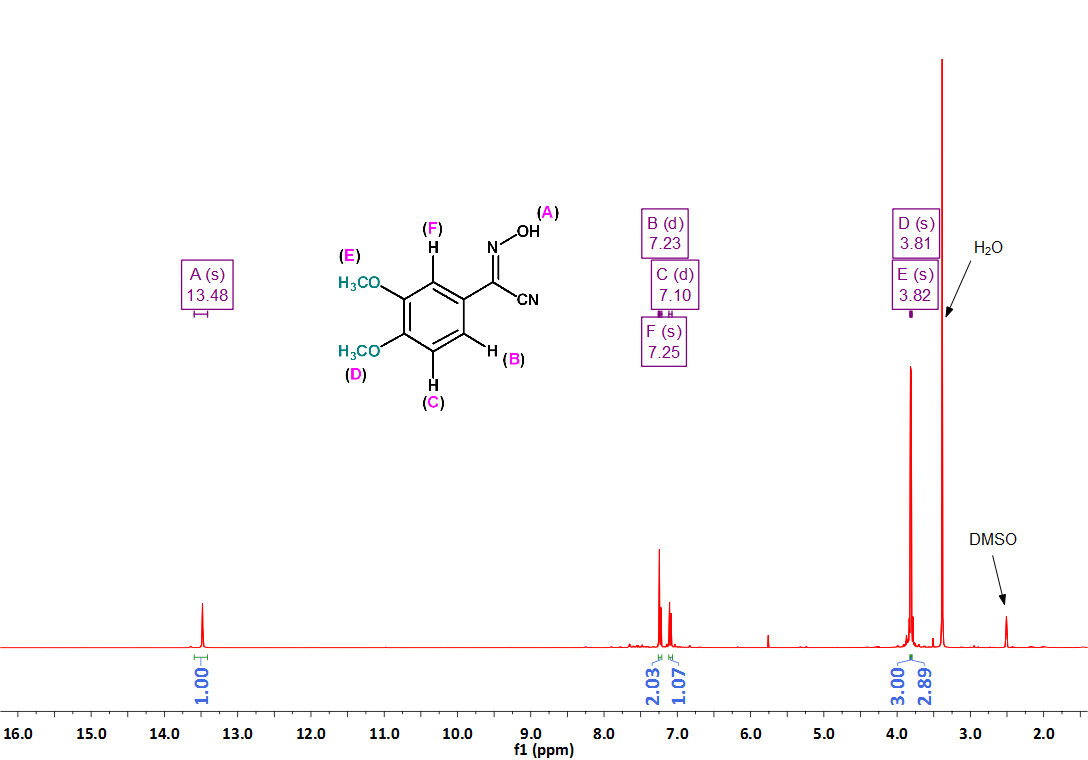
**

**
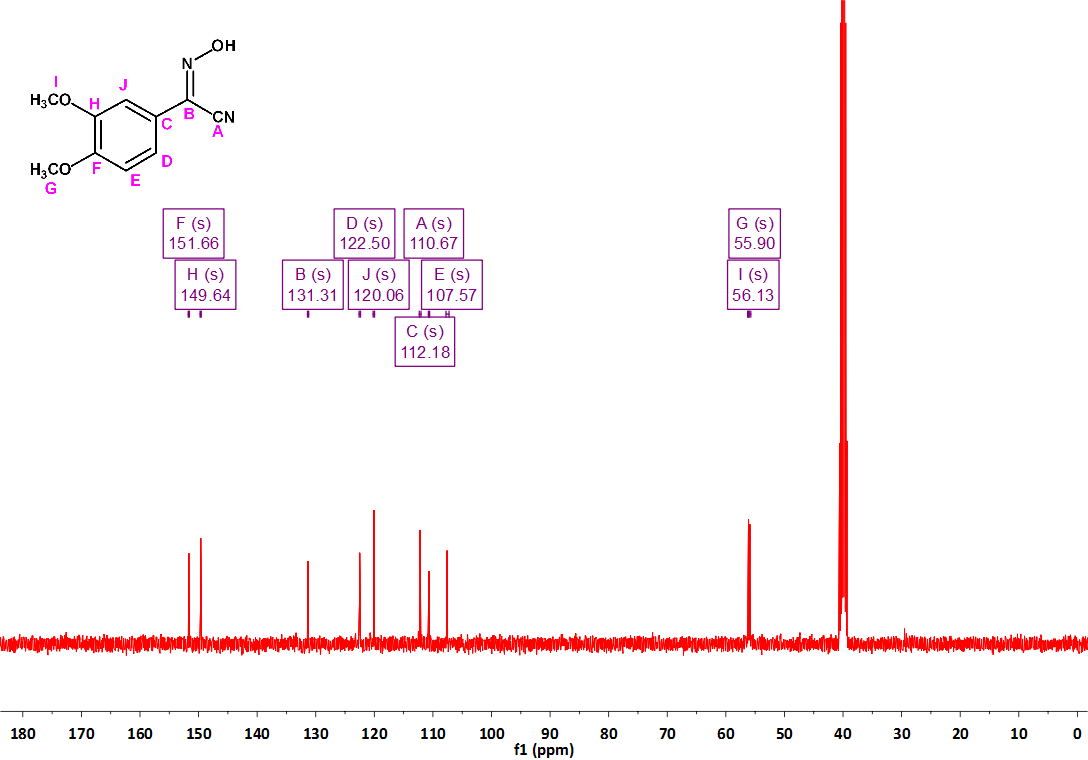
**

**N-hydroxybenzo[d][1,3]dioxole-5-carbimidoyl cyanide (2p)**

**
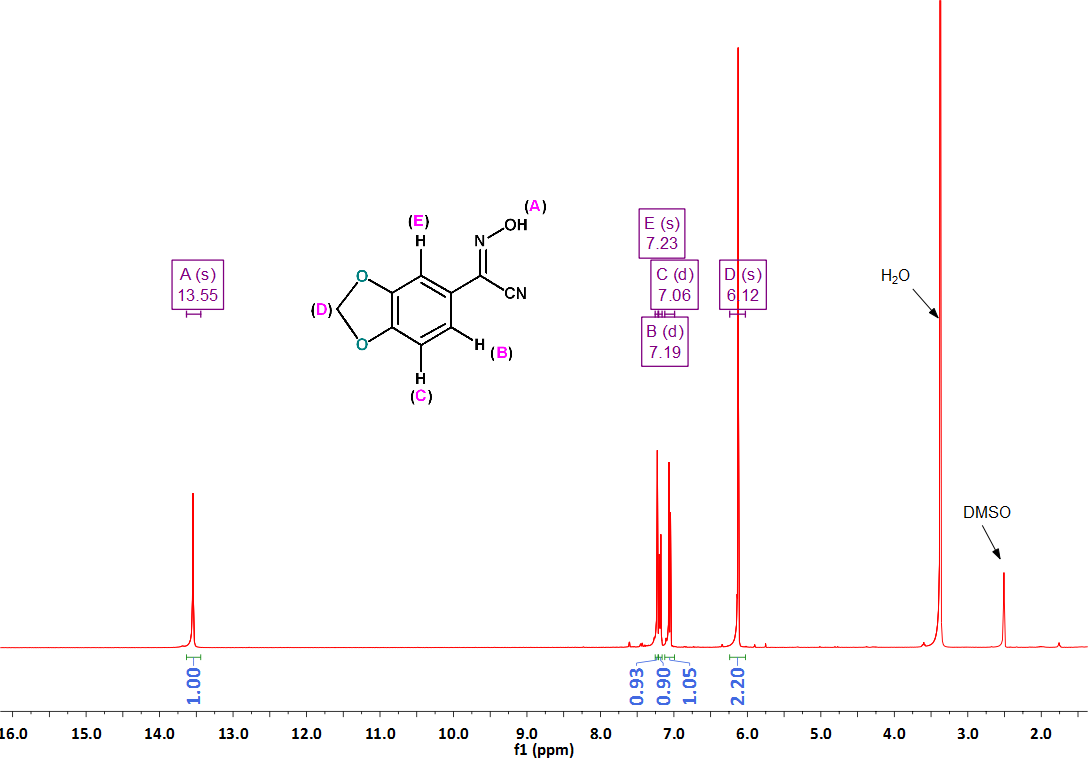
**

**
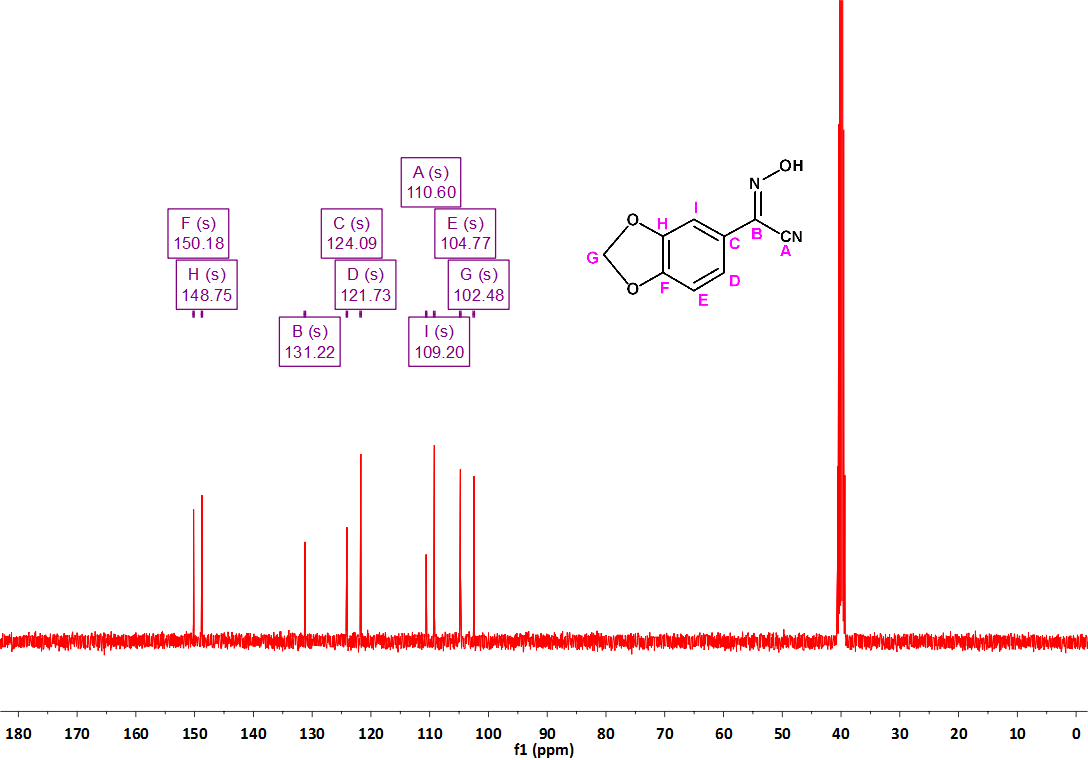
**

**2,4-Di-fluoro-N-hydroxybenzimidoyl cyanide (2q)**

**
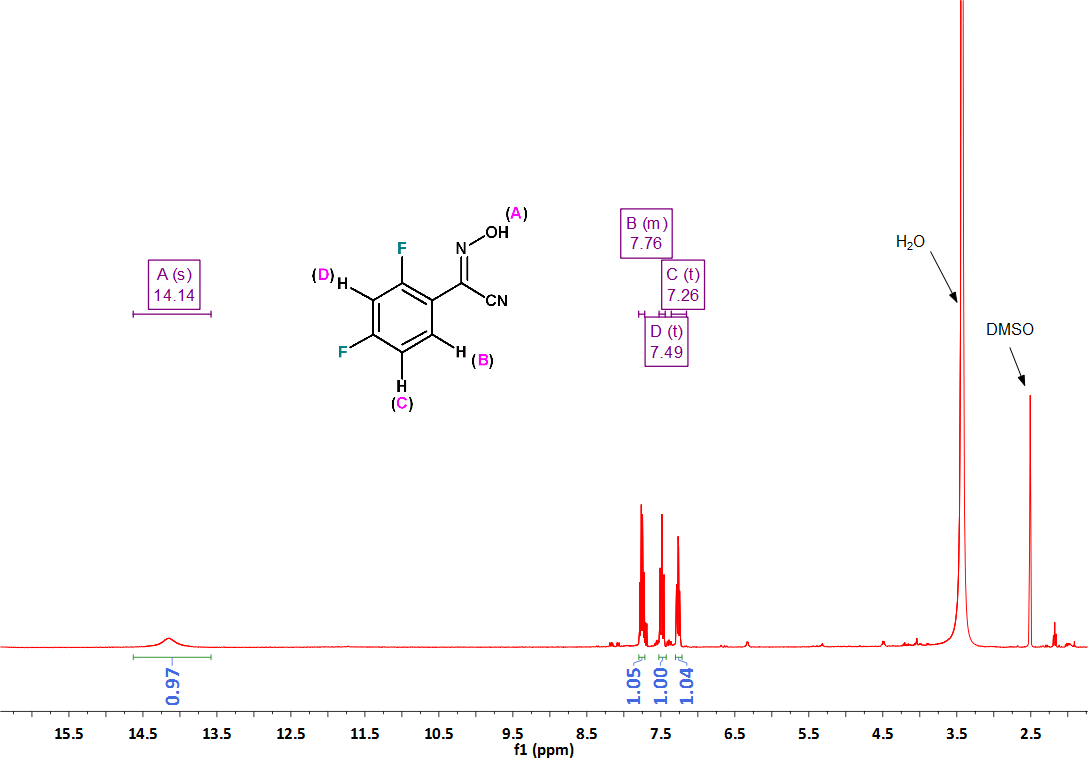
**

**
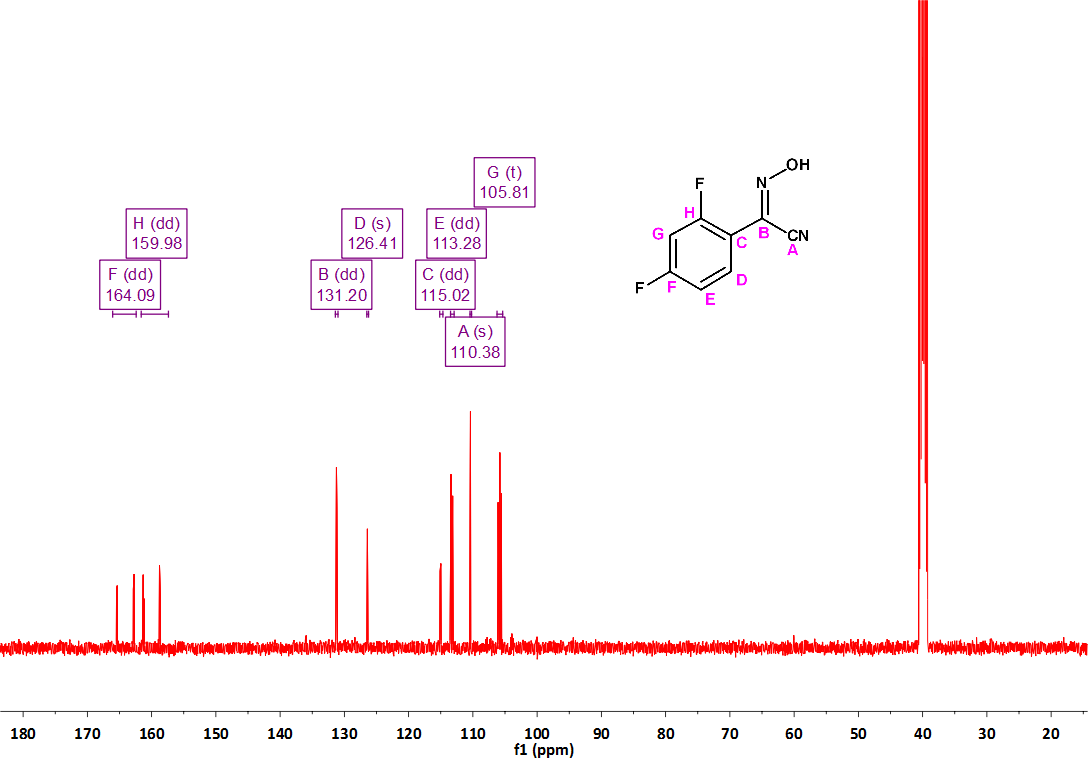
**

**2-(hydroxyimino)-2-naphthylacetonitrile (2r)**

**
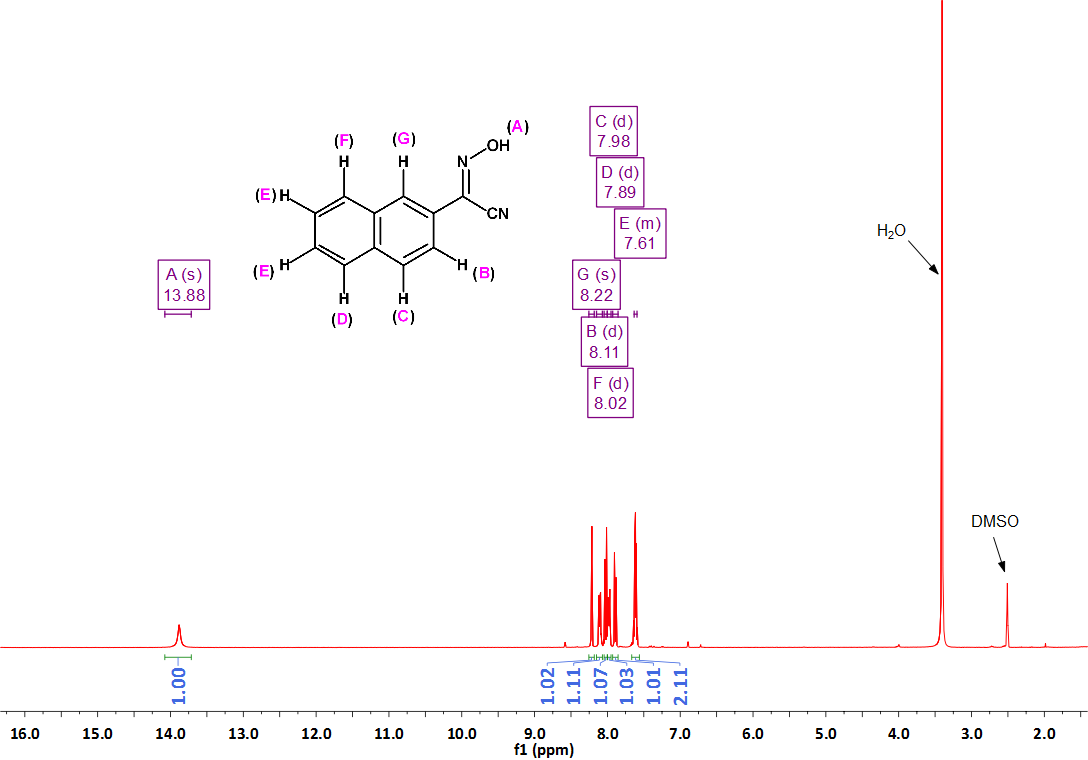
**

**
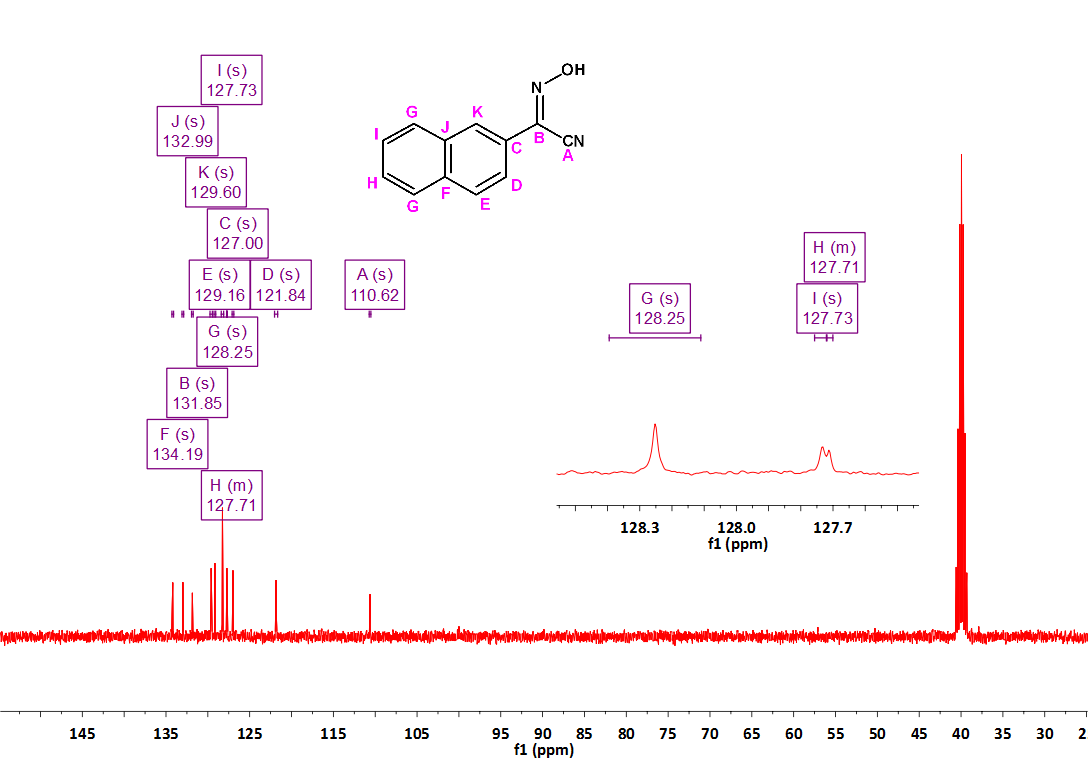
**

**Hydroxy-1-naphthimidoyl cyanide (2s)**

**
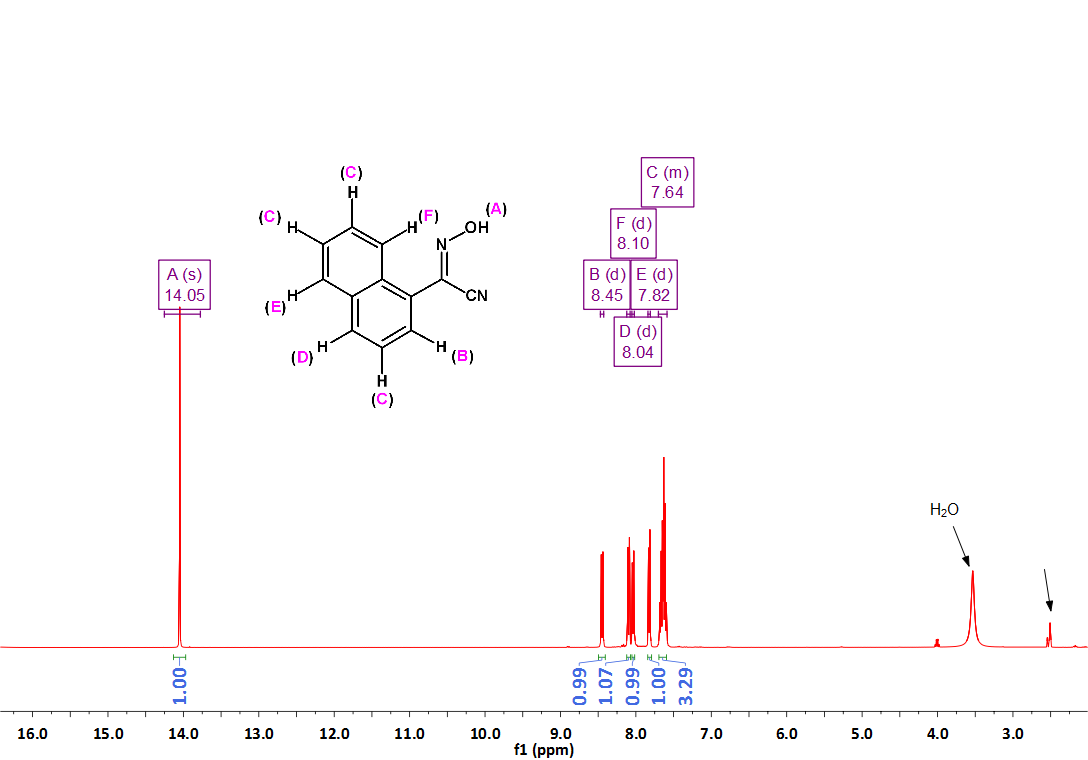
**

**
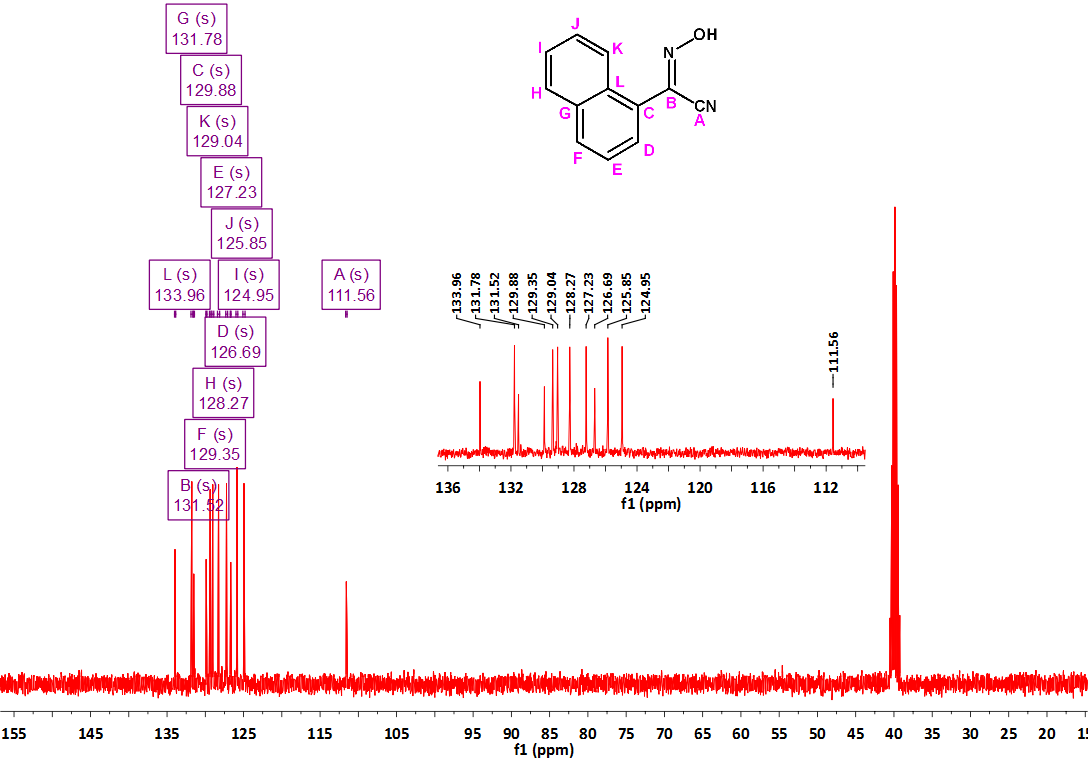
**

**N-hydroxythiophene-2-carbimidoyl cyanide (2t)**

**
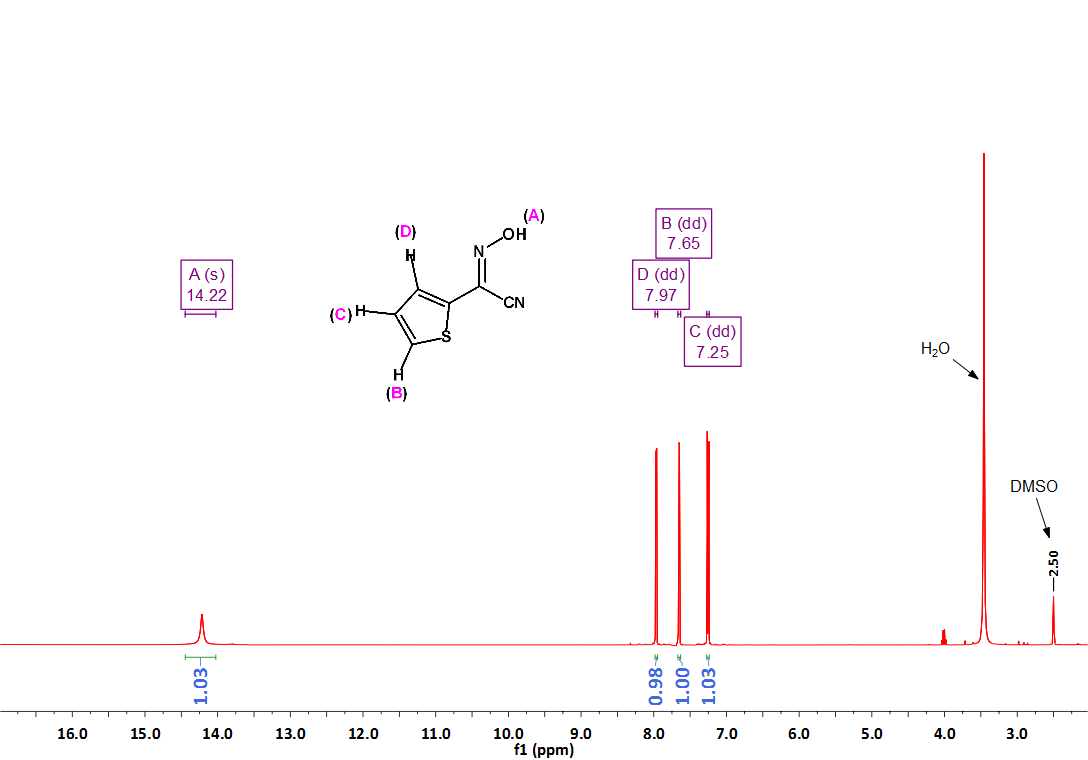
**

**
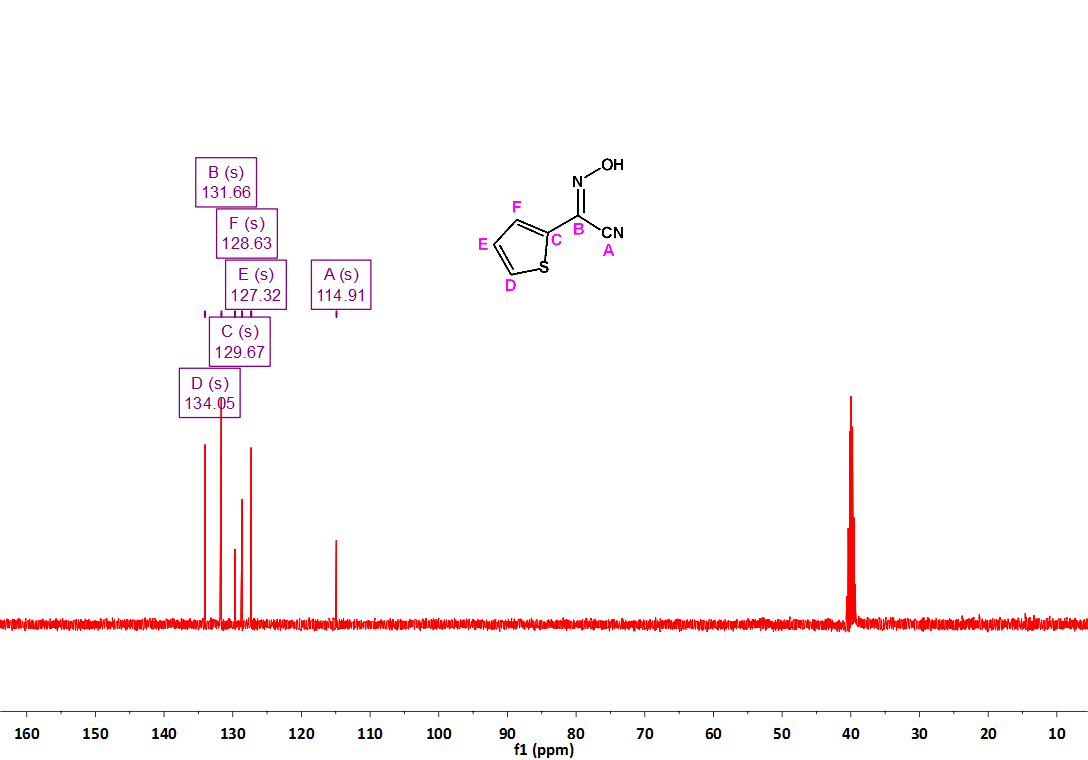
N-hydroxythiophene-3-carbimidoyl cyanide (2u)**

**
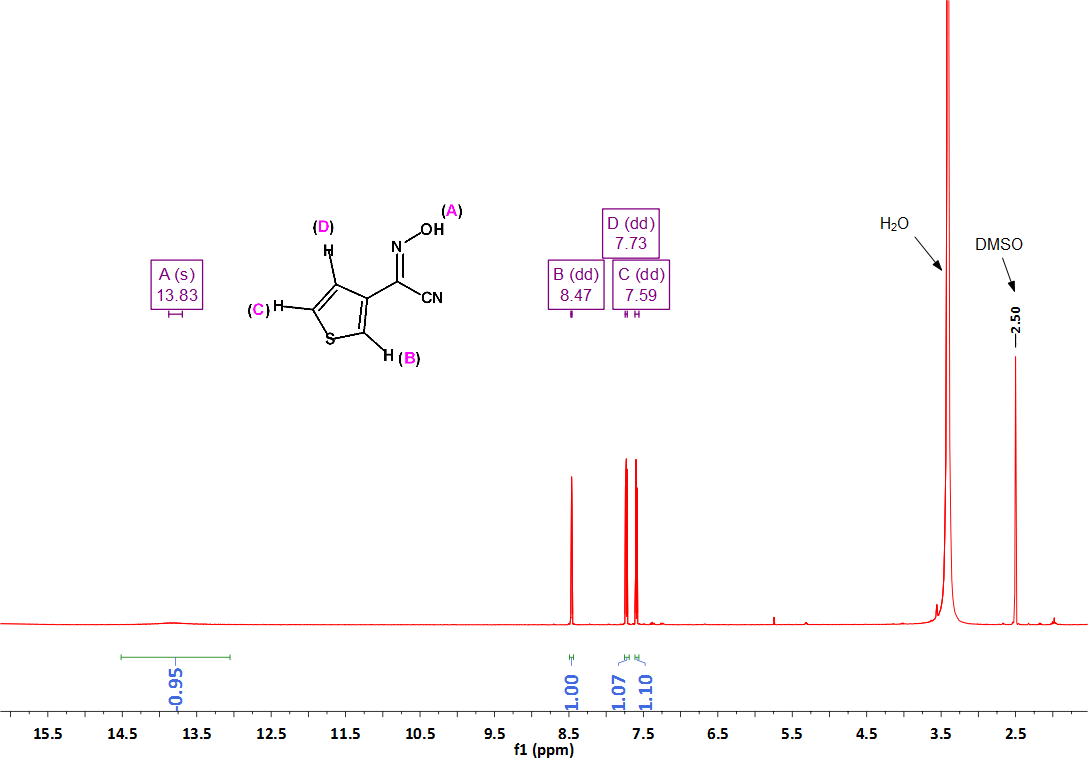
**

**
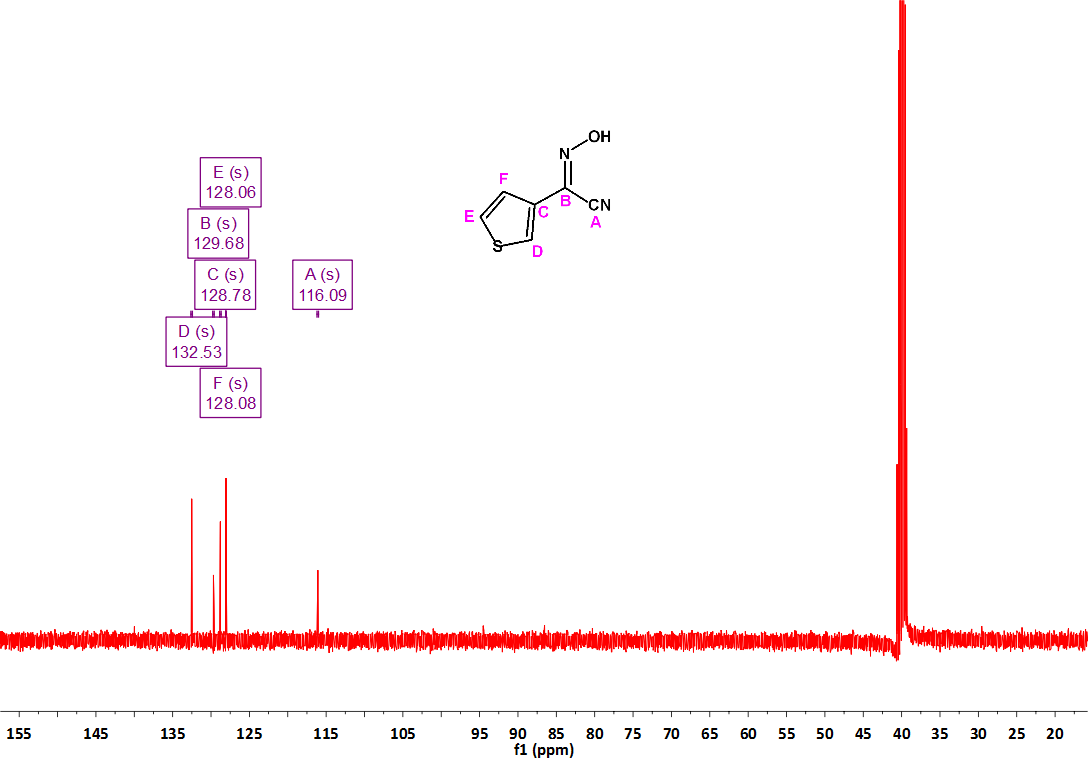
**

**Phthalimide (4a)**

**
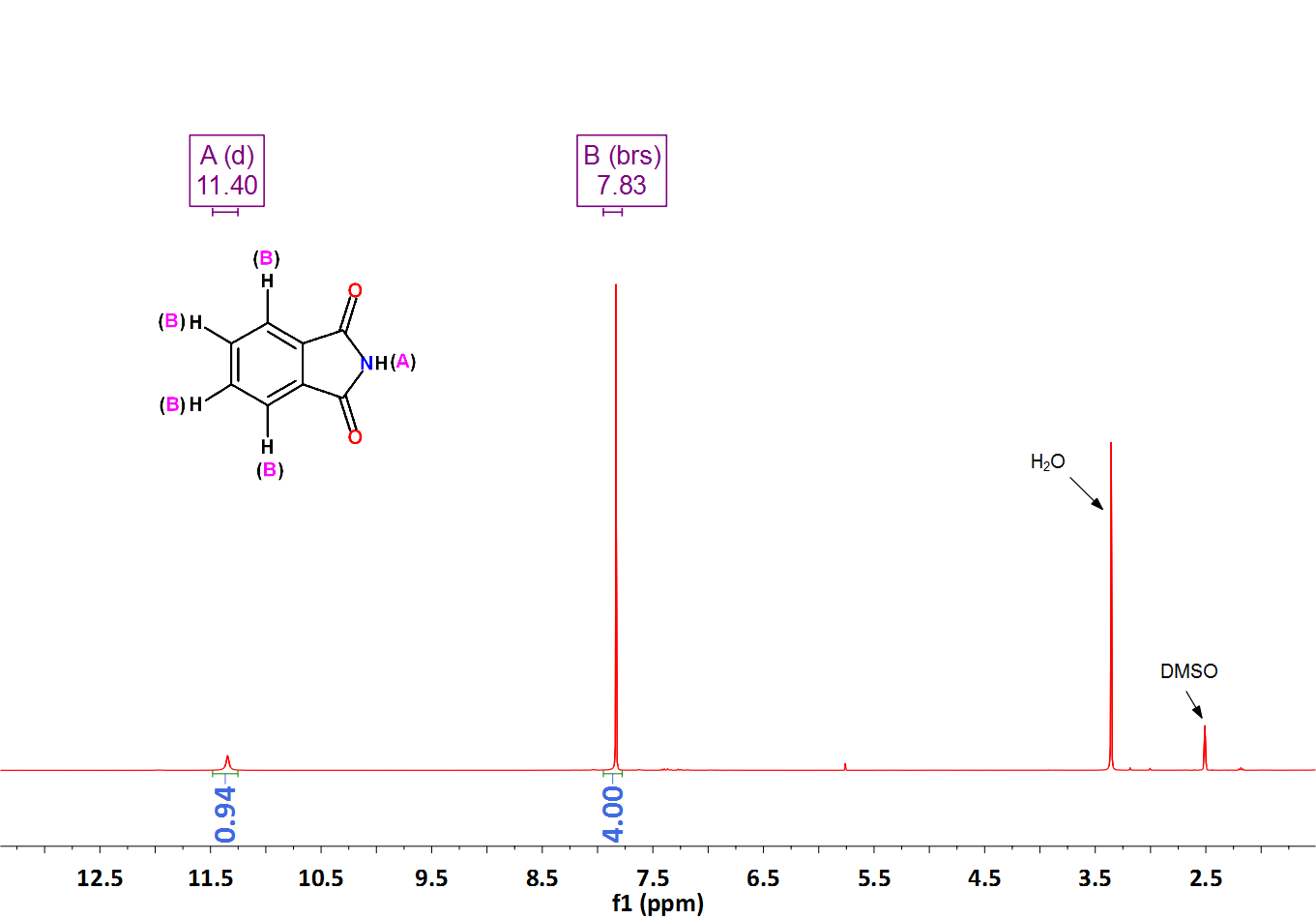
**

**
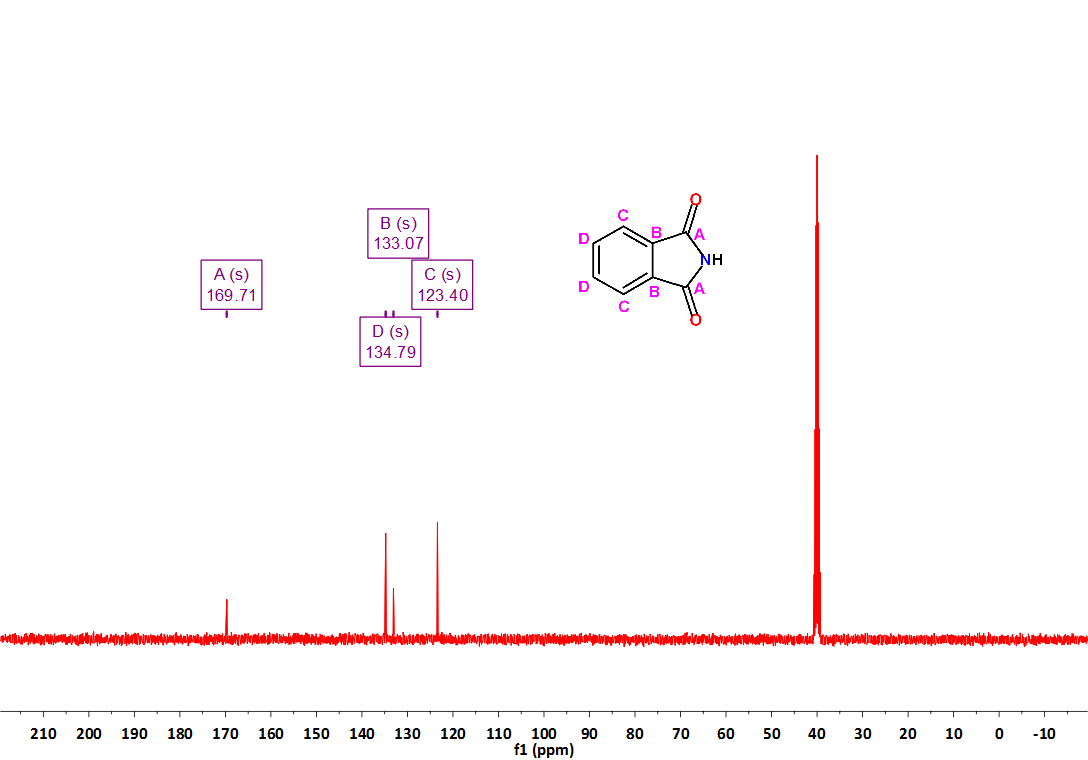
**

**3-Methyl-phthalimide (4b)**

**
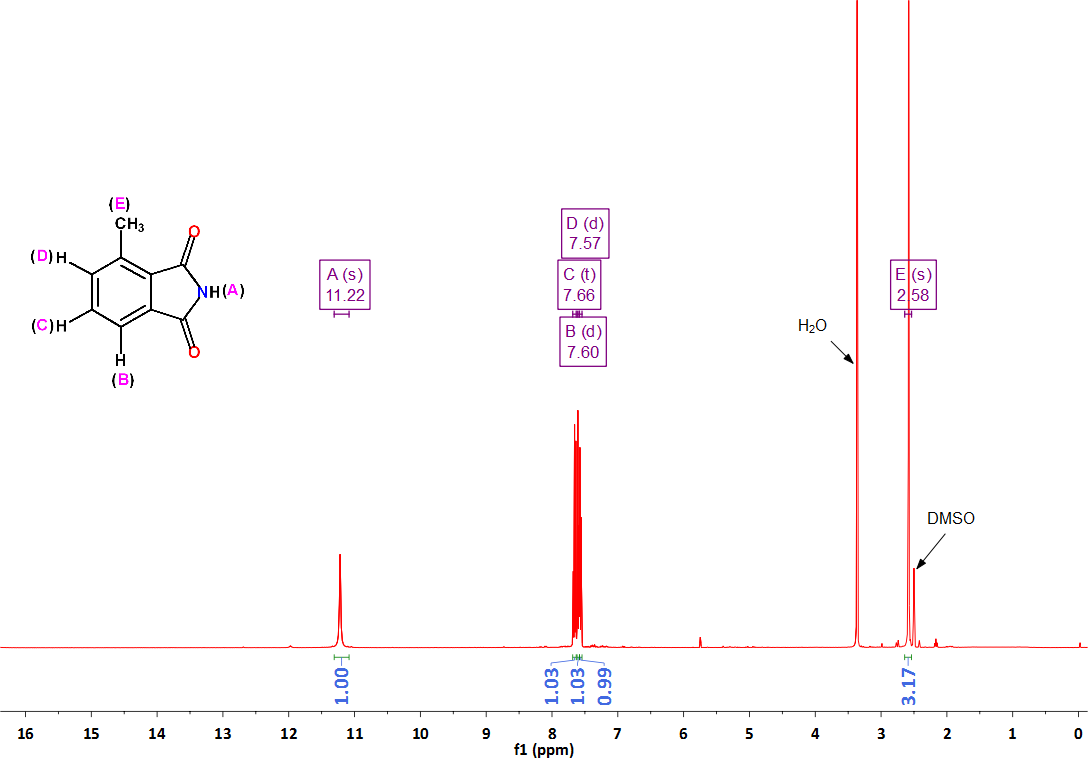
**

**
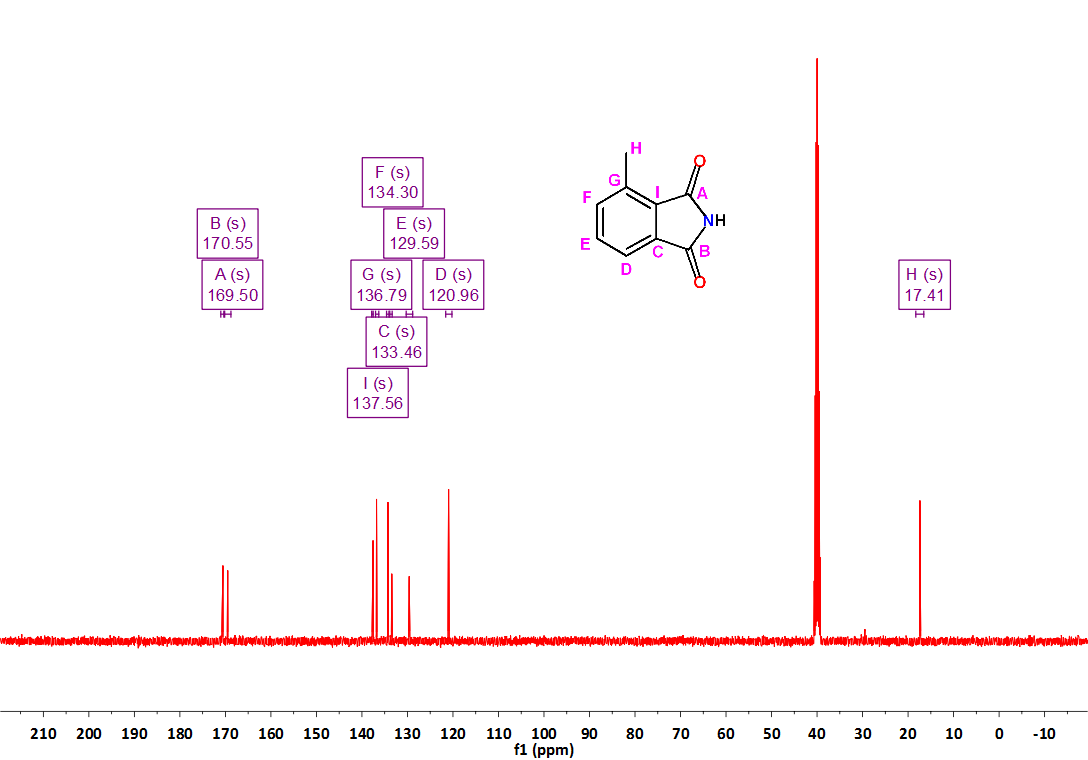
**

**4-Methylphthalimide (4c)**

**
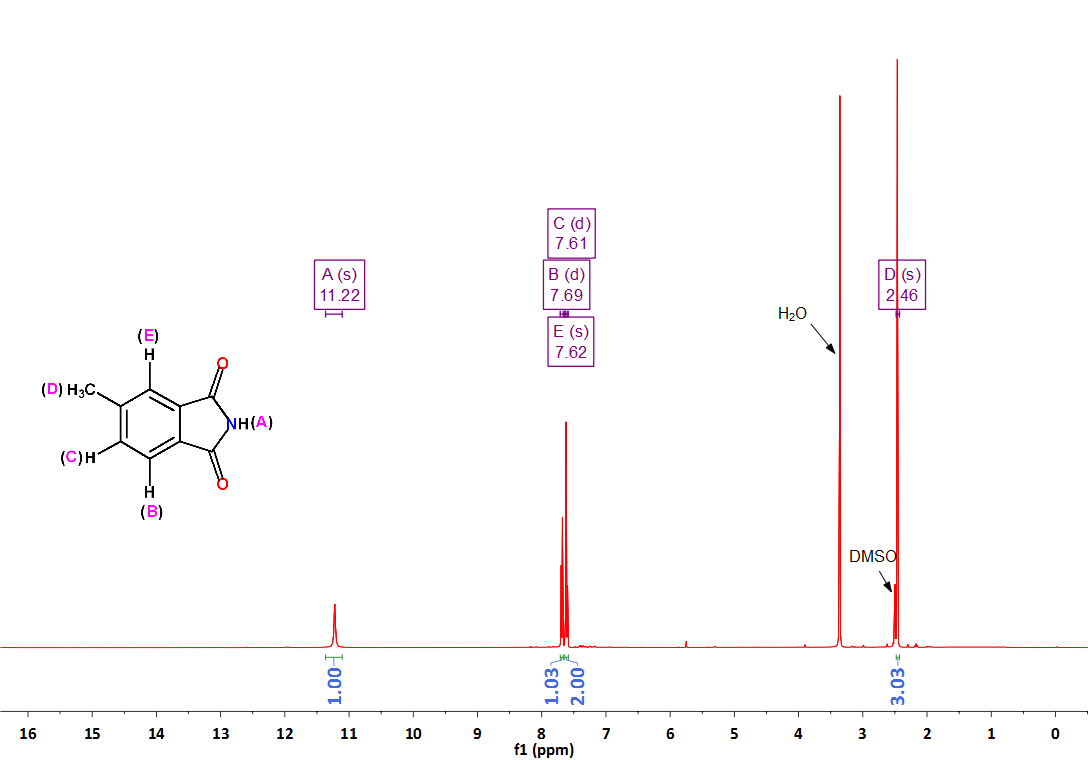
**

**
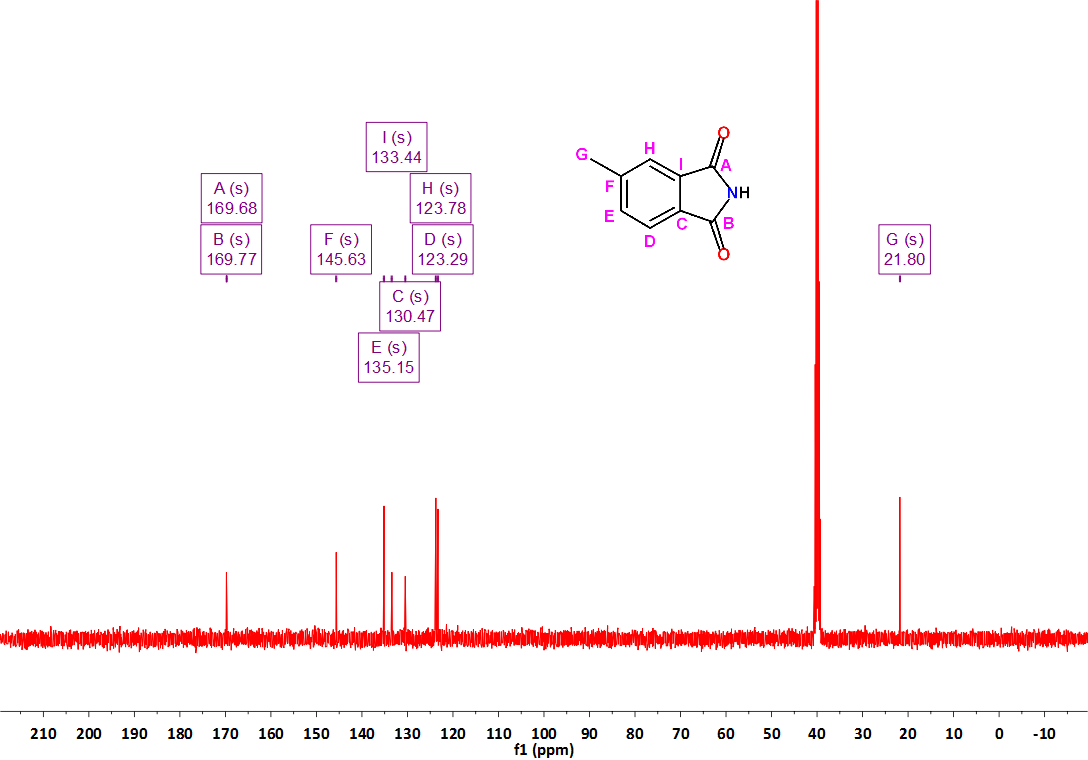
**

**4-Tert-butyl-phthalimide (4d)**

**
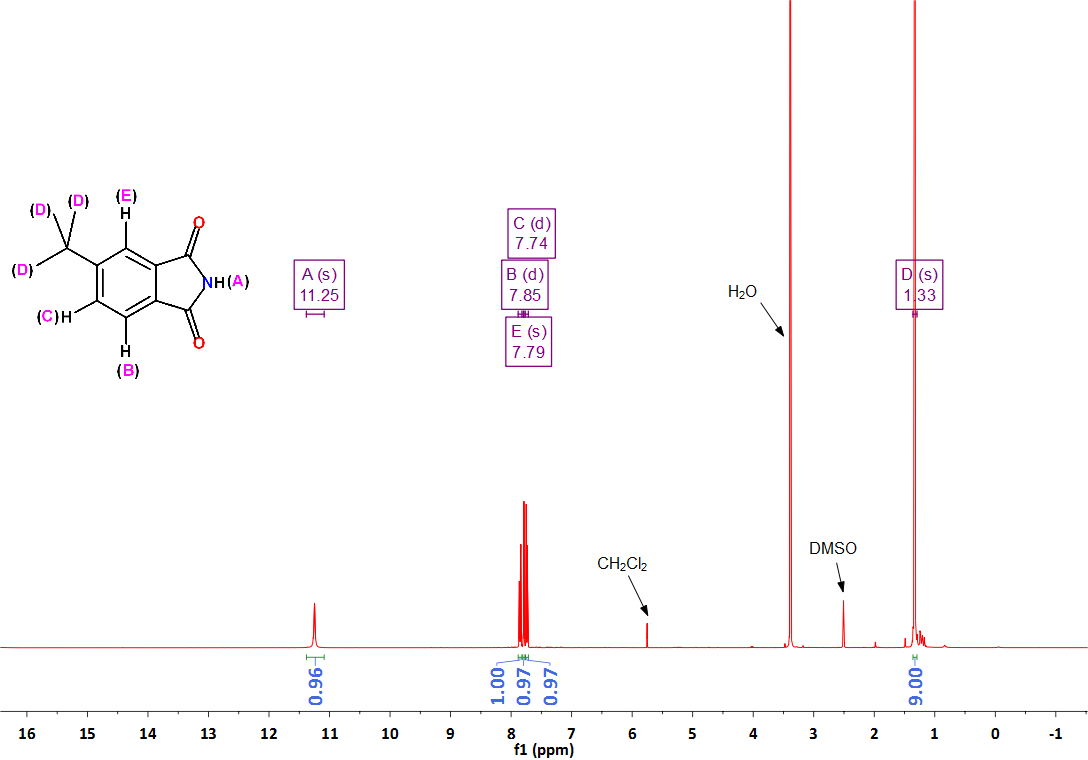
**

**
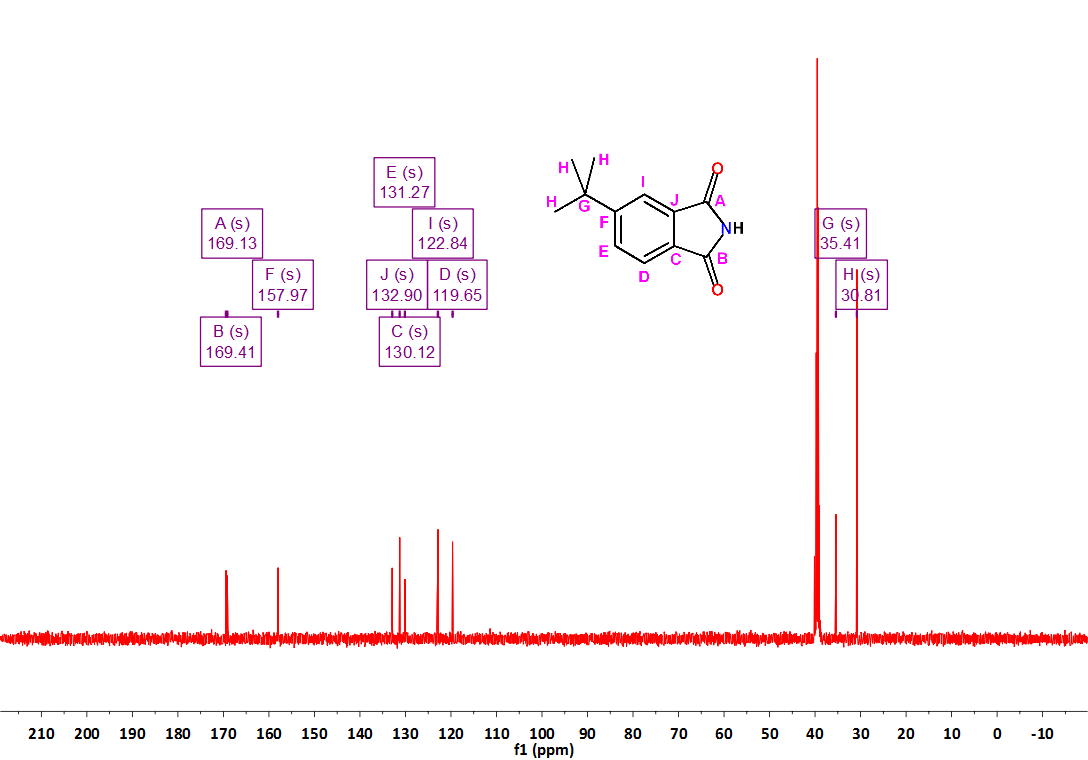
**

**4-Methoxylphthalimide (4e)**

**
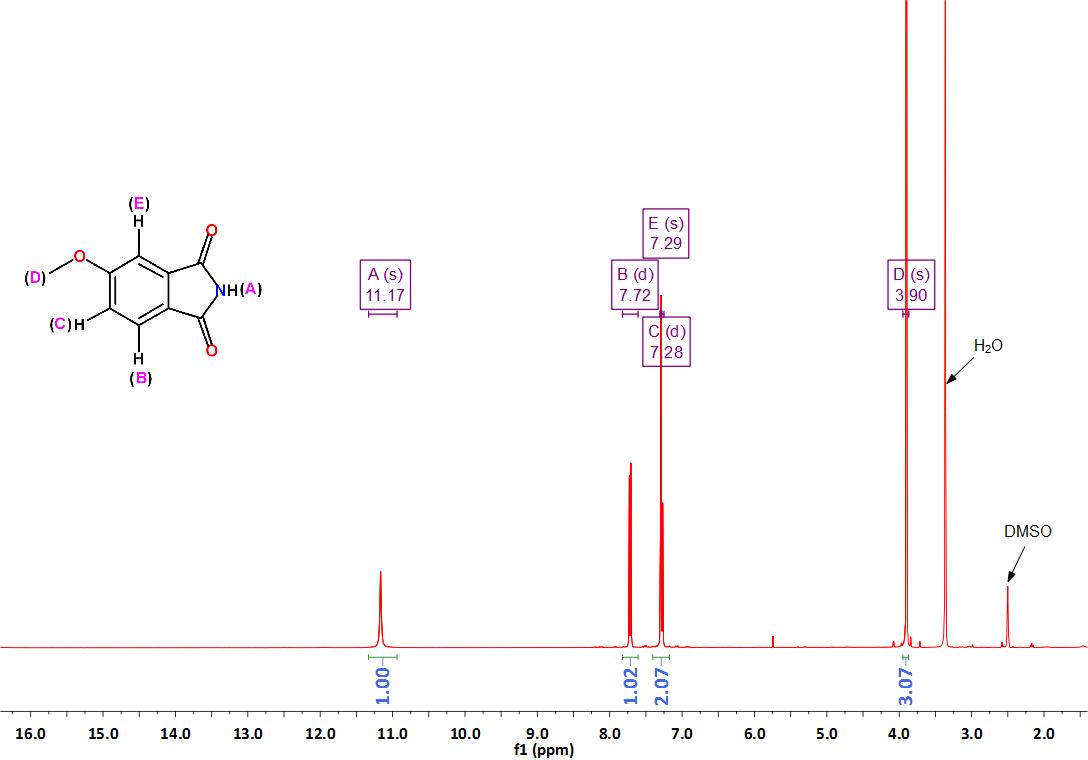
**

**
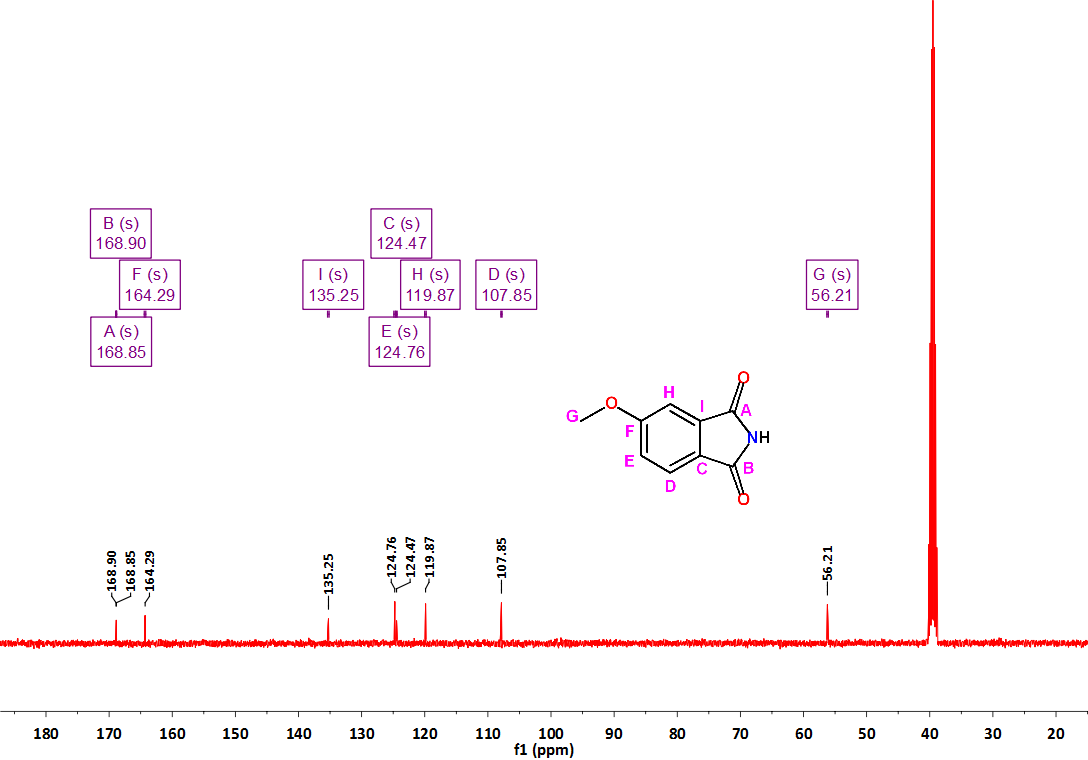
**

**4-Benzyloxy-phthalimide (4f)**

**
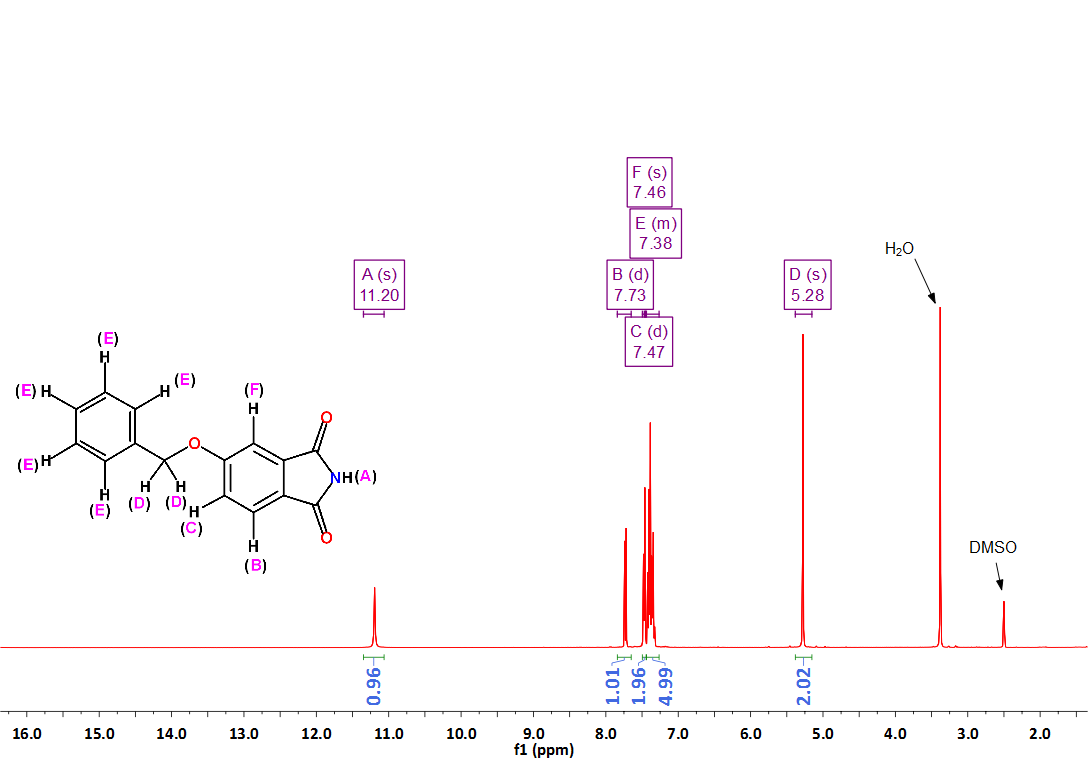
**

**
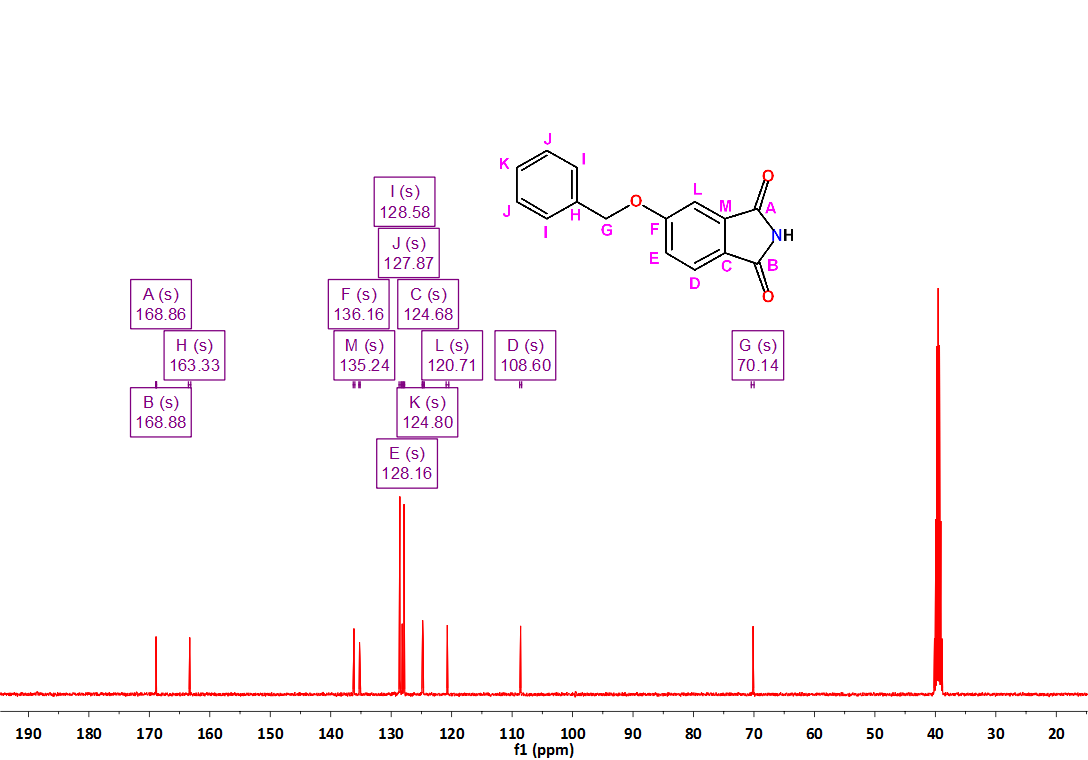
**

**4-Phenylphthalimide (4g)**

**
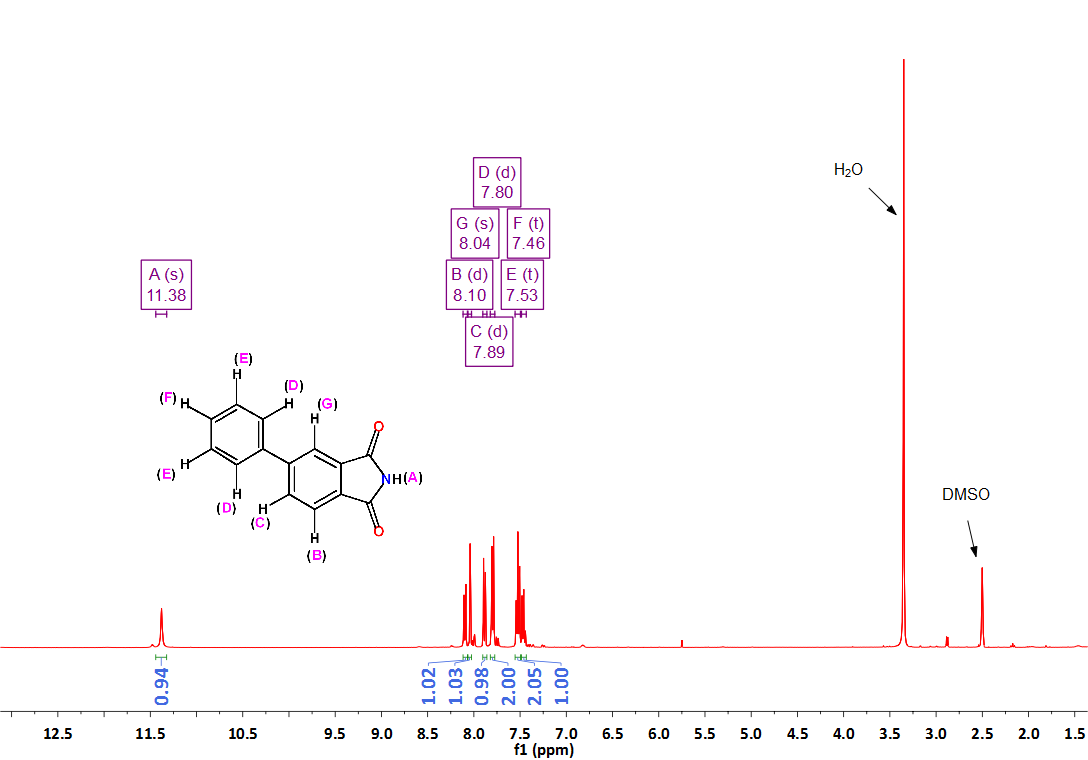
**

**
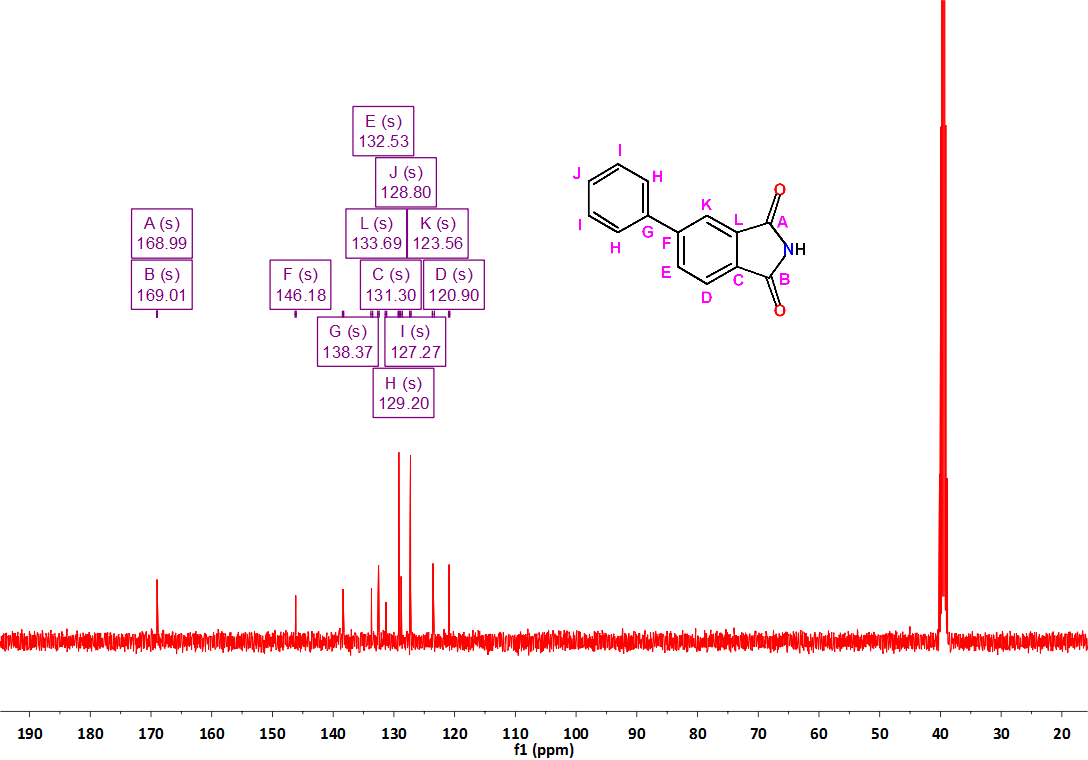
**

**4-Bromophthalimide (4h)**

**
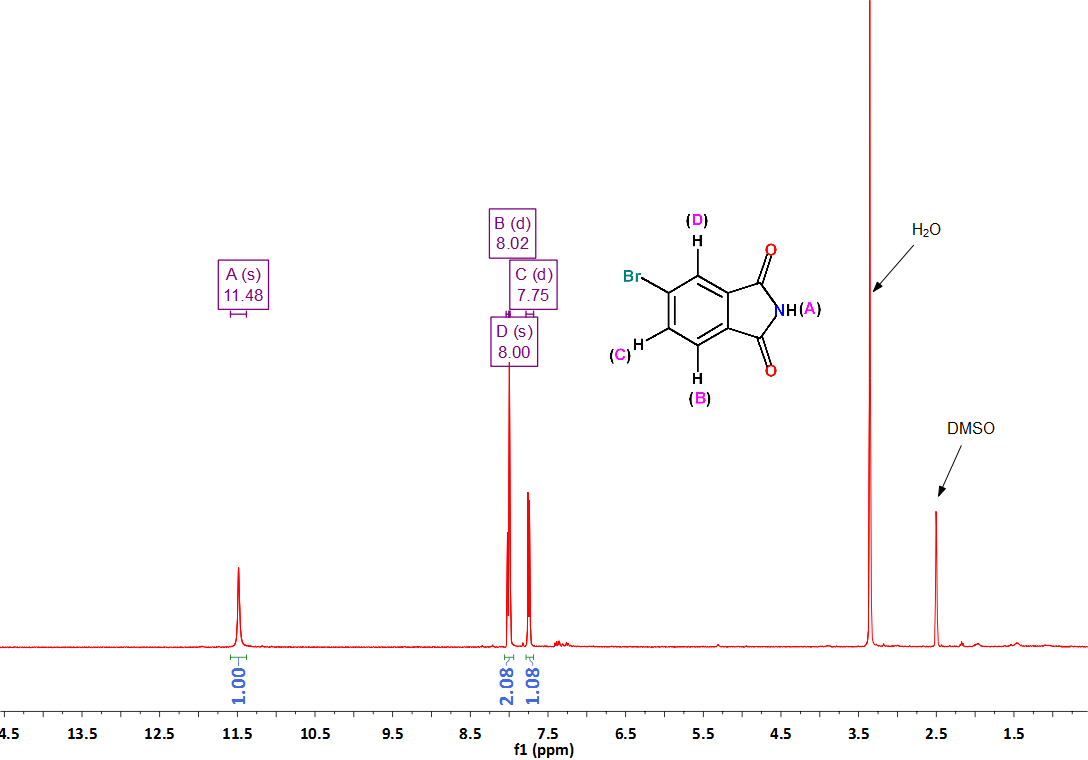
**

**
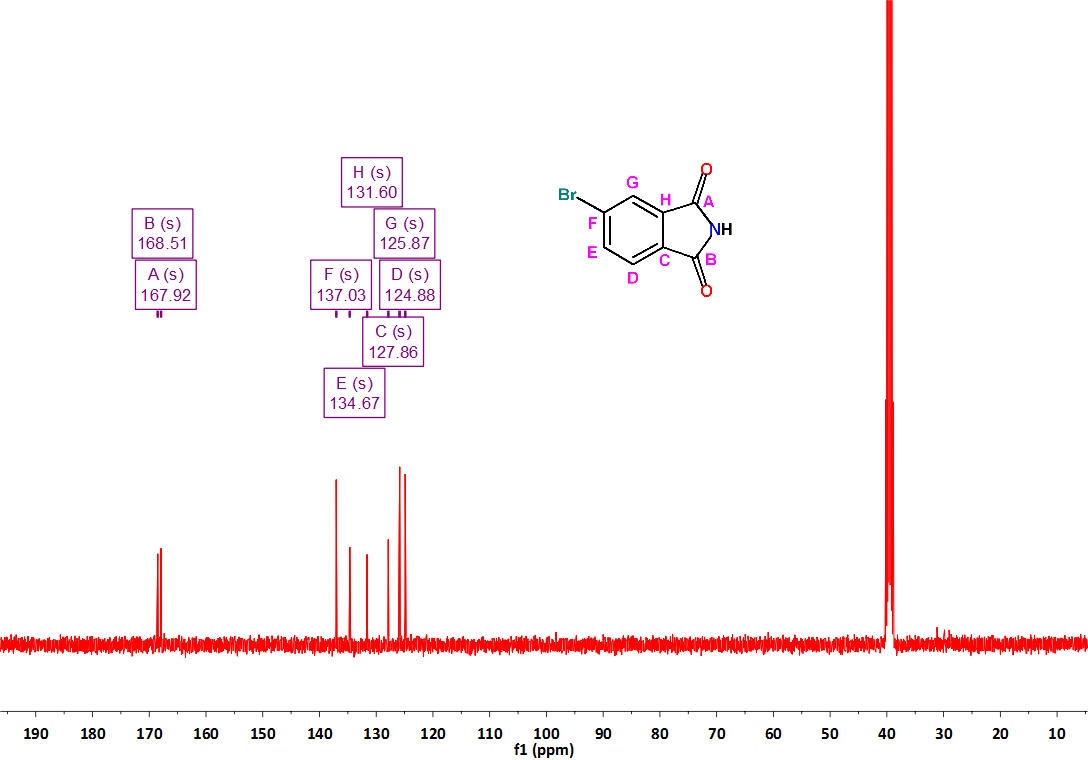
**

**4-Chlorophthalimide (4i)**

**
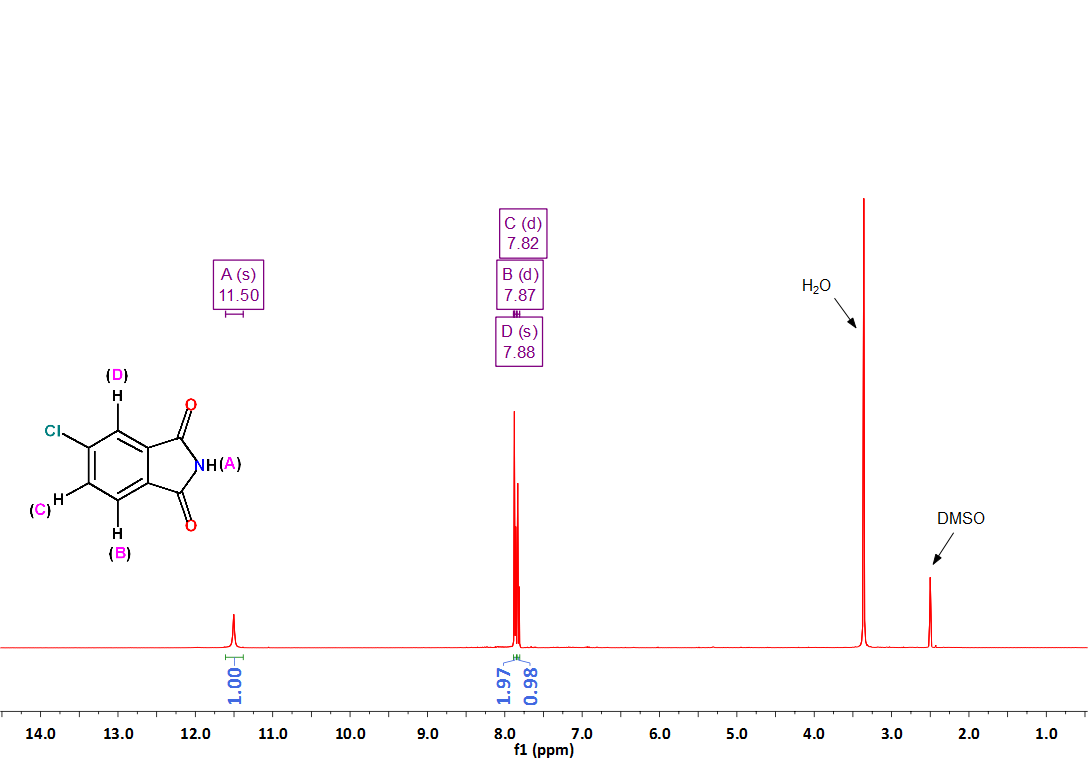
**

**
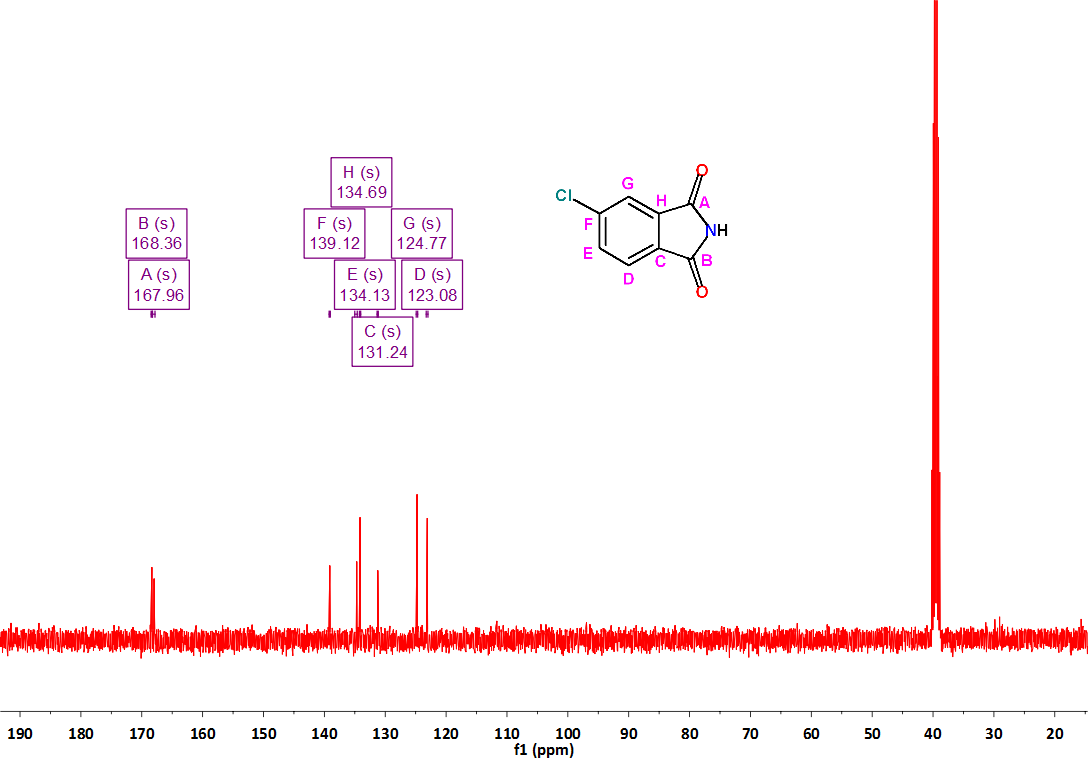
**

**4-Fluorophthalimide (4j)**

**
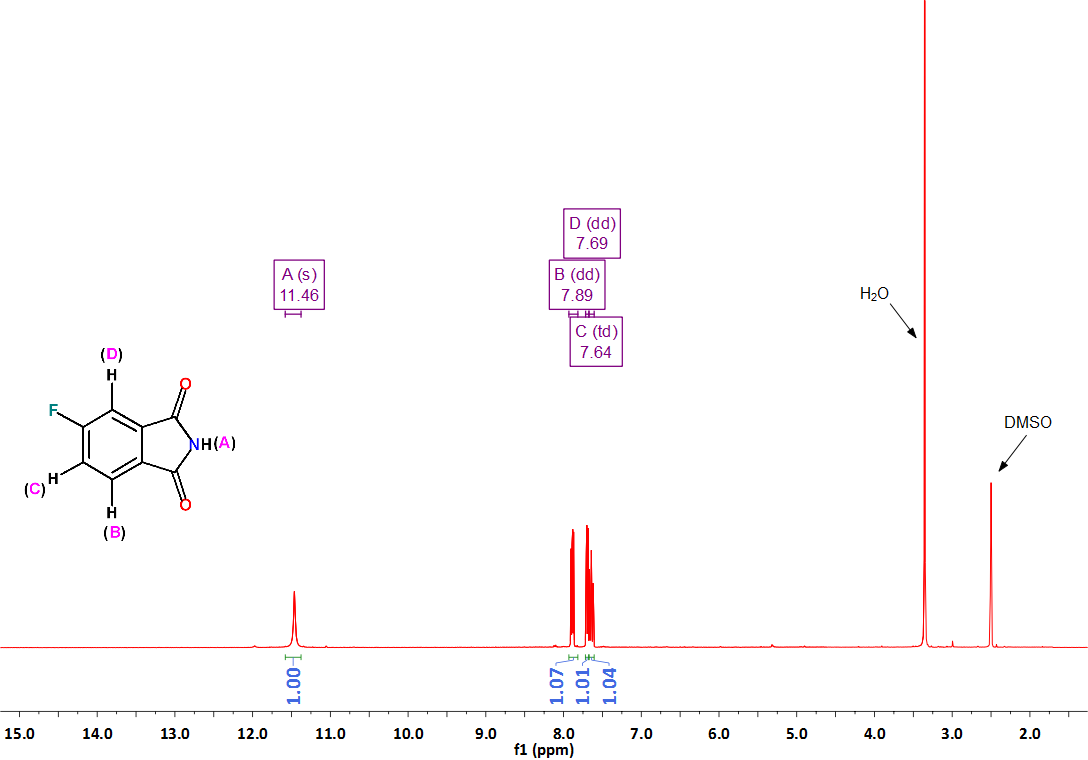
**

**
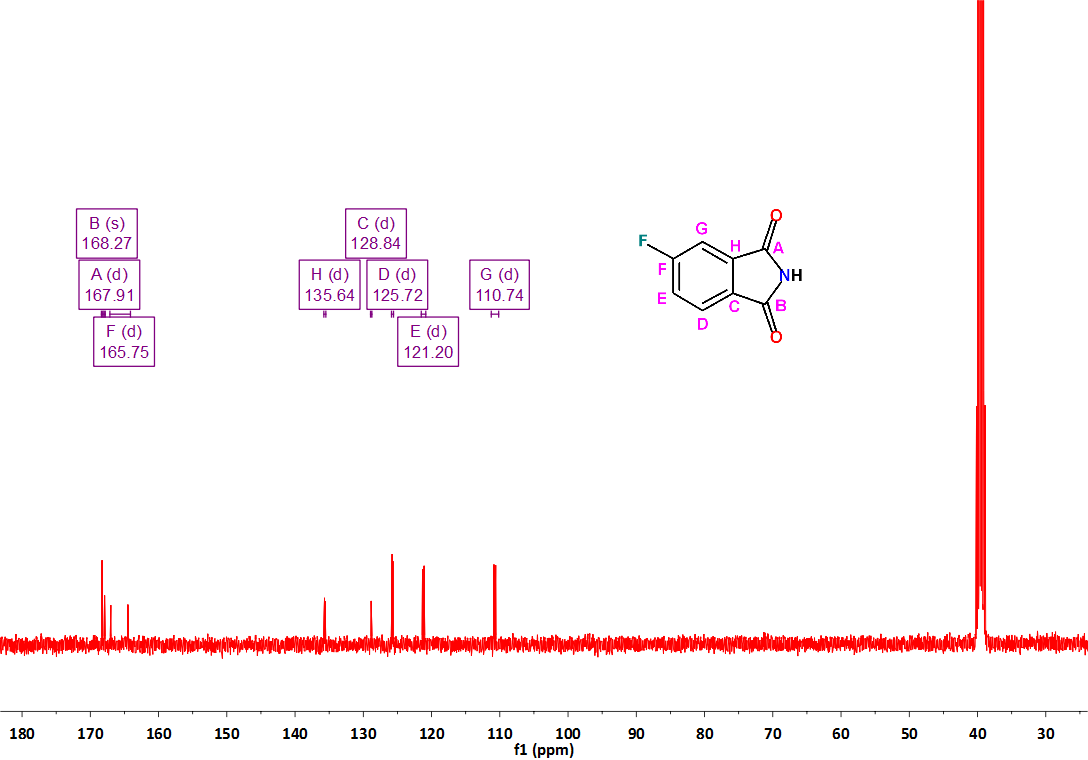
**

**4-Cyano-phthalimide (4k)**

**
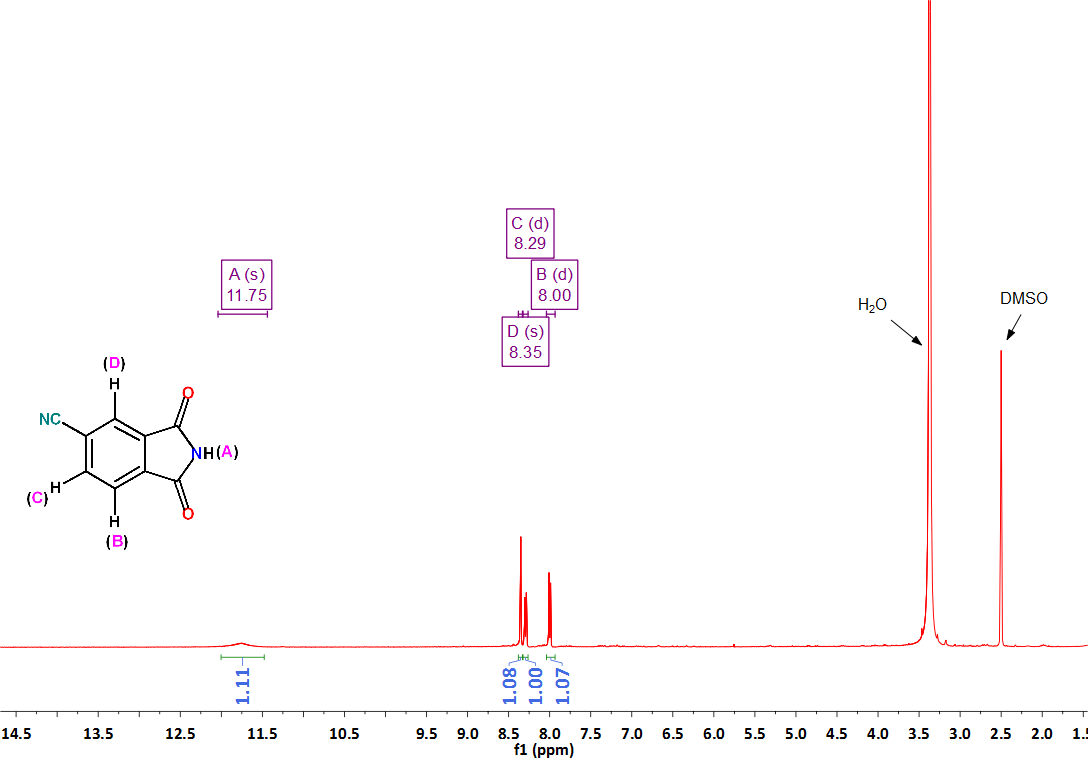
**

**
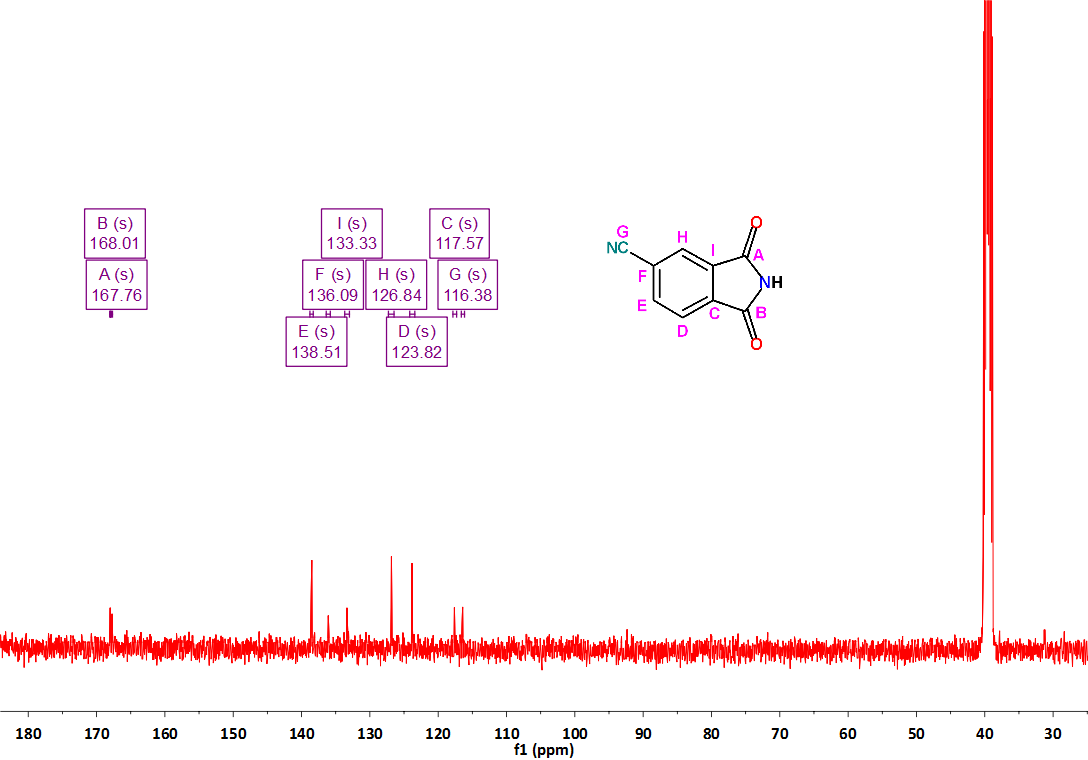
**

**4-Nitro-phthalimide (4l)**

**
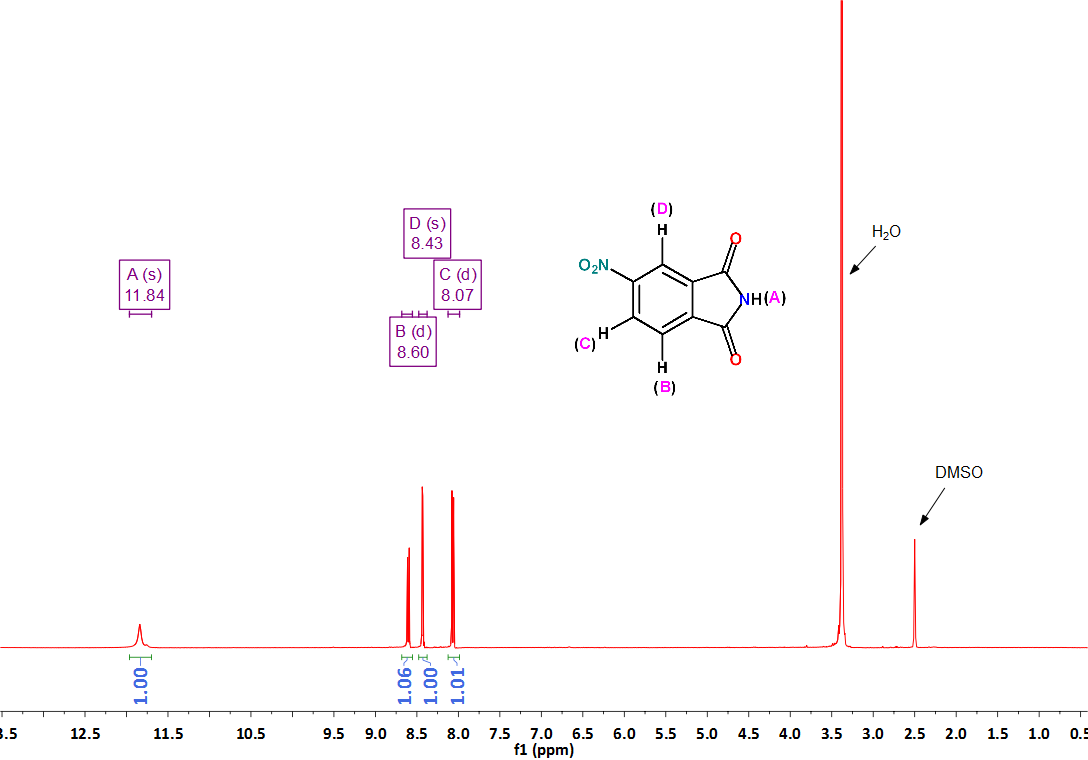
**

**
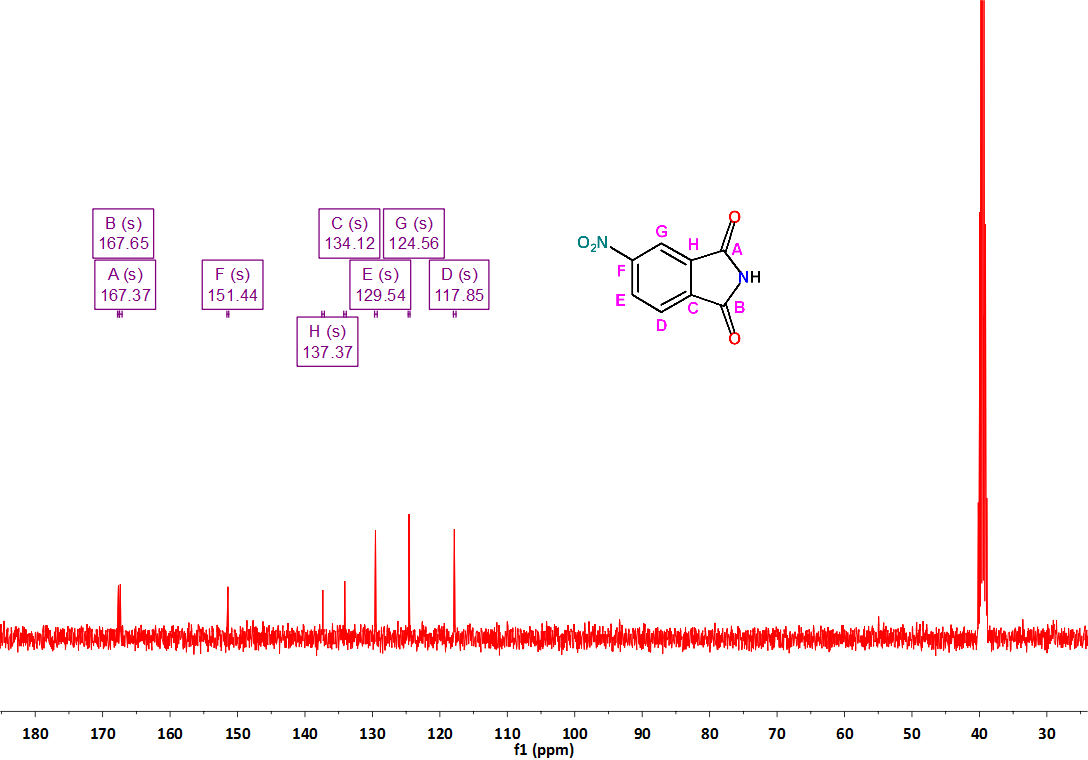
**

**4,5-Dimethoxyl-phthalimide (4m)**

**
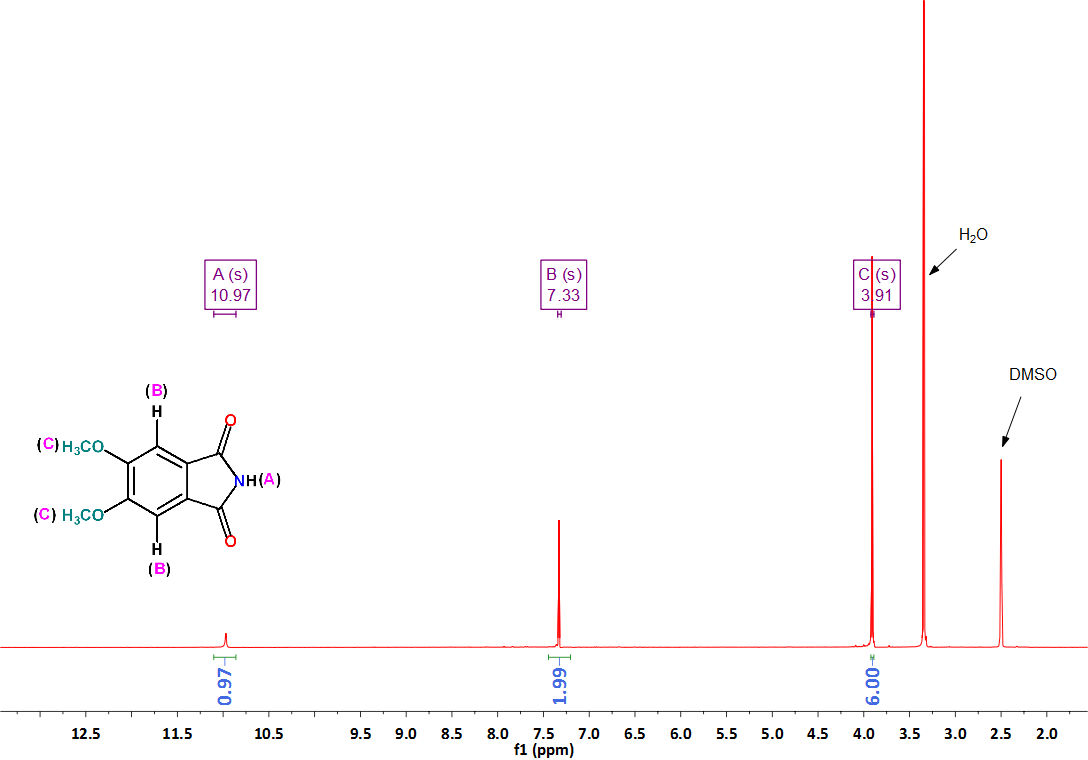
**

**
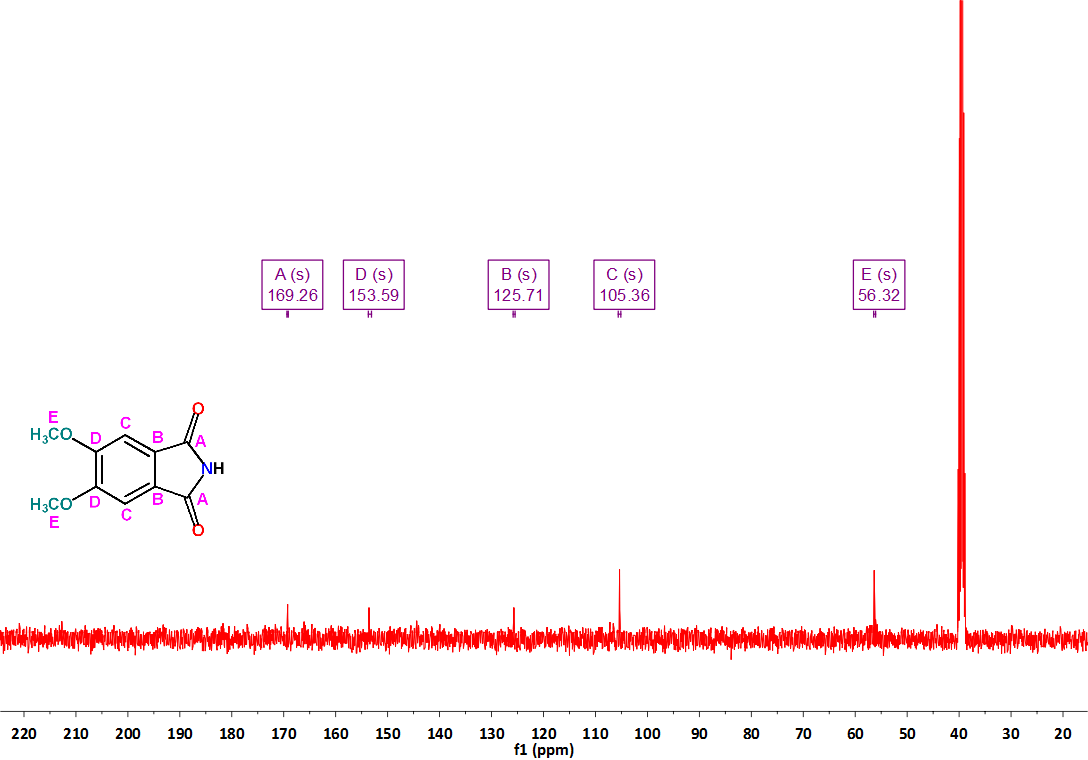
**

**7,8-dihydro-1H-furo[3,2-e]isoindole-1,3(2H)-dione (4n)**

**
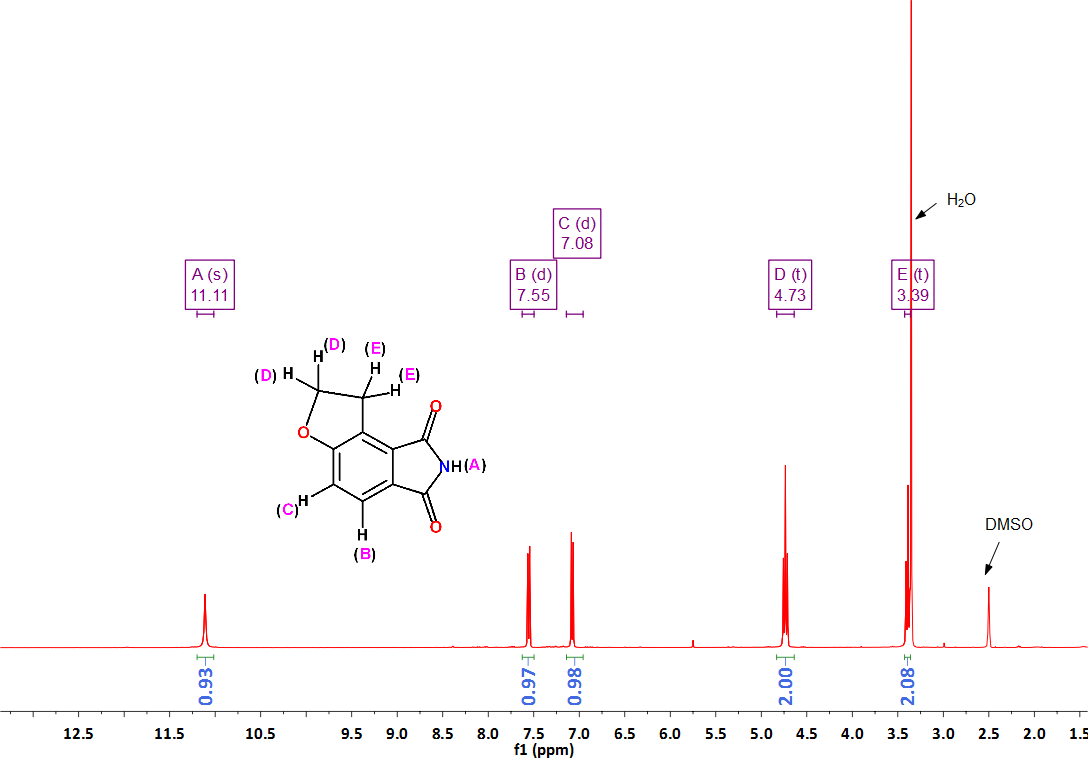
**

**
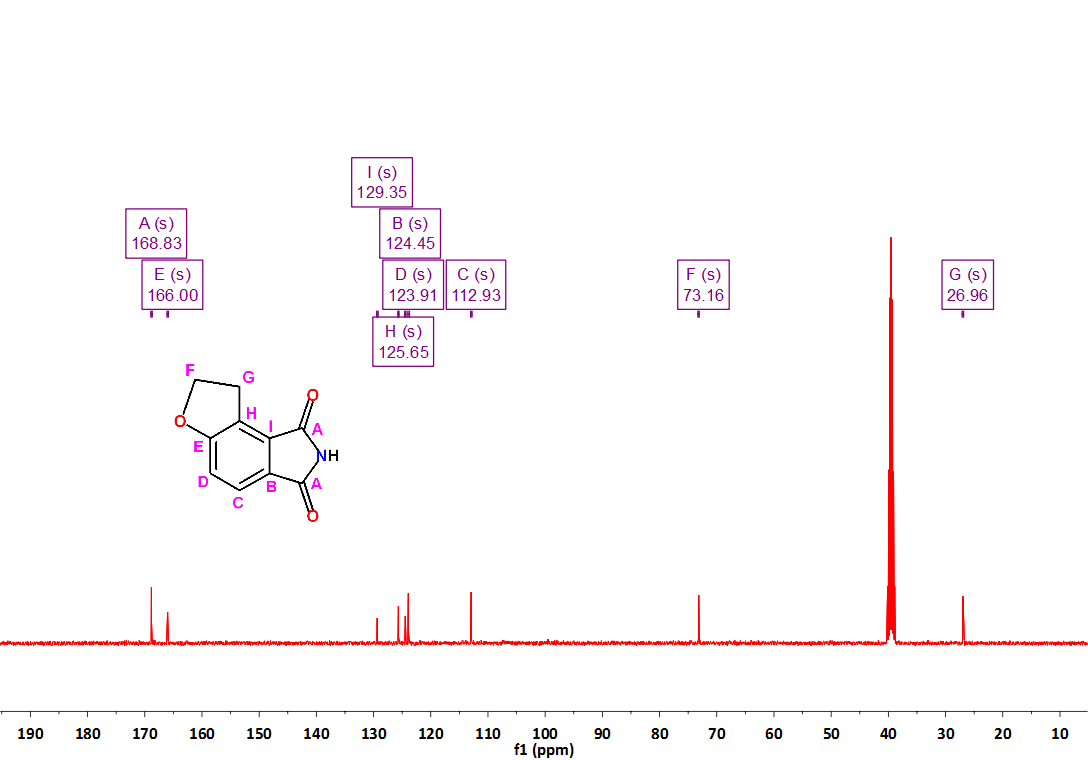
**
